# HIV vaccine candidate efficacy in female macaques mediated by cAMP-dependent efferocytosis and V2-specific ADCC

**DOI:** 10.1038/s41467-023-36109-8

**Published:** 2023-02-02

**Authors:** Massimiliano Bissa, Sohyoung Kim, Veronica Galli, Slim Fourati, Sarkis Sarkis, Anush Arakelyan, Isabela Silva de Castro, Mohammad Arif Rahman, Saori Fujiwara, Monica Vaccari, Jeffrey A. Tomalka, James D. Stamos, Luca Schifanella, Giacomo Gorini, Ramona Moles, Anna Gutowska, Guido Ferrari, Alexei Lobanov, David C. Montefiori, George W. Nelson, Margaret C. Cam, Marita Chakhtoura, Elias K. Haddad, Melvin N. Doster, Katherine McKinnon, Sophia Brown, David J. Venzon, Hyoyoung Choo-Wosoba, Matthew W. Breed, Kristin E. Killoran, Joshua Kramer, Leonid Margolis, Rafick P. Sekaly, Gordon L. Hager, Genoveffa Franchini

**Affiliations:** 1 Animal Models and Retroviral Vaccines Section, National Cancer Institute, Bethesda, MD USA; 2 Laboratory of Receptor Biology and Gene Expression, National Cancer Institute, National Institutes of Health, Bethesda, MD USA; 3 Department of Pathology, Case Western Reserve University, Cleveland, OH USA; 4 Department of Pathology, Emory University, Atlanta, GA USA; 5 Section on Intercellular Interactions, Eunice Kennedy-Shriver National Institute of Child Health and Human Development, National Institutes of Health, Bethesda, MD USA; 6 Tulane National Primate Research Center, Tulane University, Covington, LA USA; 7 Division of Surgical Sciences, Duke University School of Medicine, Durham, NC USA; 8 Collaborative Bioinformatics Resource, Center for Cancer Research, National Cancer Institute, Bethesda, MD USA; 9 Department of Medicine, Drexel University College of Medicine, Philadelphia, PA USA; 10 Vaccine Branch Flow Cytometry Core, National Cancer Institute, Bethesda, MD USA; 11 Biostatistics and Data Management Section, Center for Cancer Research, National Cancer Institute, Bethesda, MD USA; 12 Laboratory Animal Sciences Program, Leidos Biomedical Research Inc., Frederick National Laboratory, Frederick, MD USA

**Keywords:** Vaccines, HIV infections, Antibodies, Phagocytes

## Abstract

The development of an effective vaccine to protect against HIV acquisition will be greatly bolstered by in-depth understanding of the innate and adaptive responses to vaccination. We report here that the efficacy of DNA/ALVAC/gp120/alum vaccines, based on V2-specific antibodies mediating apoptosis of infected cells (V2-ADCC), is complemented by efferocytosis, a cyclic AMP (cAMP)-dependent antiphlogistic engulfment of apoptotic cells by CD14^+^ monocytes. Central to vaccine efficacy is the engagement of the CCL2/CCR2 axis and tolerogenic dendritic cells producing IL-10 (DC-10). Epigenetic reprogramming in CD14^+^ cells of the cyclic AMP/CREB pathway and increased systemic levels of miRNA-139-5p, a negative regulator of expression of the cAMP-specific phosphodiesterase *PDE4D*, correlated with vaccine efficacy. These data posit that efferocytosis, through the prompt and effective removal of apoptotic infected cells, contributes to vaccine efficacy by decreasing inflammation and maintaining tissue homeostasis.

## Abstract (web summary)

HIV vaccine development can be aided by knowledge of correlates of protection. Here the authors identify engagement and reprogramming of tolerogenic CD14^+^ myeloid cells mediating a spatiotemporal balance of pro- and anti-inflammatory responses, as correlates of efficacy in female macaques vaccinated with the DNA/ALVAC/gp120/Alum platform.

## Introduction

Vaccination with the HIV clades B/A/E recombinant canarypox–derived poxvirus vector (ALVAC) and bivalent clade AE/B gp120-envelope proteins in alum significantly reduced the risk of HIV acquisition in the RV144 HIV Phase III vaccine trial in Thailand^1^. A similar study of SIV_mac251_ in the macaque model confirmed the efficacy of this vaccine regimen and, in both humans and macaques, identified antibodies to SIV/HIV variable region 2 (V2), CD4 cells, and Antibody Dependent Cell Cytotoxicity (ADCC) as correlates of risk of HIV/SIV acquisition^2–6^. The relevance of the SIV_mac251_ model has been further substantiated by its ability to predict the lack of efficacy of the HVTN-702 HIV vaccine trial in South Africa, which used an ALVAC-HIV clade C based vaccine together with boosts of gp120 formulated with MF59, rather than alum adjuvant, in a population at high risk (4% incidence in females) of HIV acquisition^7^. Notably, the SIV-based ALVAC vaccine followed by the gp120 boost in alum reduced the risk of SIV_mac251_ acquisition in macaques with an estimated 20-30% decreased risk of virus acquisition at each challenge. However, a simultaneous study testing an SIV-based ALVAC vaccine followed by more inflammatory gp120/ MF59 boosts demonstrated no vaccine efficacy^2^. Strikingly, immunization of macaques with an Ad26-based vaccine followed by ALVAC/gp120/alum boost interfered with vaccine efficacy by establishing a durable pro-inflammatory landscape^3^. In humans, a prime/boost vaccine regimen using Ad26.Mos4.HIV and gp140 formulated with alum was not efficacious in the HVTN-705/Imbokodo HIV vaccine trial^8^.

The macaque model of SIV infection has therefore proven itself as a reliable parallel for the mechanics of HIV infection in humans. In addition to reproducing the immune correlates of risk observed in human trials, pre-clinical studies in the SIV_mac251_ model have been instrumental in uncovering an unexpected role of CD14^+^ cells as strong and reproducible correlates of risk of SIV acquisition^2–4^. CD14 is expressed on a heterogenous population of myeloid cells including classical and intermediate monocytes and tolerogenic dendritic cells^9–11^. Here, we investigated the phenotype, epigenome, and function of CD14^+^ cells in two consecutive studies following vaccination of macaques with the SIV-based DNA/ALVAC/gp120/alum regimen using reduction of per/exposure risk of mucosal SIV_mac251_ acquisition as a surrogate of vaccine efficacy.

First, we contrasted vaccine responses in young and old macaques since aging is associated with functional impairment of CD14^+^ cells^12–16^ and found that in older animals the vaccination did not significantly delay the risk of SIV acquisition, possibly due to high levels of type II interferon, associated with decreased engagement of the anti-inflammatory CCL2/CCR2 axis^17^. Second, by analyzing the CD14^+^ cell epigenome in young vaccinated animals one week following immunization, we uncovered that changes in chromatin accessibility of the cyclic AMP (cAMP)-responsive transcription factor *CREB1* correlated with V2-specific ADCC and vaccine efficacy. In addition, vaccination-induced miR-139-5p, a negative regulator of cAMP-specific phosphodiesterase *PDE4D* expression, correlated with vaccine efficacy in young animals. Third, we found that tolerogenic dendritic cells producing IL-10 (DC-10) and efferocytosis, a cAMP-dependent clearance of apoptotic cells orchestrated by IL-10 that epigenetically reprograms monocytes, correlated with each other and constitute correlates of vaccine efficacy. We conclude that the vaccine-mediated engagement of the anti-inflammatory CCL2/CCR2 axis and DC-10 is critical for the induction of IL-10-mediated efferocytosis, an essential effector response that complements V2-specific ADCC and reduces inflammation.

## Results

### Efficacy of DNA/ALVAC/gp120 candidate vaccine is durable in young macaques

**Fig. 1:**
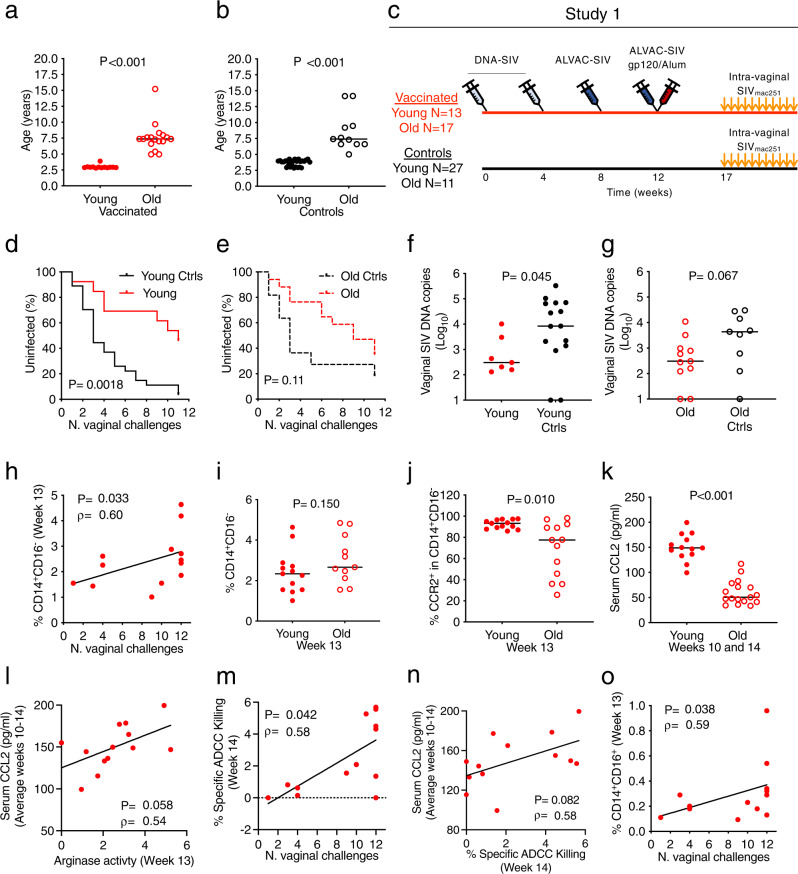
Study design, vaccine efficacy, and monocytes. **a**, **b** Age of vaccinated (**a**; *p* = 0.00000002) and control (**b**; *p* = 0.0000000017) animals. **c** Schematic study design of Study 1 with immunization schedule (weeks 0–12) and SIV_mac251_ challenges (weeks 17–27). **d**, **e** SIV_mac251_ acquisition. The number of intravaginal exposures before infection was assessed in (**d**) *n* = 13 young and (**e**) *n* = 17 old animals relative to their *n* = 27 young and *n* = 11 old control animals (Log-rank Mantel-Cox test). **f**, **g** Log_10_ of SIV-DNA copies in vaginal mucosa at 2–3 weeks after infection in (**f**) *n* = 7 vaccinated and *n* = 15 control young animals; and (**g**) *n* = 11 vaccinated and *n* = 9 control old animals. **h** Correlation between the frequency of classical monocytes (CD14^+^CD16^−^HLA-DR^+^ in live cells) at week 13 and the time of acquisition (TOA) in *n* = 13 young animals. **i** Frequency of classical monocytes in *n* = 13 young and *n* = 11 old vaccinated animals (week 13). **j** Classical monocytes expressing CCR2 (CCR2^+^ in CD14^+^CD16^−^ cells) in *n* = 13 young and *n* = 13 old vaccinated animals (week 13). **k** Serum CCL2 levels (pg/ml) in *n* = 13 young and *n* = 17 old vaccinated animals (average weeks 10 and 14; *p* = 0.0000001). **l** Correlation between the arginase activity measured in plasma at week 13 and the average of the serum CCL2 levels (pg/ml) at weeks 10 and 14 in *n* = 13 young vaccinated animals. **m**, **n** Correlations between (**m**) the average of adjusted specific ADCC killing of SIV_mac251_-infected cells at week 14 assessed at different plasma dilutions and the TOA, or (**n**) the average of the serum CCL2 levels (pg/ml) at weeks 10 and 14 in *n* = 13 young vaccinated animals. **o** Correlation between the frequency of intermediate monocytes (CD14^+^CD16^+^HLA-DR^+^ in live cells) at week 13 and the TOA in *n* = 13 young animals. Comparisons: **a**, **b**, **f**, **g**, **i**–**k** Two-tailed Mann–Whitney *t*-test with median. Correlation analyses: **h**, **l**–**o** two-tailed Spearman correlation test and simple linear regression. Displayed p values are unadjusted. Source data are provided in the Source Data file.

One of the aims of the current study was to investigate the role of monocytes in detail since their frequency has been associated with the efficacy of the DNA/ALVAC/gp120/alum vaccine in prior studies^3^. Since monocyte function decreases with age^14–18^, we designed a study (Study 1) using animals differing in age to better contrast monocyte responses. We vaccinated or left naïve macaques differing in age (Mean [SD] defined here for descriptive purpose as, young: 3.45 [0.545]; and old animals: 7.98 [2.60] years; Fig. 1a–c). Specifically, 13 young and 17 old macaques were primed twice with DNA-SIV (*gag*, *pol*, and *env*; weeks 0 and 4) and boosted with ALVAC-SIV alone (week 8) or in combination with SIV_mac251_ and SIV_smE660_ proteins adjuvanted in alum Alhydrogel (week 12). Vaccinated and naïve animals were then intravaginally exposed to 11 weekly doses of SIV_mac251_. We found no difference in per-exposure risk of vaginal SIV_mac251_ acquisition for the 27 young and 11 old naïve control animals, indicating no association of susceptibility to SIV_mac251_ infection with aging (*p* = 0.50; HR = 1.25 with the old control group as reference; Supplementary Fig. S1a). Vaccination reduced the risk of SIV_mac251_ acquisition when compared to age-matched controls in young animals (average per exposure of 71%; *p* = 0.0018; HR = 0.31 with the young control group as reference; Fig. 1d), but showed much weaker decreased acquisition in old macaques (*p* = 0.11; HR = 0.52 with the old control group as reference; Fig. 1e). A decrease in SIV-DNA copies in vaginal mucosa was more pronounced in young vaccinated animals (*p* = 0.045; medians for the vaccinated and control groups = 305 and 8384, respectively; Fig. 1f) than old (*p* = 0.067; medians for the vaccinated and control groups = 306 and 4343, respectively; Fig. 1g), with transient protection from CD4^+^ T-cell loss occurring in young animals at week 2 (*p* = 0.012), and in old animals at week 10 post-infection (*p* = 0.027; Supplementary Fig. S1b, c) when compared to aged-matched infected controls. No significant difference in plasma virus levels was observed over time in vaccinated groups compared to their controls (Supplementary Fig. S1d, e). Six of the thirteen vaccinated young animals that remained uninfected (46%) were re-exposed to a second round of 11 weekly SIV_mac251_ challenges, starting 7 months from the last immunization, and maintained a significant reduction in the risk of SIV_mac251_ acquisition (*p* = 0.020; HR = 0.33 with the young control group as reference; Supplementary Fig. S1f). Together, these data demonstrated durable vaccine efficacy in young animals.

### CCL2/CCR2 axis and ADCC against SIV_mac251_ infected cells are associated with vaccine efficacy

We characterized monocyte populations phenotypically by flow cytometry. Our analyses of monocyte subsets demonstrated a correlation of decreased risk of virus acquisition with the frequency of CD14^+^CD16^−^ classical monocytes measured one week following the last immunization at week 13 in young (*ρ* = 0.60, *p* = 0.033; Fig. 1h) but not old animals (*ρ* = −0.19, *p* = 0.575; Supplementary Fig. S2a), even though classical monocyte frequency after immunization did not differ between the groups (*p* = 0.150; Fig. 1i). In old animals, a smaller proportion of classical monocytes expressed the homing marker C-C chemokine Receptor type 2 (CCR2; *p* = 0.010; Fig. 1j). In addition, at the end of immunization, C-C motif Chemokine Ligand 2 (CCL2) serum levels were higher in young macaques (*p* < 0.001; Fig. 1k and Supplementary Fig. S2b), likely reflecting an age-dependent response to vaccination. Since engagement of the CCR2/CCL2 axis has been associated with polarization to M2 monocytes producing the immune suppressive enzyme arginase^19,20^, we measured arginase activity in the plasma of vaccinated animals. Immunization resulted in lower median arginase activity in old animals compared to young (*p* = 0.096; Supplementary Fig. S2c), and arginase activity correlated with serum CCL2 levels in young (*ρ* = 0.54, *p* = 0.058; Fig. 1l**)** but not old macaques (*ρ* = 0.15, *p* = 0.570; Supplementary Fig. S2d). ADCC, a central correlate of decreased risk of HIV/SIV acquisition for the ALVAC-based vaccine modality^5,6^, measured against SIV_mac251_-infected cells did not differ in young and old animals (*p* = 0.481; Supplementary Fig. S2e), and correlated with reduced risk in young but not old animals (*ρ* = 0.58, *p* = 0.042; Fig. 1m; and *ρ* = 0.33, *p* = 0.188; Supplementary Fig. S2f, respectively). In addition, the average CCL2 serum levels at weeks 10 and 14 correlated with ADCC at week 14 in young but not old animals (*ρ* = 0.58, adjusted *p* = 0.082; Fig. 1n; and *ρ* = 0.007, *p* = 0.981; Supplementary Fig. S2g, respectively). All together, these data may indicate that vaccine-induced engagement of the CCL2/CCR2 axis^17^ in young animals influences ADCC, although further studies will be required to verify this hypothesis. Interestingly, in the current shortened vaccine regimen (12-weeks), and in contrast to the 24-week regimen^2,3^, we found that CD14^+^CD16^+^ intermediate monocyte frequency is also correlated with delayed viral acquisition (*ρ* = 0.59, *p* = 0.038; Fig. 1o). Neutralizing antibody titers to SIV Tier1A and the SIV_mac251_ challenge virus were higher in young animals, but titers to both SIV Tier1A and Tier1B correlated with increased virus acquisition in young, vaccinated animals (Supplementary Table S1). These data support that the mechanisms of protection by the DNA/ALVAC/gp120/alum vaccine regimen are distinct from neutralization.

### Age-associated pro-inflammatory cytokine profile skews engagement of the CCL2/CCR2 axis

In order to explore differences in response to vaccination between young and old macaques, we analyzed plasma cytokines and chemokines prior to and following immunization. Analyses demonstrated higher TGF-β1 and TGF-β2 in young animals than old and higher IFN-γ, IL-10, IL-1β, and CCL3 in old than young animals at baseline, consistent with observations in elderly humans^13^, and equivalent levels of CCL2 in both age groups (Supplementary Fig. S3a and Supplementary Table S2). Vaccination decreased plasma TGF-β1 and TGF-β2 to a larger extent in young than old macaques, which retained higher levels of IFN-γ and IL-8 than young animals one week after the last immunization. In contrast, in young animals CCL2 and IL-6 were significantly higher after vaccination (Supplementary Fig. S3b and Supplementary Table S2). Notably, the level of IFN-γ, a cytokine that induces pro-inflammatory monocytes^21^, negatively correlated with the vaccine-induced plasma CCL2 levels in old macaques (*ρ* = −0.49, *p* = 0.047; Supplementary Fig. S3c). Similarly, plasma IL-8 levels after DNA prime (week 4) correlated with increased risk of SIV_mac251_ acquisition only in old animals (*ρ* = −0.56, *p* = 0.022; Supplementary Fig. S3d). In vitro LPS-stimulation of PBMCs of young and old animals showed a lower production of different cytokines/chemokines in PBMCs from old animals (Supplementary Fig. S3e–g and Supplementary Table S3), confirming that aging skews monocyte responsiveness to TLR stimulation, as observed in humans^18^. These data suggest that the high levels of IFN-γ in old animals likely contributed to the decreased engagement of the CCL2/CCR2 axis^20^ and lower vaccine efficacy, although further studies would need to be conducted to validate the hypothesis.

### BioAge module promoting IFN type II responses and *ZC3H7A* expression associated with decreased vaccine efficacy in young animals

**Fig. 2:**
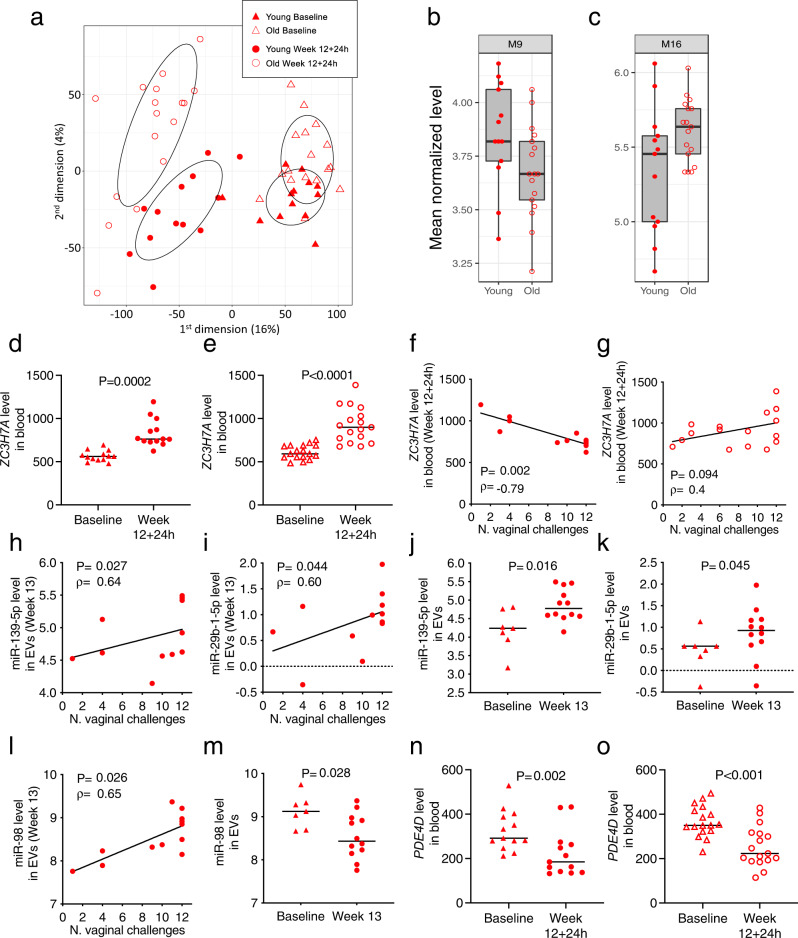
Transcriptome and extracellular vesicular microRNAs. **a** Multidimensional scaling plot summarizing gene expression. Different responses to vaccination identified in *n* = 13 young and *n* = 17 old animals. Differences were present between baseline and 24 h following vaccination (first dimension, Wilcoxon test, *p* = 3.18 × 10^−11^) and between young and old groups (second dimension, Mann–Whitney, *p* = 8.77 × 10^−*6*^). **b**, **c** Expression levels of genes in BioAge modules M9 and M16 associated with age and risk of SIV_mac251_ acquisition in *n* = 13 young and *n* = 17 old animals (week 12 + 24 h). The median, 95% CI, and first and third quartiles are displayed. **d**, **e** Expression of *ZC3H7A* gene at baseline and 24 h following vaccination in (**d**) *n* = 13 young and (**e**; *p* = 0.00003) *n* = 17 old animals. **f**, **g** Correlations between the expression of *ZC3H7A* at 24 h following vaccination and the Time of Acquisition (TOA) in (**f**) *n* = 13 young and (**g**) *n* = 17 old vaccinated animals. **h**, **i**, **l** Correlations between the expression of **h** miR-139-5p, **i** miR-29b-1-5p, or **l** miR-98 in EVs (week 13) and TOA in *n* = 12 young animals. **j**, **k**, **m** Normalized expression of **j** miR-139-5p, **k** miR-29b-1-5p, and **m** miR-98 in *n* = 7 young animals at baseline and *n* = 12 young vaccinated animals at week 13. **n**, **o** Expression of Phosphodiesterase-4D (*PDE4D*) at baseline and 24 h following the last boost in the (**n**) *n* = 13 young and (**o**; *p* = 0.00066) *n* = 17 old animals. Comparisons: **d**, **e**, **n**, **o** two-tailed Wilcoxon signed rank or **j**, **k**, **m** two-tailed Mann–Whitney tests with median. Correlation analyses: **f**–**i**, **l** two-tailed Spearman correlation tests and simple linear regression. Displayed *p* values are unadjusted. Source data are provided in the Source Data file and at GEO Series accession numbers GSE188901 and GSE188575.

To better contrast the immune responses elicited by vaccination in young and old animals, we conducted transcriptome analyses on whole blood both at pre-vaccination and 24 h following final immunization. Analyses demonstrated a significant difference in gene expression in young and old animals before and after immunization (explaining 4% of variance in gene expression; *p* = 8.77 × 10^−6^, Mann–Whitney test between the second multidimensional scaling dimension and the animals’ age groups; Fig. 2a). Previous work characterizing gene expression changes associated with aging in humans (BioAge) identified 20 modules of co-expressed genes correlating with age^22^. A similar analysis identified that the expression of genes clustered in modules 9 and 10, respectively involved in the metabolic pathways of fatty acid biosynthesis and amino acid metabolism and both associated with young age in humans, correlated here with decreased risk of SIV_mac251_ acquisition in young vaccinated macaques (adjusted *p* = 0.004 and *p* = 0.030, respectively, GSEA analysis; Supplementary Fig. S4a and Supplementary Table S4). In contrast, the increased expression of transcripts clustered in modules 16 and 17, associated with old age in humans and which respectively promote inflammation and type II interferon signaling, were associated with increased risk of SIV_mac251_ acquisition in young vaccinated macaques (adjusted *p* = 0.026 and *p* = 0.032, respectively, GSEA analysis; Supplementary Fig. S4a and Supplementary Table S4). Comparison of macaque gene expression to the human age-associated modules showed higher expression of genes belonging to BioAge module M9 (*p* = 0.046) and lower expression of genes belonging to module M16 (*p* = 0.062) in young animals (Fig. 2b, c).

These data are consistent with the ex vivo finding of high, sustained levels of IFN-γ in old animals reported in Supplementary Fig. S3a, b. Further analyses of gene expression before or after vaccination identified several genes correlated with risk of virus acquisition in young and old macaques (Supplementary Table S5a). However, correction for False Discovery Rate (FDR) of vaccine-induced genes (fold-changes) narrowed it down to a single gene, *ZC3H7A*, whose expression was increased by vaccination in both young and old animals (*p* = 0.0002 and *p* < 0.0001 respectively; Fig. 2d, e) and its expression at 24 h following final vaccination correlated with increased risk of SIV_mac251_ acquisition in young but not in old animals (*ρ* = −0.79, *p* = 0.002 and *ρ* = 0.4, *p* = 0.094, respectively; Fig. 2f, g and Supplementary Table S5b). *ZC3H7A*, whose expression is upregulated following stimulation of primary bone marrow-derived macrophages in vitro^23^ and during Th2 cell activation^24^, belongs to the class III CCCH-zinc finger RNA-binding protein that regulates miRNA biogenesis through its recognition of miRNA hairpin sequences, suggesting its function includes miRNA processing regulation^25^.

### miR-139-5p and *CREB*-responsive genes correlate with vaccine efficacy

The finding that *ZC3H7A* is a regulator of miRNA processing raised the hypothesis that miRNAs may play a role in vaccine efficacy and prompted us to sequence and quantitate miRNA content in plasma extracellular vesicles (EVs)^26^ obtained from young and old animals at the end of immunization and some of the young animals prior to vaccination. We found 109 miRNAs that differed significantly between age groups at the end of immunization (Supplementary Fig. S4b and Supplementary Table S6). Several miRNAs correlated with risk of virus acquisition in young and old animals, but none remained significant following correction for FDR (Supplementary Tables S7a, S8). Nevertheless, we focused on the miRNAs identified in young animals which were modified by vaccination and correlated (unadjusted p-values) with decreased risk of SIV acquisition (Supplementary Table S7b). Two miRNAs, miR-139-5p and miR-29b-1-5p (correlations with risk: *ρ* = 0.64, *p* = 0.027 and *ρ* = 0.60, *p* = 0.044, respectively; Fig. 2h, i) were increased by vaccination (*p* = 0.016 and *p* = 0.045, respectively; Fig. 2j, k) and one, mir-98 (correlation with risk: *ρ* = 0.65, *p* = 0.026; Fig. 2l) was decreased by vaccination (*p* = 0.028; Fig. 2m). MiR-139-5p has several validated targets, including: *CXCR4*, important for egress of CXCR4^+^ cells and monocytes from bone marrow^27,28^ and HIV entry in target T-cells^29^; the cAMP-specific *PDE4D* gene^30^, which decreases CREB1-mediated transcription by degrading cAMP; and the *IGF-1/AKT/Glut1* gene receptor, central for glucose utilization and cellular metabolism^31^. Indeed, whole blood RNA-seq analysis revealed decreased *PDE4D* expression following vaccination in both age groups (young *p* = 0.002 and old *p* < 0.001; Fig. 2n, o). Consistent with decreased *PDE4D*, the expression of cAMP/CREB1-responsive genes was significantly upregulated by vaccination in young and old animals (*p*-values after FDR *p* = 0.010 and *p* = 0.030 respectively; Supplementary Fig. S4c, d for young and old animals, respectively). The validated target of miR-29b-1-5p is the NF-kB pathway^32^; mir-98 has several validated targets, inhibits IL-10 production^33^, modulates M1-M2 polarization of macrophages towards the M1 phenotype^34^, and its CCL2-induced downregulation increases IL-6 production^35^. To confirm the ability of miR-139-5p to modulate *PDE4D* expression, we transfected PBMCs or CD14^+^ cells collected from naïve macaques with miR-139-5p mimic or a negative control, and incubated for 4 h. *PDE4D* relative expression in PBMCs transfected with miR-139-5p normalized for housekeeping gene and *PDE4D* expression in PBMCs transfected with miR negative control decreased after 4-h incubation (*p* = 0.046; Supplementary Fig. S4e). However, the same effect was not seen in the experiment conducted with purified CD14^+^ cells (*p* = 0.739; Supplementary Fig. S4f), suggesting that miRNA139-5p may have an indirect effect on CD14^+^ cells. These data indicate the importance of *CREB1*-mediated transcription in vaccine efficacy, likely assisted by miR-139-5p targeting the cAMP-degrading *PDE4D* gene.

### Increased chromatin accessibility of *CREB1* in CD14^+^ cells associated with vaccine efficacy

**Fig. 3:**
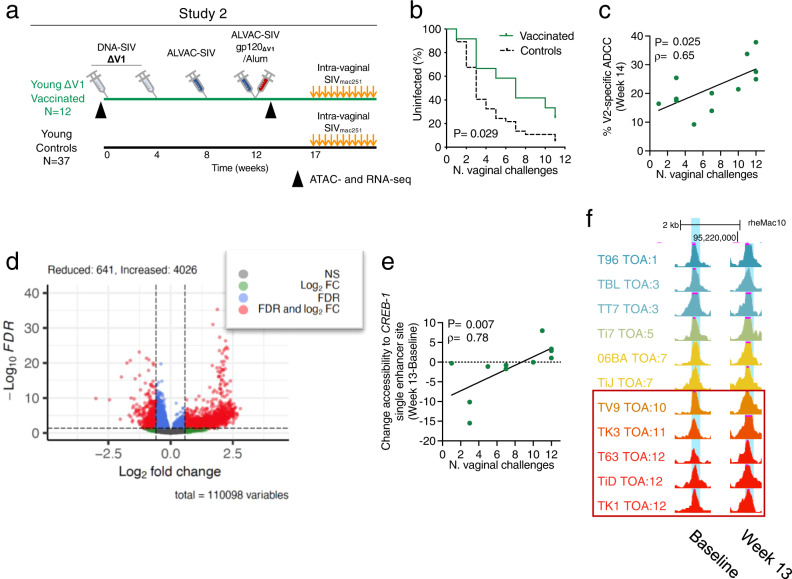
Chromatin accessibility to *CREB1*. V2 specific ADCC and risk of SIV acquisition. **a** Schematic study design with time of immunization (weeks 0–12) or SIV_mac251_ challenges (weeks 17–27) and time of collection of ATAC- and RNA-seq samples (black triangle). **b** SIV_mac251_ acquisition. The number of intravaginal exposures before viral acquisition was assessed in *n* = 12 young animals relative to *n* = 37 historic controls (Log-rank Mantel-Cox test). **c** Two-tailed Spearman correlation and simple linear regression between V2-specific ADCC at week 14 and time of acquisition (TOA) in *n* = 12 animals. **d** Volcano plot showing in red ATAC-sites with significantly (FDR < 0.05) different accessibility at baseline and week 13 and with absolute fold-change higher than 1.5 (Log_2_ = 0.585). Reduced accessibility: 641 sites, Increased: 4026. Blue dots: sites with significantly different accessibility, but lower absolute fold-change. Green dots: sites with not significantly different accessibility, but absolute fold-change higher than 1.5. Gray dots: sites with not significantly different accessibility, but low absolute fold-change. Comparisons performed by two-tailed paired analyses. **e** Two-tailed Spearman correlation and simple linear regression of variation between baseline and week 13 of the intensity of ATAC site downstream *CREB1* TSS accessibility (chr12:95218442-95218735) identified in Supplementary Fig. S6b with TOA in *n* = 11 vaccinated animals (week 13). **f** Visualization of the ATAC site downstream *CREB1* TSS (chr12:95218442-95218735) whose variation between baseline and week 13 correlated with TOA. The ATAC site of each animal is shown at baseline and week 13 for better comparison (*n* = 11 animals). Animals that showed an increase in accessibility following vaccination are indicated by the red box. Animals are colored based on TOA, and those with TOA > 8 are framed. Displayed *p* values are unadjusted. Source data are provided in the Source Data file and at GEO Series accession numbers GSE188879 and GSE189032.

The role of CD14^+^ cells in decreasing the risk of SIV acquisition and the durable vaccine efficacy in young vaccinated macaques indicate the possible induction of long-term innate responses following vaccination. Indeed, studies on tolerogenic CD14^+^ dendritic cells^36^ and trained immunity^37^ suggested the ability of CD14^+^ cells induced by our vaccine strategy to persist in tissues and mediate vaccine efficacy. We hypothesized that the DNA/ALVAC-SIV/gp120/alum vaccine regimen could induce epigenetic reprogramming of CD14^+^ cells and shape their responses to secondary stimulus, such as SIV infection. We designed Study 2 to investigate the epigenetic changes induced by our vaccine strategy and vaccinated twelve young female macaques with a second-generation simplified vaccine regimen using V1-deleted immunogens to favor an α-helix conformation of V2, delivered by the DNA/ALVAC platform^5^ (Fig. 3a). Animals were immunized and then challenged intravaginally similarly as in Study 1. Vaccination reduced the risk of SIV_mac251_ acquisition by 52% (*p* = 0.029; HR = 0.49 with the young control group as reference; Fig. 3b), and SIV_mac251_ acquisition correlated with V2-specific ADCC (V2-ADCC) as previously described^5^ (*ρ* = 0.65, *p* = 0.025; Fig. 3c). RNA-seq and ATAC-seq were performed on CD14^+^ cells isolated by microbead positive selection at baseline and 1 week after the last immunization (week 13).

First, we explored the overall chromatin accessibility in CD14^+^ cells. While the total number of accessible sites significantly decreased between baseline and following vaccination at week 13 (*p* < 0.001; Supplementary Fig. S5a), we refined our assessment to avoid potential errors due to background noise via a paired analysis considering only fold-changes >1.5. We identified 641 and 4032 sites with significantly decreased or increased accessibility, respectively (FDR adjusted *p* < 0.05; Fig. 3d), indicating that vaccination changed the epigenetic landscape of CD14^+^ cells.

We next investigated the role of chromatin accessibility in vaccine efficacy by conducting an unbiased analysis on the overall transcriptome and epigenetic landscape by combining ATAC-Seq and RNA-Seq data analysis using the workflow summarized in Supplementary Fig. S5b. First, we compared the transcriptome at baseline among animals that acquire SIV before or after eight single-virus challenge exposures (Low vs High Time of Acquisition [TOA]) and selected 14,397 genes that were similarly expressed. The threshold TOA of eight challenges was used as a filtering criterion to reduce the large number of genes to a manageable subset of special interest and was selected due to its ability to divide the TOA distribution as close to the median as possible, aligning with a gap in the distribution between the seven observed TOA with values ranging 1–7 and the five ranging 10–12, inclusively. We then compared the expression of the 14,397 genes following vaccination (week 13) in animals with low and high TOA and identified 821 genes that were associated with risk of SIV acquisition only following vaccination. Next, we compared the expression of these 821 genes between baseline and week 13 for both low and high TOA groups. In order to select genes differentially expressed at baseline vs week 13 only in high TOA animals, we identified 136 genes affected in the high TOA group and removed the 79 common genes identified by the same analysis in both High and Low TOA animals. We therefore identified 57 genes (Supplementary Table S9) and analyzed the accessibility surrounding their Transcription Starting Sites (TSSs; +/−250 kb). To identify only ATAC sites that had a potential effect on gene expression, we correlated the variation between the accessibility of 2363 ATAC sites around the 57 TSSs at baseline and week 13 with the variation between the expression of the 57 genes previously identified in the same timeframe. We selected 272 ATAC sites showing a correlation with gene expression with absolute Spearman correlation *ρ*>|0.5|.

To characterize the potential transcription regulators contributing to accessibility change, we performed de novo motif analysis for the 272 ATAC sites using the set of peaks identified in the data as a background set using HOMER motif analysis. The top enriched motif was found in 3.68% of the 272 ATAC sites (*p* = 1e–10) and matched to known motifs PU.1, SPIB, and SPI1 (Supplementary Fig. S6a). The PU.1 transcription factor is required for the development of myeloid-derived dendritic cells^38^. Moreover, in humans, the Sfpil-encoded PU.1 protein is known to recruit CREB-binding proteins^39^, further suggesting its critical role in the CREB pathway and vaccine protection. Since the data pointed to a central role of CREB, we analyzed the vaccine-induced change in the accessibility of the enhancer region surrounding (+/−250 kb) *CREB1* TSS in CD14^+^ cells (variation between baseline and post-vaccination) and identified 43 accessible ATAC sites. The variation in the accessibility of one of the 43 ATAC sites identified in the *CREB1* enhancer region correlated with decreased risk of virus acquisition (*ρ* = 0.78, *p* = 0.007; Fig. 3e, f and Supplementary Fig. S6b), and moderately with the V2-specific ADCC response (*ρ* = 0.56, *p* = 0.076; Supplementary Fig. S6c). Thus, in our studies *CREB1* chromatin accessibility appears to influence vaccine efficacy by shaping the V2-specific ADCC response, consistent with recent observations of RV144 volunteers and non-human primates^40^.

### Activation of CREB pathways in CD14^+^ cells is central for vaccine efficacy

**Fig. 4:**
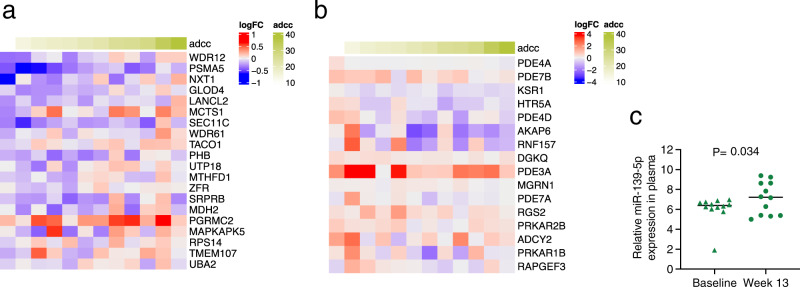
*CREB1* pathway activation and ADCC. **a** Heatmap representations of the log_2_ fold-change (logFC) expression (week 13/baseline) in CD14^+^ cells (*n* = 12 animals) of 20 genes involved in the cellular response to cAMP identified by Tomalka et al.^40^ and their correlation with V2-specific ADCC. **b** Heatmap representations of the log_2_ fold-change expression (week 13/baseline) in CD14^+^ cells (*n* = 12 animals) of 16 genes involved in Gene Ontology Biological Process (GOBP) related to the cAMP-mediated signaling and their correlation with V2-specific ADCC. In **a**, **b**, the V2-specific ADCC response is reported on the top row. **c** Relative expression of miR-139-5p in plasma collected at baseline and week 13 in *n* = 12 animal (two-tailed Wilcoxon signed rank). Displayed *p* values are unadjusted. Source data are provided in the Source Data file and at GEO Series accession number GSE189032.

To validate the CREB pathway activation role at the transcriptional level we conducted GSEA analyses on the transcriptome of CD14^+^ cells isolated at baseline and 1 week following the last immunization. Analysis identified four cAMP pathways that were positively or negatively associated with risk of SIV_mac251_ acquisition (Supplementary Table 10a). Following vaccination (week 13), the expression of genes involved in cellular responses to cAMP were associated with decreased risk of acquisition (unadjusted *p* = 0.096; Supplementary Fig. S6d and Supplementary Table S10a), whereas three cAMP pathways significantly influenced V2-ADCC (Supplementary Table S10b). Interestingly, the fold-change between post-vaccination and baseline of the expression of CREB pathway-genes previously identified as protective in the RV144 trial by Tomalka et al.^40^ correlated positively with V2-ADCC (adjusted *p* = 0.019; Fig. 4a), and genes related to cAMP-mediated signaling correlated negatively with V2-ADCC (adjusted *p* = 0.016; Fig. 4b).

In addition, since in Study 1 we identified the miR-139-5p as a correlate of reduced risk of SIV acquisition due to its possible effect on the *PDE4D*/CREB1 axis^30^, in Study 2, we investigated the miR-139-5p modulatory role on CREB pathway activation in CD14^+^ cells.

In Study 1, a comparative analysis of miR-139-5p levels in the EVs and plasma identified a high correlation of the miR contents between the two compartments (*ρ* = 0.88, *p* = 0.0003; Supplementary Fig. S6e); therefore, in Study 2 we explored the relative expression of miR-139-5p in plasma collected at baseline and following vaccination (week 13). As in Study 1, in Study 2 the miR-139-5p relative expression was increased by vaccination (*p* = 0.034; Fig. 4c). In addition, its vaccine-induced changes (fold-change between week 13 and baseline) positively correlated with the expression of genes involved in CREB pathway activation (adjusted *p* = 0.014 and 0.131; Supplementary Table S10c). These data together confirmed the ability of vaccination to modify the expression of genes involved in the CREB pathway through the modulation of systemic miR-139-5p levels.

### Critical role of efferocytosis and DC-10 in vaccine efficacy

**Fig. 5:**
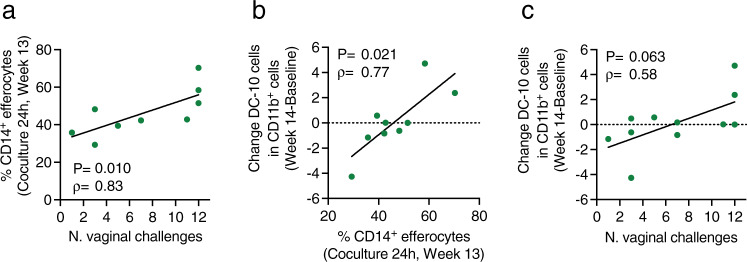
Efferocytosis and DC-10 in Study 2. **a** Correlation between the frequency of CD14^+^ efferocytes cocultured for 24 h and Time of Acquisition (TOA) in *n* = 9 vaccinated animals (week 13). **b**, **c** Correlation between the change from baseline to week 14 in the frequency of DC-10 cells and the frequency of CD14^+^ efferocytes cocultured for (**b**) 24 h in *n* = 9 vaccinated animals (week 13) or (**c**) the TOA in *n* = 11 vaccinated animals. Gating strategy: high SSC/single cells/Live/CD45^+^/Lin^−^(CD3^−^CD20^−^)/HLA-DR^+^/ CD1c^−^/CD11b^+^/CD11c^+^/CD14^+^CD16^+^/CD163^+^/CD141^+^/CD1a^−^. The frequency of DC-10 cells was identified as the frequency of CD1a^−^ cells in CD11b^+^ cells. Correlation analyses: **a**–**c** Two-tailed Spearman correlation test and simple linear regression. Displayed *p* values are unadjusted. Source data are provided in the Source Data file.

The finding that cAMP pathway activation is important in vaccine efficacy raised the hypothesis that pro-resolving professional phagocytes may be an effector mechanism of vaccine efficacy via efferocytosis, a cAMP-dependent process evolved to maintain tissue homeostasis^41,42^. Efferocytosis is induced by the production of IL-13 by T-cells, including Type 1 regulatory T-cells, which induces metabolic reprogramming of monocytes and IL-10 production, in turn activating VAV1/Rac1 via an IL-10 autocrine mechanism to promote apoptotic cell engulfment^43,44^. We hypothesized that since the efficacy of the ALVAC-based vaccine regimen reproducibly correlates with V2-ADCC, an Fc-mediated cytotoxicity process that induces apoptosis of virus expressing cells^45^, the reprogramming of monocytes by vaccination may affect efferocytosis, favoring efficient elimination of apoptotic cells and preventing inflammation. To test this hypothesis, we purified cryopreserved CD14^+^ before and after vaccination from the vaccinated animals in Study 2 and performed efferocytosis assays (Supplementary Fig. S7a, b) using ex vivo macaque neutrophils treated with Staurosporine to induce apoptosis. We found that the frequency of CD14^+^ cells able to engulf apoptotic neutrophils at 24 h of incubation correlated with reduced risk of SIV acquisition when isolated at 1 week following the last immunization (*ρ* = 0.83, *p* = 0.010; Fig. 5a), but not prior to vaccination (*ρ* = 0.23, *p* = 0.473; Supplementary Fig. S7c).

These data prompted us to investigate anti-inflammatory cells as possible sources of IL-10 which may complement efferocytes. Since we observed that CD14^+^CD16^+^ cells correlated with vaccine efficacy in Study 1, we characterized this population in detail using markers that also define tolerogenic dendritic cells able to produce IL-10^11,46^ (Supplementary Fig. S7g). We found that vaccine-induced change in the frequency of CD11c^+^CD14^+^CD16^+^CD163^+^CD141^+^CD1a^−^ cells in CD11b^+^ cells (DC-10) correlated with efferocytosis (*ρ* = 0.77, *p* = 0.021; Fig. 5b), and with delayed SIV_mac251_ acquisition (*ρ* = 0.58, *p* = 0.063; Fig. 5c). Collectively our data suggest that efferocytosis and DC-10 together may create an antiphlogistic environment unfavorable to viral replication, contributing to vaccine efficacy by inhibiting CD4^+^ T-cell activation^11,41,46^.

## Discussion

We demonstrate here that the efficacy of SIV-based DNA/ALVAC/gp120/alum vaccines is age-dependent and confirm that anti-V2 antibodies, particularly those recognizing the V2 in α-helix conformation, which dampen T-cell activation^47^ and mediate ADCC by inducing apoptosis^5^ of infected cells, are critical for vaccine efficacy. In ADCC, the interaction of the antibody Fc region to FcγRIIIa (CD16a) on the surface of natural killer cells induces the release of pro-apoptotic granzyme B and perforin via calcium-dependent pathways and mitogen-activated protein^45^. Virus-infected cells permeabilized by perforin become apoptotic following granzyme B-incorporation^48,49^. The current work demonstrates that efferocytosis is an effector mechanism of vaccine efficacy, and likely essential to complement anti-viral ADCC. Efferocytosis is orchestrated by the pro-resolving cytokine IL-10 and is constituted by highly regulated steps that result in engulfment and digestion of apoptotic cells by efferocytes (a process overlapping with, but distinct from phagocytosis) to recycle cellular components^41^. Defective clearance of apoptotic cells by monocytes results in secondary necrosis of apoptotic cells, accumulation of inflammatory cell debris, overt inflammation^41^, and CD4^+^ T-cell activation.

The induction of efferocytosis by the DNA/ALVAC/gp120/alum regimen likely stems from the ability of most of the vaccine components used here to elicit IL-10. ALVAC increases IL-10 production by PBMCs^50^ and neutrophils^50,51^, alum by infiltrating eosinophils^52,53^, and gp120 by PBMCs^54,55^ and monocytes^56^. IL-10 polarizes monocytes to an anti-inflammatory pro-resolution profile consistent with the vaccine-induced engagement of the immunoregulatory CCL2/CCR2 axis^20^ observed in our studies. CCR2 regulates monocyte mobilization, extravasation, adhesion, and transmigration to inflamed tissue mediated by CCL2. In turn, CCL2 expressed in large quantities by classical monocytes promotes IL-10 production and polarization of monocytes to tolerogenic pro-resolving phenotypes^15^. Importantly, the CCL2/CCR2 axis favors vaccine-induced mucosal recruitment of NKp44^+^ cells, correlating with increased vaccine efficacy^2,57^, as well as recruitment of α_4_β_7_^+^ Th2 cells that do not express CCR5, as previously demonstrated^3^.

Vaccination resulted in chromatin accessibility in CD14^+^ cell genes regulating myeloid cell differentiation, with CREB1-responsive genes, *ZC3H7A*^23^ regulating miRNA biogenesis/processing^25^, and the engagement of innate and adaptive responses via the CCL2/CCR2 axis all being central to vaccine efficacy^30^. Effective efferocytosis of apoptotic cells (killed by V2-specific ADCC and/or by viral infection^58^) appeared to be critical for vaccine efficacy, likely to avoid T-cell activation and viral seeding (Supplementary Fig. S8). The DNA/ALVAC/gp120/alum vaccine regimen was less effective in old than young macaques when compared to their aged-matched controls, likely because of the low-grade systemic chronic inflammation that ensues with aging (inflamm-aging)^59^ and IFN-γ inhibiting engagement of the CCL2/CCR2 axis^42^. Finally, efferocytosis is well-documented to decrease with aging^60,61^.

Our data provide a rational explanation for seemingly contrasting data obtained in humans with ALVAC-based vaccines. Whereas ALVAC-HIV in combination with gp120 formulated in alum reduced the risk of HIV acquisition by 31.2% in the RV144 vaccine trial^1^, no efficacy was observed using gp120 formulated with MF59 in the HVTN-702 trial in South Africa^7^. While differing population risks of HIV acquisition may have contributed to the different outcomes^7^, the current work and prior controlled pre-clinical studies in macaques comparing alum and MF59 with identical SIV-based ALVAC/gp120 vaccines^2^ suggest a role for the MF59 pro-inflammatory profile in the lack of protection observed in HVTN-702. Alum preferentially engages monocytes and macrophages whereas MF59 engages neutrophils that persist in tissues^2^ and whose frequency has been associated with decreased vaccine efficacy^51^. MF59 induces higher levels of interferon type II responses and IL-8 than alum^62^ and does not efficiently activate the cAMP pathway in humans or macaques^40^.

Other vaccine components likely matter as well, as suggested by the lack of efficacy of the more recent HVTN-705 vaccine trial of Southern African women using Ad26-based HIV in combination with gp140/alum boosts^8^. To this point, macaque studies comparing DNA and Ad26 primes followed by ALVAC/gp120/alum boosts have clearly demonstrated a lesser ability of the Ad26 prime to engage CD14^+^ cells, resulting in no vaccine efficacy and higher and persistent pro-inflammatory profiles despite subsequent ALVAC/gp120/alum boosts^3^. Thus, our data suggest that the choice of the adjuvant (MF59) or vector (Ad26) may have primarily contributed to the failures of HVTN-702 and HVTN-705. Our data posit that components of HIV vaccine candidates unable to induce neutralizing antibodies must be carefully evaluated for their specific pro- and anti-inflammatory profiles.

### Limitations of the study

There are some limitations in the current study. First, the use of SIV-DNA copies in the vaginal mucosa as a readout for viral burden and the impossibility of conducting a plaque assay do not allow the identification of active virus at the mucosal level. Second, differential PBMCs’ composition, myelopoiesis, as well as possible polymorphonuclear neutrophil contamination may be confounder effects in the in vitro experiment of PBMC stimulation. Third, it is possible that differential cell-cycle stages in cells used in the ATAC-seq analyses may have affected the results. Fourth, the age gap in the animals used in Study 1 (average age gap 4.63 years) was narrow, precluding a definitive assessment of the age dependence of vaccine efficacy. To best address this point, we repeated the study with older animals (average age gap 13.67 years) and confirmed the age-dependence of the efficacy of this vaccine regimen.

## Methods

### Animals, vaccines, and SIV_mac251_ challenge

All animals used in these studies were female Indian rhesus macaques (macaca mulatta) obtained from Alpha Genesis Inc. (Yemasee, SC), Primate Products Inc. (Immokalee, FL), the National Institute of Child Health and Human Development (NICHD, Rockville, MD), PreLabs (Hines, IL), SNBL (Everett, WA), Worldwide Primates Inc. (Miami, FL), New Iberia Research Center (New Iberia, LA), and Covance Inc. (Princeton, NJ). Animals were housed in two different facilities at the National Institutes of Health (Bethesda, MD) and at Bioqual, Inc. (Rockville, MD). All animals were handled in accordance with the standards of the Association for the Assessment and Accreditation of Laboratory Animal Care (AAALAC) in an AAALAC-accredited facility (OLAW, Animal Welfare Assurance A4149-01 for NIH and A3086-01 for Bioqual). All animal care and procedures were carried out under protocols approved by the NCI and/or NIAID Animal Care and Use Committees (ACUC; Protocol numbers: VB-013 and VB-026 at the NIH and P-181 at Bioqual). Animals were closely monitored daily for any signs of illness, and appropriate medical care was provided as needed. Animals were socially housed per the approved ACUC protocol and social compatibility except during the viral challenge phase when they were individually housed. All clinical procedures, including biopsy collection, administration of anesthetics and analgesics, and euthanasia, were carried out under the direction of a laboratory animal veterinarian. Steps were taken to ensure the welfare of the animals and minimize discomfort of all animals used in this study. Animals were fed daily with a fresh diet of primate biscuits, fruit, peanuts, and other food items to maintain body weight or normal growth. Animals were monitored for psychological well-being and provided with physical enrichment including sanitized toys, destructible enrichment (cardboard and other paper products), and audio and visual stimulation.

*Study 1*. Forty juvenile (categorized as young, average age 3.45 years, 0.545 SD) and twenty-eight adult (categorized as old, average age 7.98 years and 2.60 SD) macaques were assigned to four groups based on their major histocompatibility status, age, and weight. One MamuA01^+^ and one MamuB17^+^ animal were included in the vaccinated group of the young cohort, whereas two MamuA01^+^ animals were included in the vaccinated group of the old cohort. Animals in these age ranges were chosen due to their availability and to ensure their overall health during the study, while allowing for a substantive age difference. Thirteen young and seventeen old macaques were immunized twice with DNA-SIV intramuscularly at weeks 0 and 4 as previously described^3^. Each vaccination contained a total of 4 mg of DNA in 1.2 mL PBS. The animals were given the following DNA constructs: 206 S SIV p57gag_mac239_ (1 mg), 209 S MCP3-p39gag_mac239_ (1 mg), 221 S SIV_macM766_ gp160 (2 mg). At weeks 8 and 12 all 30 macaques were boosted with intramuscular inoculations of 10^8^ plaque forming units (PFU) of recombinant ALVAC (vCP2432), expressing SIV_mac251_ *gag-pro* and *gp120TM* (Sanofi Pasteur, Bridgewater, NJ). At week 12, vaccinated macaques also received 200 μg each of SIV_mac251-M766_ and SIV_smE660-CG7V_ gp120-gD proteins formulated in alum Alhydrogel (Invivogen, San Diego, CA), as previously described^3^. The two proteins were administered together intramuscularly in the opposite thigh of the ALVAC injection site. The thirty immunized macaques and 38 naïve control macaques were challenged intravaginally with 11 repeated, low doses of pathogenic SIV_mac251_ once a week. All controls were naïve as prior studies demonstrated no protection afforded by alum or non-recombinant ALVAC^2,63^. The stock of SIV_mac251_ was propagated in macaque cells (QBI#305342b, Quality Biological, Gaithersburg, MD). Challenge was initiated at 5 weeks following the last immunization (week 17) and each animal was intravaginally administered 1 mL of SIV_mac251_ diluted in RPMI 1640 (Gibco, Waltham, MA) to a final concentration of 4000 TCID_50_/mL (evaluated in rhesus 221 cells). Animals that remained uninfected were subsequently exposed to second challenge. Second challenge was performed by intravaginal exposure with 11 repeated, low doses of pathogenic SIV_mac251_ once a week. The second challenge was initiated 28 weeks following the last immunization in the six animals that remained uninfected following the first challenge. The stock of SIV_mac251_ used in the second challenge was the same as previously used, at the same dilution, and prepared and administered by the same operators. In Figs. 1, 2 and Supplementary Fig. 1–4, vaccinated animals are depicted in red and control animals in black; young animals are represented by filled shapes or continuous lines, and old animals by empty shapes or dashed lines unless otherwise specified.

*Study 2*. Twelve juvenile (young, average age 3.91 years, 0.155 SD), female macaques were enrolled in Study 2. The study included one MamuA01^+^ and one MamuB17^+^ animal. All twelve macaques were immunized similarly to animals in Study 1 at weeks 0, 4, 8, and 12. During DNA prime, the DNA construct 221 S SIV_macM766_ gp160 was substituted with the V1-deleted SIV_macM766_ gp160_ΔV1_, whereas during the protein boost the animals were administered 400 μg of V1-deleted SIV_mac251-M766_ gp120_ΔV1_, as done in a previous study^5^. Animals were challenged intravaginally with 11 repeated, low doses of pathogenic SIV_mac251._ The exposure was done following the same procedure and using the same viral stock and at the same concentration as Study 1. In Figs. 3–5, and Supplementary Figs. 5–7, vaccinated animals are depicted in green and control animals in black.

### Viral RNA and DNA and CD4^+^ T cell count

The RNA copies of SIV_mac251_ in plasma were quantified by nucleic acid sequence-based amplification as previously described^3^. Whole blood counts of CD20^+^, CD4^+^, and CD8^+^ T cell counts in whole blood were assessed by flow cytometry using a previously described protocol^3^. The analyses for proviral DNA load were conducted as described previously^64,65^. Briefly, genomic DNAs from tissues were isolated with the DNeasy blood and tissue kit (Qiagen, Valencia, CA) according to the manufacturer’s protocol, except for a modification at the DNA elution step. The SIV gag gene and the macaque albumin gene sequence (used as an internal control) were amplified by real-time TaqMan qPCR technology in a 7500 real-time system (Applied Biosystems, Waltham, MA). The normalized value of the SIV viral DNA load was calculated as SIV-DNA copy number/macaque albumin gene copy number × 2 × 10^6^ and expressed as the number of SIV viral DNA copies per 10^6^ cells.

### Flow cytometry analysis

To identify monocytic myeloid cells in Study 1, cryopreserved PBMCs (5–10 × 10^6^ cells) collected following the ALVAC-SIV + gp120 immunization (week 13) were thawed and stained with Fluorochrome-conjugated mAbs. The following antibodies were used: PE-Cy7 anti-CD3 (clone SP34-2; cat. #563916, 2.0 µl), PE-Cy7 anti-CD20 (clone 2H7; cat. #560735, 1.0 µl), BV786 anti-NHP-CD45 (clone D058-1283; cat. #563861, 3.0 µl), APC anti-CD14 (clone M5E2; cat. #561390, 7.5 µl), FITC anti-CD16 (clone 3G8; cat. #555406, 5.0 µl), BV421 anti-CD192 (CCR2; clone 48607; cat. #564067, 3.0 µl), PE-CF594 anti-CD184 (CXCR4; clone 12G5; cat. #562389, 5.0 µl), all from BD Biosciences (San Jose, CA), and HLA-DR-APC-Cy7 (clone L243; cat. #307618, 5.0 µl) from BioLegend (San Diego, CA). Aqua LIVE/DEAD viability dye (cat. #L34957, 1 µl; Thermo Fisher Scientific, Waltham, MA) was used to exclude dead cells. All myeloid cell populations were gated as CD45^+^Lin^−^ (CD3 & CD20). Monocyte populations were identified as Lin^−^CD45^+^HLA-DR^+^, and differentiated by the expression of CD14 and CD16, as previously published^3^. Classical monocytes were identified as CD14^+^CD16^−^, Intermediate as CD14^+^CD16^+^, and Non-Classical as CD14^–^CD16^+^.

To identify monocytic myeloid cells in Study 2, cryopreserved PBMCs (5-10 × 10^6^ cells) collected at baseline and following the ALVAC-SIV + gp120_ΔV1_ immunization (week 13) were thawed and stained with Fluorochrome-conjugated mAbs. The following antibodies were used: FITC anti-CD103 (clone B-Ly7; cat. #11-1038-42, 5.0 µl), PerCP-eFluor710 anti-CCR7 (clone 3D12; cat. #46-1979-42, 5.0 µl) from Thermo Fisher Scientific; PE anti-CD33 (clone AC104.3E3; cat. #130-113-349, 2.0 µl) from Miltenyi Biotec (Cambridge, MA); PE-CF594 anti-CXCR4 (clone 12G5; cat. #562389, 5.0 µl), PE-Cy7 anti-CD3 (clone SP34-2; cat. #557749, 1.0 µl), PE-Cy7 anti-CD20 (clone 2H7; cat. #560735, 1.0 µl), APC anti-CCR2 (clone 48607; cat. #558406, 2.0 µl), APC-R700 anti-CD11c (clone 3.9; cat. #566610, 2.5 µl), APC-Cy7 anti-HLA-DR (clone L243; cat. #335796, 2.5 µl), BV421 anti-CD163 (clone GHI/61; cat. #562643, 2.5 µl), BV510 anti-CD16 (clone 3G8; cat. #563830, 2.5 µl), BV650 anti-CD141 (clone 1A4; cat. #740604, 5.0 µl), BV750 anti-PD-L1 (clone MIH1; cat. #746965, 5.0 µl), BV786 anti-CD45 (clone D058-1283; cat. #563861, 2.5 µl), BUV395 anti-CD73 (clone AD2; cat. #742636, 5.0 µl), BUV496 anti-CD1a (clone SK9; cat. #750320, 2.5 µl), BUV563 anti-CD80 (clone 2D10.4; cat. #751730, 2.5 µl), BUV661 anti-CD86 (clone 2331; cat. #741629, 5.0 µl), BUV737 anti-CX3CR1 (clone 2A9-1; cat. #749355, 2.5 µl), BUV805 anti-CD14 (clone M5E2; cat. #612902, 3.0 µl), all from BD Biosciences; PE-Cy5 anti-CD11b (clone ICRF44; cat. #301308, 1.0 µl), PE-Cy5 anti-CD1c (clone L161; cat. #331538, 2.5 µl), all from BioLegend; and Blue LIVE/DEAD viability dye (cat. #L34962, 1 µl; Thermo Fisher Scientific).

#### Antibody cross-reactivity with rhesus macaques

For clones SP34-2, 2H7, D058-1283, M5E2, and 3.9, cross-reactivity is reported on the BD Biosciences website; for clones 3G8, 48607, 12G5, B-Ly7, 3D12, AC104.3E3, GHI/61, 1A4, MIH1, AD2, SK9, 2331 (FUN-1) and 2D10.4, cross-reactivity is reported on the Nonhuman Primate Reagent Resource website (https://www.nhpreagents.org/); for clones L243, 2A9-1, ICRF44, and L161, cross-reactivity is reported on BioLegend website.

Flow cytometry acquisitions for Study 1 were performed on an LSRII and examined using FACSDiva software (BD Biosciences) by acquiring a minimum of 500,000 events for myeloid cell evaluation. Flow cytometry acquisitions for Study 2 were performed on a FACSymphony A5 and examined using FACSDiva software (BD Biosciences) by acquiring all stained cells. Data was further analyzed using FlowJo v10.1 (TreeStar, Inc., Ashland, OR).

Monocyte populations were identified as Lin^−^CD45^+^HLA-DR^+^ and differentiated by the expression of CD14 and CD16, as previously published^3^. Classical monocytes were identified as CD14^+^CD16^−^, Intermediate as CD14^+^CD16^+^, and Non-Classical as CD14^–^CD16^+^. Monocyte subsets were expressed either as frequency of the parental HLA-DR^+^ gate or the live cells. The expression of CCR2 in each monocytic subset was expressed as the frequency of CCR2^+^ cells in their parental subset. In Study 2, DC-10 cells were identified as (high SSC/Singlets/Live CD45^+^/CD3^−^CD20^−^/HLA-DR^+^/ CD1c^−^/CD11b^+^/CD11c^+^/CD14^+^CD16^+^/CD163^+^/CD141^+^/CD1a^−^) and expressed as the frequency of the cells in the final gate (CD1a^−^) of the cells in CD11b^+^ gate.

### CCL2 detection in serum

CCL2 serum levels were measured using a rhesus macaque MCP-1 ELISA commercial kit (Sigma-Aldrich, Saint Louis, MO) following the manufacturer’s instructions as previously described^3^.

### Arginase activity

Arginase activity was assayed on plasma as previously described^66^ using the Arginase Activity Assay Kit (MAK112, Sigma-Aldrich) following the manufacturer’s instructions. Briefly, samples were thawed on ice depleted of the urea by filtration. Samples were assayed in singlicates, whereas controls in duplicates. At the end of the procedure, as specified by the kit, the arginase activity was calculated by the formula that considers the controls as well as the samples’ arginase activity in the presence or absence of the substrate.

### Luminex analysis in plasma

Plasma collected before vaccination, 24 h following the second DNA immunization (week 4), and 1 week following the ALVAC-SIV + gp120 vaccination (week 13) were analyzed using three MILLIPLEX® Non-Human Primate Multiplex assays (EMD Millipore Corporation, Billerica, MD). The samples were assayed following the manufacturer’s instructions. Plasma collected before vaccination and at week 4 + 24 h were assayed for the following targets: GM-CSF, G-CSF, IL-1β, IL-1ra, IL-2, IL-4, IL-5, IL-6, IL-8, IL-10, IL-12/23 (p40), IL-13, IL-17A, IL-18, TNF-α, IFN-γ, MCP-1, MIP-1α, MIP-1β (cat. #PRCYTOMAG-40K-19), Fractalkine, IL-1α, IL-21, IL-22, IL-23, IL-33, IP-10, TNF-β (cat. #PRCYT2MAG-40K-08), and TGF-β1, 2, 3 (cat. #TGFBMAG-64K). Plasma collected at week 13 was assayed for the following targets: IL-1β, IL-2, IL-4, IL-6, IL-8, IL-10, IL-13, IL-17, IFN-γ, MCP-1, MIP-1α (cat. #PRCYTOMAG-40K-11), IL-21, IL-22, IL-23 (cat. #PRCYT2MAG-40K-04), and TGF-β1, 2, 3 (cat. #TGFBMAG-64K). Briefly, samples were thawed on ice, 25 µl of each plasma were loaded in singlet into the plate and mixed with 25 µl of assay buffer and 25 µl of magnetic beads. The plates were incubated at 4 °C for 18 h under agitation at 650 RPM. Following the incubation, the plate was washed, 25 µl of detection antibody were added to each well and incubated for 1 h at room temperature (RT). Next, 25 µl of Streptavidin-PE were added to each well and incubated for 30 min at RT. Finally, the plate was washed and 150 µl of sheath fluid were added to each well. Samples were acquired on a Bio-Plex® 200 System (Bio-Rad, Hercules, CA). Radar plots were generated using Microsoft Excel for Mac (version 16.54).

### In vitro stimulation of healthy young and old rhesus macaque PBMCs

PBMCs from healthy young and old rhesus macaques, frozen in 10% DMSO, were thawed in RPMI 1640 (Corning Life Sciences, New York, NY) supplemented with 10% fetal bovine serum (Access Biologicals, Vista, CA) and 1% penicillin/streptomycin (Gibco). The cells were counted then resuspended at a density of 10^6^ cells per 10 μl. Cells were plated in 96-well plates in a 100 μl total volume, with 80 μl of medium and 10 μl of treatment. The PBMCs were stimulated with 0.5 μg/mL of LPS (Invivogen) and compared to the unstimulated PBMCs control that received 10 μl RPMI instead of the stimulation. Supernatants from all conditions were collected at 24 h post-stimulation and stored at −80 °C.

### Luminex analysis in supernatants of rhesus macaque PBMCs

The MILLIPLEX®MAP Non-Human Primate Cytokine/Chemokine Assay (23-Plex; EMD Millipore Corporation) was used to determine the level of a 23 cytokine/chemokine panel produced by the stimulated PBMCs 24 h after stimulation. The following human chemokine/cytokine premixed panel was used according to the manufacturer’s protocol: GM-CSF, TGF-α, G-CSF, IFN-γ, IL-2, IL-10, IL-15, sCD40L, IL-17, IL-1ra, IL-13, IL-1ß, IL-4, IL-5, IL-6, IL-8, MIP-1α, MCP-1, TNF-α, MIP-1ß, IL-12/23p40, VEGF, IL-18. Briefly, 200 μl of assay buffer were added onto each well of the assay plate and incubated for 10 min then decanted. Subsequently, 25 μl of each standard and control were dispensed in the appropriate wells. Assay buffer was used as blank. Standards were run in duplicates in a 4-fold serial dilution. Twenty-five microliters of assay buffer were then loaded in all sample wells, while 25 μl of medium were dispensed into the standard, control, and blank wells. Samples, thawed in ice, were added to the appropriate wells in a 25 μl volume, followed by 25 μl of the vortexed pre-mixed beads dispensed in all plates. After 2 h incubation at RT, plates were washed and 25 μl of detection antibodies were added into each well and incubated for 1 h, followed by 25 μl of streptavidin-phycoerythrin. Plates were incubated for 30 min before washing. Beads were resuspended in 150 μl of sheath fluid and plates were shaken for 5 min. All incubations were done on a shaker at RT, protected from light. Data was acquired on a Bio-Plex 200 System using bead regions defined in the protocol and analyzed with the Bio-Plex Manager 6.1 software (Bio-Rad). Standard curves were generated, and sample concentrations were calculated in pg/mL. Radar plots were generated using Microsoft Excel for Mac (version 16.54).

### ADCC against SIV_mac251_-infected cells

ADCC activity directed against SIV_mac251_-infected target cells was determined by the ADCC-Luc assay as previously described^67,68^. Briefly, target CEM.NKRCCR5 cells were infected for 48 h with SIV_mac251_ infectious molecular clone virus encoding Renilla luciferase and then incubated with PBMC effector cells (30:1 effector cell/target cell ratio) and plasma dilutions in half-area opaque flat bottom plates (Corning Life Sciences). Incubation was performed in duplicate wells, for 6 h at 37 °C and 5% CO_2_. ADCC activity (percent specific killing) was calculated from the change in relative light units (RLU; Vivi Ren luciferase assay; Promega, Madison, WI) resulting from the loss of intact target cells in wells containing effector cells, target cells, and plasma samples compared to RLU in control wells containing target cells and effector cells alone according to the formula: percent specific killing (number of RLU of target+effector well number of RLU of test well)/number of RLU of target+effector well. Adjusted percentages of specific ADCC killing were determined by subtracting the background activity observed for matched pre-vaccination samples and were reported as reciprocal dilution. After subtraction, negative values were considered as zero. The average of adjusted percentages of specific ADCC killing measured at plasma dilution 1:400, 1:1600, and 1:6400 was calculated and used for the analyses.

### Serum neutralizing antibodies

The levels of Neutralizing antibodies were measured in the serum of vaccinated animals at baseline and week 14 (2 weeks following the last immunization) as a reduction in luciferase reporter gene expression after a single round of infection in TZM-bl cells as described previously. TZM-bl cells were obtained from the NIH AIDS Research and Reference Reagent Program, contributed by John Kappes and Xiaoyun Wu. Test samples were serial-diluted (3-fold dilution in duplicate) and incubated with 200 TCID_50_ of virus in a total volume of 150 μl for 1 h at 37 °C in 96-well flat-bottom culture plates. TZM-bl cells were trypsinized and added to each well (10,000 cells in 100 μl of growth medium containing 20 μg/mL DEAE dextran). A set of wells with cells and virus was used as virus control, and another set of wells with cells only was used as background control. After 48 h incubation, the cells were lysed by the addition of Britelite (PerkinElmer Life Sciences, Waltham, MA), and three quarters of the cell lysate were transferred to a 96-well black solid plate (Corning Costar) for luminescence measurement. Neutralization titers are defined as the dilution at which relative luminescence units were reduced by 50% or 80% compared to that in virus control wells after subtraction of background relative luminescence units. Neutralization was tested against the virus SIV_mac251.6_ (ID #1636DB2), SIV_smE660/BR-CG7G.IR1_ (ID #1370DB2), SIV_smE660/BR-CG7G.IR1_ (ID #1634DB2), and SIV_mac251_ (challenge virus).

### Whole blood Gene-expression analysis

Thirteen young and seventeen old vaccinated macaques were included in a gene-expression analysis. Whole blood (2.5 ml) was collected directly in PAXgene® Blood RNA Tube (cat. #762165; PreAnalytiX, Hombrechtikon, Switzerland) 5 h prior to the first immunization and 24 h following the last immunization in young and old animals. RNA was extracted from PAXgene samples using the PAXgene Blood RNA kit (PreAnalytiX) according to the manufacturer’s instructions. GLOBINclear kit (Ambion, Austin, TX, USA) was used to remove globin transcript. Libraries were loaded on Illumina Truseq Paired-End stranded mRNA Kits and sequenced in 2 × 51 base paired-end runs on an Illumina HiSeq 2500 sequencer with an average of 30 million reads per sample (Illumina, Inc., San Diego, CA). Raw reads were trimmed for low-quality reads and miscalled nucleotides using Trimmomatic v. 0.36. Reads that passed the trimming step were aligned to the rhesus macaque genome (Ensembl Mmul_8.0.1 release 88) using the STAR aligner (version 2.5.3a). Aligned reads were counted using HTSeq (version 0.9.1). Differential expression analysis was performed using the R-package edgeR for count data and limma for fold-changes between post-vaccination and pre-vaccination timepoints. To identify genes differentially expressed after vaccination, a generalized linear model was fitted with the timepoints as the independent variable and the gene counts as the dependent variable. A likelihood ratio test and Benjamini-Hochberg adjustment were used to test statistically for differential expression. An adjusted *p*-value below or equal to 0.05 was used to define differentially regulated genes.

The number of SIV challenges to infection of macaques that were not infected after 11 SIV challenges was recoded as 12. To identify genes regulated by the vaccine and correlated with the number of SIV challenges to infection, a linear regression model was fitted with the number of SIV challenges to infection as an independent variable and the fold-change post/pre of the genes as a dependent variable using the R-package LIMMA^69^. A moderated *t*-test was used to test that the coefficient of regression was statistically different from zero. The Benjamini-Hochberg method was used to correct the *p*-values for multiple testing (adjusted *p*-values). Genes with an adjusted *p*-value below 5% were considered significantly correlated with challenges. Similar analysis was run with edgeR using the number of SIV challenges to infection, the timepoint, the interaction between challenge and timepoint as independent variables, and the gene counts as the dependent variable. A likelihood ratio test and Benjamini-Hochberg adjustment were used to statistically test the significance of the interaction term. The results of analysis using edgeR returned similar genes correlated to challenge as found in LIMMA analysis. The results of analysis using LIMMA are presented in the manuscript.

Gene set enrichment analysis (GSEA) was used to evaluate the gene sets (i.e., pathways) associated with the number of SIV challenges to infection^70^. Genes were pre-ranked by LIMMA t statistic and GSEA was used to assess the enrichment of gene sets from the Molecular Signatures Database gene sets (version 6.1). The GSEA Java desktop program was downloaded from the Broad Institute (http://www.broadinstitute.org/gsea/index.jsp) and used with GSEA Pre-Ranked module parameters (number of permutations: 1000; enrichment statistic: weighted; seed for permutation: 111, 15 ≤ gene set size ≤ 2000).

### Extracellular vesicle isolation from plasma

Blood was collected directly in tubes containing EDTA (K2 EDTA BD Vacutainer, Becton, Dickinson and Company, Franklin Lakes, NJ). After collection plasma was isolated by centrifugation at 900 × *g* for 30 min at 20 °C and then stored at −80 °C. EVs were isolated from plasma as described previously^71^ with minor modifications. This method does not allow for discrimination between apoptotic bodies, microparticles, and exosomes. In addition, since the isolation was performed from whole blood EDTA plasma, the cell-type where EVs originated cannot be identified. Briefly, 1 mL of plasma was thawed in ice, centrifuged at 300 × *g* for 10 min at 4 °C to remove possible remaining cells, then transferred to clean tube and centrifuged at 2000 × *g* for 10 min at 4 °C to remove dead cells. Supernatants were collected and centrifuged at high-speed at 10,000 × *g* for 30 min at 4 °C to remove any remaining cell debris. Following centrifugation, supernatants were transferred to ultracentrifuge tubes (cat. #326814, Beckman Coulter, Brea, CA), diluted with cold sterile phosphate-buffered saline (PBS, pH 7.4) and centrifuged at 25,000 rpm (~46,000 × g) for 2 h at 4 °C. Pellets containing EVs were resuspended in 350 µl of PBS and stored at −80 °C. EV isolation was performed on plasma collected at baseline from only some young animals (*n* = 7) and at week 13 from all young (*n* = 13) and old (*n* = 17) animals of Study 1.

### RNA isolation from EVs and microRNA sequencing

EVs isolated from plasma were processed by Exiqon, Inc. (Woburn, MA). The RNA was isolated from EVs by following a proprietary RNA isolation protocol optimized for serum/plasma (no carrier added). At the end of the isolation the total RNA was eluted in ultra-low volume. The library preparation was done using the QIAseq miRNA Library Kit (cat. #331502, Qiagen). A total of 5 μl of total RNA isolated from EVs was converted into microRNA NGS libraries. Adapters containing UMIs were ligated to the RNA. Then, RNA was converted to cDNA. The cDNA was amplified using PCR (22 cycles) and the indices were added during PCR amplification. After amplification, the samples were purified. Library preparation quality control was performed using either Bioanalyzer 2100 or TapeStation 4200 (Agilent technologies, Santa Clara, CA). The libraries were pooled in equimolar ratios based on quality of the inserts and concentration measurements. The library pools were quantified using Qubit, then sequenced on a NextSeq500 sequencing instrument (Illumina, Inc.) according to the manufacturer’s instructions. An average number of reads of 1 × 10 millions million reads/sample were acquired with a read length of 76 nucleotides and with a single-end read. Raw data was de-multiplexed and FASTQ files for each sample were generated using the bcl2fastq software (version 0.10.1; Illumina, Inc.). FASTQ data were checked using the FastQC tool (http://www.bioinformatics.babraham.ac.uk/projects/fastqc/). Annotation of the obtained sequences was performed using the following reference annotations: Organism Macaca mulatta; Reference genome Mmul 1; Annotation reference: miRbase 20.

Raw counts of miR-Seq were TPM-scaled and quantile normalized to generate the levels of miRs. Following quality control, sequences of samples from 1 young and 1 old vaccinated animal at week 13 were discarded. Differentially expressed genes between young and old animals were determined using Limma-Voom^69^ (Limma package version 3.38.3). Adjusted p-values were generated by the Benjamini-Hochberg method. Downstream analysis and visualization were performed in the NIH Integrated Analysis Portal (NIDAP) using R programs developed on the Palantir Foundry platform (Palantir Technologies, version 5.341.0). To test the effectiveness of the vaccines in preventing SIV infection, the Cox model (R function coxph^72^) was used, considering SIV infection as the outcome, the number of challenges survived as the time variable, and miRNA level for 510 miRNAs as the explanatory variable. For the young and old vaccinated animals, survival was tested as a function of miRNA levels at week 13. Adjusted *p*-values were generated by the False Discovery Rate method. Means, standard deviations (SD), medians, 25th and 75th percentiles, 95% confidence intervals of the medians, and Mann-Whitney test p-values were calculated by Prism 9 for macOS.

### MicroRNA mimic transfection and transcripts quantification

Validation of microRNA effect on proposed targets was performed by transfection of rhesus macaque PBMCs and CD14^+^ cells with mirVana miRNA mimics (Thermo Fisher Scientific) and subsequent relative quantification of RNA transcripts.

#### PBMCs and CD14^+^ cells transfection

PBMCs and CD14^+^ cells isolated from 14 naïve rhesus macaques were transfected by Nucleofection with miR-139-5p mimic (cat. #4464066; assay ID: MC12466; Thermo Fisher Scientific) or a microRNA negative control (mirVana miRNA Mimic, Negative Control #1; cat. #4464058; Thermo Fisher Scientific). Cryopreserved PBMCs were thawed and used directly for electroporation or for isolating CD14^+^ cells by using non-human primate CD14 MicroBeads (Miltenyi Biotec) and following the procedure already described for ATAC-seq and efferocytosis.

A total of 2 × 10^6^ PBMCs and 1.5 × 10^6^ CD14^+^ cells for each microRNA were transfected by using P3 Primary Cell 4D-Nucleofector® X (cat. #V4XP-3024, Lonza, Morristown, NJ, USA) and following manufacturer instructions. Briefly, cells were centrifuged at 900 × *g* for 6 min, the pellet was resuspended in 100 µl of 4DNucleofector Solution, 100 µM of microRNA mimics or negative control were added, and cells were transferred into the cuvettes. Transfection was performed using the 4D-Nucleofector Core Unit instrument (Lonza) with settings EA-100. Following transfection, cells were divided in 2 and either processed directly (0-h incubation) or incubated for 4 h at 37 °C and 5% CO_2_ in incubator (4-h incubation). Following incubation, cells were centrifuged, and dry pellets were stored at −80 °C.

#### RNA isolation and qRT-PCR

RNA isolation was performed using the Quick-RNA microprep kit (cat. #R1051, Zymo Research, Irvine, CA, USA) following manufacturer’s instructions. Briefly, cell pellets were resuspended in 300 µl of RNA lysis buffer and mixed with 300 µl of ethanol. The mix was then transferred into the Spin columns and centrifuged at 11,000 × *g* for 1 min. Next, 400 µl of RNA prep buffer was added to the columns, centrifuged, and washed twice with 700 µl of RNA wash buffer, followed by 400 µl of buffer. The RNA was then eluted in 15 µl of Ultrapure water and stored at −80 °C until used.

The RNA was reverse transcribed using the QuantiTect reverse transcription kit (Qiagen) following manufacturer instructions. Briefly, for each sample, the genomic DNA was eliminated by mixing 1 µg of RNA with gDNA wipeout buffer and incubating at 42 °C for 3 min. The product of the reaction was then mixed with 6 µl of reverse-transcription master mix and incubated at 42 °C for 25 min followed by an incubation at 95 °C for 3 min.

The expression of *PDE4D* was quantified in RNA isolated from cells transfected with miR-139-5p and miR negative control. The expression of the housekeeping gene *GAPDH* was assessed in all samples and used to calculate the relative expression of *PDE4D*. The following primers were used: *GAPDH* (Forward 5′-GCACCACCAACTGCTTAGCAC-3′; Reverse 5′-TCTTCTGGGTGGCAGTGATG-3′) *PDE4D* (Forward 5′-AAAATCACAGGTGGGCTTCA-3′; Reverse 5′-CTGAGGGATTGTGCTCTGGT-3′). Expression was assessed by Power SYBR Green PCR master mix (Applied Biosystems) following manufacturer instructions. Briefly, for each sample, 100 ng of cDNA were mixed with Power SYBR Green PCR master mix containing the primers. Samples were run in duplicates on a Rotor-Gene Q (Qiagen) instrument at 95 °C for 10 min, then cycles of 95 °C for 15 sec and 60 °C for 60 s for a total of 40 cycles. Relative *PDE4D* expression in miR-139-5p mimic transfected samples was calculated as fold-change at 0 and 4-h incubations of the delta delta CT (ΔΔCT) compared to the housekeeping gene and the expression of PDE4D in samples transfected with miR-negative control. First, the following formulas were used to calculate the ΔΔCT at 0-h: [(CT_*PDE4D*_ – CT_*GAPDH*_) for samples transfected with miR-139-5p mimic at time 0 - (CT_*PDE4D*_ – CT_*GAPDH*_) for samples transfected with negative miR at time 0]; and at 4-h [(CT_*PDE4D*_ – CT_*GAPDH*_) for samples transfected with miR-139-5p mimic at time 4h - (CT_*PDE4D*_ – CT_*GAPDH*_) for samples transfected with miR-negative control at time 4h]. Next, the fold changes of *PDE4D* expression in miR-139-5p transfected PBMCs at 0 and 4-h were calculated (formula: 2^−ΔΔCT^) and compared.

### MicroRNA quantification in plasma

#### MicroRNA isolation from plasma

RNA was isolated from plasma collected at baseline and week 13 from 12 animals of Study 2 by using the miRNeasy Serum/Plasma Advanced Kit (cat. #217204, Qiagen) following manufacturer instructions. Briefly, cryopreserved plasma was thawed on ice, and 200 µl of plasma were mixed with 60 µl of buffer RPL and incubated for 3 min at room temperature. Then, 20 µl of buffer RPP were added to the mix and incubated for an additional 3 min at room temperature. Following incubation, the mix was centrifuged at 12,000 × *g* for 3 min and the supernatant was transferred in a new tube, mixed with 1 volume of isopropanol, and transferred to a RNeasy UCP MinElute column. The column was centrifuged at 8000 × *g* for 15 s, washed once with 700 µl of buffer RWT, once with 500 µl of buffer RPE, and once with 500 µl of 80% ethanol. After centrifuging the empty column for 5 min to dry the membrane, the RNA was eluted by adding 20 µl of RNase-free water and centrifuging at 14,000 × *g* for 1 min. Eluted RNA was stored at −80 °C.

#### cDNA synthesis

Following isolation, the RNA was converted into cDNA by using the TaqMan Advanced miRNA cDNA Synthesis Kit (cat. #A28007, Thermo Fisher Scientific) and following manufacturer instructions. Briefly, 2 µl of RNA were mixed with 3 µl of Poly(A) reaction mix and then incubated in thermal cycler at 37 °C for 45 min, 65 °C for 10 min, and then held at 4 °C. Following poly(A) tailing reaction, 10 µl of ligation reaction mix were added to the product of the previous step and incubated in thermal cycler at 16 °C for 60 min and then held at 4 °C. Following the ligation reaction, 15 µl of reverse transcription reaction mix were added to the product of the previous step and incubated in thermal cycler at 42 °C for 15 min, 85 °C for 5 min, and then held at 4 °C. Following the reverse transcription reaction, 5 µl of product were added to 45 µl of miR-Amp reaction mix and incubated in thermal cycler at 95 °C for 5 min, then cycles of 95 °C for 3 s and 60 °C for 30 s for a total of 14 cycles, a final incubation at 99 °C for 10 min, and then held at 4 °C.

#### MicroRNA-139-5p quantification

The relative quantification was performed by TaqMan advanced miRNA assay (cat. #A25576, Thermo Fisher Scientific) and by using the TaqMan fast advanced master mix (cat. #4444963, Thermo Fisher Scientific) and following manufacturer instructions. For mature miR-139-5p the assay ID 478794_mir TaqMan advanced miRNA assay was used, whereas, as suggested by the assay, mir-361-5p was assayed as housekeeping microRNAs by using the assay ID 478056_mir TaqMan advanced miRNA assays. Briefly, following cDNA preparation, 5 µl of cDNA diluted 1:5 were mixed with 10 µl of TaqMan fast advanced master mix and 1 µl of TaqMan advanced miRNA assays. Samples were run in duplicates on a Rotor-Gene Q (Qiagen) instrument at 95 °C for 20 s, then cycles of 95 °C for 3 s and 60 °C for 30 s for a total of 40 cycles. Relative microRNA content was calculated as dCt compared to the housekeeping miR.

### Assay for transposase-accessible chromatin using sequencing (ATAC-seq)

#### Isolation of CD14^+^ cells for ATAC-seq

The isolation of CD14^+^ monocytes in Study 2 was performed from freshly isolated PBMCs (30 × 10^6^ cells) collected at baseline and 1 week following the ALVAC-SIV + gp120_ΔV1_ immunization (week 13) by using non-human primate CD14 MicroBeads (cat. #130-091-097, Miltenyi Biotec) and following manufacturer instructions. Briefly, 30 × 10^6^ Ficoll Plaque isolated PBMCs were incubated with 60 µl microbeads and 240 µl buffer at 4 °C for 15 min. At the end of incubation, cells were washed with 3 ml buffer and resuspended in 500 µl of buffer. Positive selection was performed using the AutoMACSpro (Miltenyi Biotec) using the Possel program (positive selection). At the end of the separation, cells were resuspended in R10 (RPMI media, 10% FBS and 1X antibiotic/antimycotic; GIBCO) and counted for the assay. The purity of CD14^+^ cells was assessed by flow cytometry and using the following antibodies: PE-Cy7 anti-CD20 (clone 2H7; cat. #560735, 2.0 µl), Alexa700 anti-CD3 (clone SP34-2; cat. #557917, 5.0 µl), APC anti-CD14 (clone M5E2; cat. #555399, 7.0 µl), FITC anti-CD16 (clone 3G8; cat. #555406, 5.0 µl) all from BD Biosciences and Violet LIVE/DEAD viability dye (cat. #L34955, 1 µl; Thermo Fisher Scientific). The frequency of CD14^+^CD16^+/−^ cells ranged between 60 and 98% of live cells with a median of 90% (Gating strategy Singlets/Live/CD3^−^CD20^−^/CD14^+^CD16^+/−^). The antibodies’ cross-reactivity with rhesus macaques for clones SP34-2, 2H7, and M5E2 is reported on the BD Biosciences website. Cross-reactivity for clone 3G8 is reported on the Nonhuman Primate Reagent Resource website.

#### Assay for transposase-accessible chromatin on nuclei

ATAC-seq was performed according to Omni-ATAC protocol^73,74^, with a minor modification. Following isolation of CD14^+^ cells, for each sample 50,000 viable cells resuspended in RPMI were briefly transferred in 1.5 ml vials. Cells were pelleted and then washed with 50 µl cold PBS^−^ 1X by centrifugation at 800 × *g* for 5 min at 4 °C. Cell pellets were added to 50 µl cold ATAC resuspension buffer (RSB; 10 mM Tris-HCl pH 7.4, 10 mM NaCl and 3 mM MgCl_2_ in water) containing 0.1% NP40, 0.1% Tween-20, and 0.01% Digitonin, gently resuspended, and incubated for 3 min in ice. Following incubation, 1 mL cold RSB containing 0.1% Tween-20 was added to cells and centrifuged at 800 × *g* for 10 min at 4 °C. Supernatants were carefully removed, and pelleted nuclei were resuspended in 50 µl of transposition mixture containing 5 µl TDE1 Tagment DNA Enzyme (Ref #20034197, Illumina, Inc.) 0.1% Tween-20, 0.01% Digitonin, 10 mM Tris-HCl pH 7.4, 5 mM MgCl_2_, 10% dimethyl formamide in water and PBS^−^ 1X and incubated for 30 min at 37 °C in a thermomixer with 1000 RPM mixing. Following incubation, the transposed DNA was purified by MinElute PCR Purification kit (Ref #28004, Qiagen) following manufacturer instructions, eluted in 11 µl of buffer provided by the kit, and stored at −20 °C.

#### ATAC-seq libraries, sequencing, and data analyses

Libraries for sequencing were generated as previously described^73,74^. Briefly, following a first step of amplification by PCR performed on 10 µl of eluted DNA with 2.5 µM of each primer, 1X NEBNext® high-fidelity PCR master mix and water (5 min at 72 °C; 30 s at 98 °C; 5 cycles of 10 s at 98 °C, 30 s at 63 °C, 1 min at 72 °C), the numbers of additional amplification cycles required to reach the correct amount of DNA was performed by real-time PCR conducted using 5 µl/sample of amplified DNA. Following the assessment, each sample was further amplified with the additional cycles of PCR and size selection was performed using SPRIselect (cat. #B23317, Beckman coulter) to eliminate fragments shorter than 200 bp and longer than 1000 bp. Before sequencing, the DNA quality of the libraries was assessed by regular and high sensitivity D5000 ScreenTape® (Agilent Technologies). The library pools were then sequenced on a HiSeq4000 or NovaSeq S1 sequencing instrument according to the manufacturer’s instructions (Illumina, Inc.). An average number of reads of 50 million/sample were acquired with a read length of 150 or 51 bp and with a paired-end read. Following quality control, samples with Fraction Reads in called peak regions (FRIP) < 5% were removed. Specifically, one sample collected at week 13 from one animal in Study 2 was discarded. Raw sequencing data were demultiplexed by Bcal2fastq v2.17. Adaptor sequence trimming was done by Trimmomatic (v 0.36) or Cutadapt (v 1.18). Bowtie2 (v 2.2.6) was used to map to the rheMac10 (Study 2) reference genome. Duplicate reads were removed using the PICARD tool (v 2.25.7). Peak calling was performed using MACS2 (v 2.2.7.1). Raw counts of mapped reads were obtained using HOMER tools (v 4.11.1) for the called peaks. For downstream analyses, normalized count data by default DESeq2 (v 1.34.0) size factors was used. Any genes with TSS located within ±250 kb distance from the ATAC peak boundary were considered as potential target genes for further analysis. Adjusted *p*-values were generated by the Benjamini-Hochberg method.

### CD14^+^ cells gene expression analysis

The isolation of CD14^+^ cells in Study 2 was performed as described above in the ATAC-seq section. Following isolation, CD14^+^ cells were resuspended in 500 µl RNAlater^TM^ (Invitrogen, Waltham, MA) and stored at −80 °C. RNA isolation was performed using RNeasy Micro Kit (Cat#74004, Qiagen) following manufacturer instructions. Briefly, CD14^+^ cells in RNAlater^TM^ were thawed on ice, 500 µl PBS was added to each vial, and centrifuged at 800 × *g* for 10 min at 4 °C. Supernatants were discarded while cell pellets were processed following manufacturer protocol and resuspended in a final volume of 14 µl of water. The RNA was then used for library preparation and sequencing. Library preparation was done using the NEBNext Ultra II Directional RNA Library Prep for Illumina kit (cat. #E7760L, New England BioLabs) following manufacturer instructions. The final product was quantitated by qPCR before cluster generation and sequencing. Total RNA was sequenced 2x on Novaseq S4 sequencing instrument (Illumina, Inc.) according to the manufacturer’s instructions. An average number of reads of 114 million/sample were acquired with a read length of 151 bp and with a paired-end read. Raw sequencing data were demultiplexed by Bcal2fastq v2.17. Adaptor sequence trimming was done by Cutadapt 1.18. The trimmed RNA-Seq reads were aligned against rheMac10 using STAR 2.7. Raw counts of mapped reads were obtained using the featureCounts program. Normalized data using default DESeq2 size factors were used for further analysis. For analysis of the expression of genes involved in CREB pathways (Supplementary Table S10, Fig. 4a, b, and Supplementary Fig. S6d), raw reads were aligned to the rhesus macaque genome Mmul10 version 103 and counted using the HTSeq function in featureCounts. Differential expression of the CD14^+^ RNA-Seq data (transformed to normally distributed data using the voom function in the R package limma) was done using the Limma framework in R (version 4.1.1). First, genes with low expression (count <10 in all samples, <15 across all samples, or with a count of 0 in more than 30% of samples) were removed (19,754 removed). The remaining count matrix was voom transformed to normally distributed expression. A generalized linear model was fit to the normalized gene expression (dependent variable) with the time to acquisition as an independent variable. A moderated t-test and Benjamini-Hochberg correction were used to assess the significance of the correlation to acquisition, adjusting for multiple testing. Geneset Enrichment analysis, as implemented in the R-package fgsea, was performed on the gene list ranked by t-statistic to assess enrichment of MSigDB genesets (version 7.4). Genesets related to cAMP and *CREB* signaling were identified by looking for the regular expression cAMP in their description. Leading edge genes of the GOBP_CELLULAR_RESPONSE_TO_CAMP genesets were used as *CREB* transcriptomic targets and were subsequently confirmed by mining the literature for publications mentioning the target gene and *CREB* or cAMP in their abstract (GeneRIF) and confirming their relevance.

### Combined gene expression and ATAC-seq analysis in CD14^+^ cells

A strategy to select only accessible enhancer single-sites surrounding vaccine-induced genes in animals with delayed risk of SIV acquisition was applied as described (Supplementary Fig. S5b). The pipeline adopted focuses on pairs of genes and ATAC sites that showed the highest Spearman correlation between accessibility change and TOA for a given gene of interest or higher correlation (absolute [rho]>0.5, arbitrarily chosen threshold) between accessibility change and the RNA expression change (absolute [rho]>0.5). Potential transcription regulators contributing to accessibility were analyzed by de novo motif analysis of the 272 ATAC sites using the HOMER motif analysis^75^.

### V2-specific ADCC killing measured by F(ab’)_2_ blocking

V2-specific ADCC assay was measured using EGFP-CEM-NKr-CCR5-SNAP cells that constitutively express GFP as targets as previously described^5^. Briefly, one million target cells were incubated with 50 μg of ∆V1 gp120 protein for 2 h at 37 °C. The coated target cells were washed and labeled with SNAP-Surface® Alexa Fluor® 647 (New England Biolabs, Ipswich, MA) as recommended by the manufacturer for 30 min at RT. The coated and stained 5000 target cells (50 μl) were incubated for 1 h at 37 °C with 1 μg of purified F(ab’)_2_ fragments from NCI09 monoclonal antibody in 96-well V-bottom plates (MilliporeSigma, Rockville, MD). Plasma samples, heat inactivated at 56 °C for 30 min, were 1:100 diluted and 100 μl were added to the V-bottom plate wells. Human PBMCs (250,000; 50 μl) were added to each well as effectors to give an effector/target (E/T) ratio of 50:1. The plate was incubated at 37 °C for 2 h followed by two PBS washes. The cells were resuspended in 200 μl of a 2% paraformaldehyde/PBS^–^ solution and acquired on an LSRII equipped with a high throughput system (BD Biosciences). Specific killing was measured by loss of GFP from the SNAP-Alexa647^+^ target cells. Target and effector cells cultured in the presence of R10 media were used as background. NCI09 monoclonal antibody (5 μg/ml each, Advanced BioScience Laboratories, Rockville, MD), was used as a positive control. Normalized percent killing was calculated as: (killing in the presence of plasma or plasma + F(ab’)_2_ – background) / (killing in the presence of positive control – background) ×100. Cells incubated with plasma from animals in the absence of F(ab’)_2_ were also parallelly used for determining ADCC killing in each animal. V2-specific ADCC killing was calculated as: ADCC killing in the absence of F(ab’)_2_–ADCC killing in the presence of NCI09 F(ab’)_2_.

### Efferocytosis assay

The frequency of CD14^+^ efferocytes in Study 2 was assessed by Efferocytosis Assay kit (cat. #601770, Cayman Chemical Company, Ann Arbor, MI). CD14^+^ cells were used as effectors, and apoptotic neutrophils as target cells. Ex vivo CD14^+^ monocyte cells rather than differentiated macrophages were used in the assay due to low cell availability.

#### Effector cells

CD14^+^ cells were isolated from cryopreserved PBMCs (10 × 10^6^ cells) collected at baseline and following the ALVAC-SIV + gp120 _ΔV1_ immunization (week 13) by using non-human primate CD14 MicroBeads (#130-091-097, Miltenyi Biotec) and following manufacturer instructions. Briefly, 10 × 10^6^ PBMCs were thawed and incubated with 20 µl microbeads and 80 µl buffer at 4 °C for 15 min. At the end of the incubation cells were washed with 3 ml buffer and resuspended in 500 µl of buffer. Positive selection was performed using the AutoMACSpro (Miltenyi Biotec) following the Possel program.

The purity of CD14^+^ cells was not assessed due to low cell availability. The same isolation method was repeatedly used in previous experiments and the purity was assessed by flow cytometry. The following antibodies were used: PE-Cy7 anti-CD20 (clone 2H7; cat. #560735, 2.0 µl), Alexa700 anti-CD3 (clone SP34-2; cat. #557917, 5.0 µl), APC anti-CD14 (clone M5E2; cat. #555399, 7.0 µl), FITC anti-CD16 (clone 3G8; cat. #555406, 5.0 µl) all from BD Biosciences and Violet LIVE/DEAD viability dye (cat. #L34955, 1 µl; Thermo Fisher Scientific). The purity ranged between 60-98% of live cells (Gating strategy Singlets/Live/CD3^−^CD20^−^/CD14^+^CD16^−^). The antibodies’ cross-reactivity with rhesus macaques for clones SP34-2, 2H7, and M5E2 is reported on the BD Biosciences website. Cross-reactivity for clone 3G8 is reported on the Nonhuman Primate Reagent Resource website.

At the end of the separation, cells were counted and stained with CytoTell^TM^ Blue provided in the kit and following manufacturer instructions. Briefly, cells were resuspended in buffer (10^7^ cells/ml), an equal volume of buffer containing 2X CytoTell^TM^ Blue (stock diluted 1:200 in buffer) was added, incubated at 37 °C for 30 min, and washed three times with R10, resuspend in R10, and used for the efferocytosis assay.

#### Target cells

One unrelated macaque was used as source of neutrophils as target cells. Neutrophils were isolated as previously described^76^. Briefly, following isolation of PBMCs by Ficoll Plaque (GE Healthcare), an equal volume of a solution of 20% dextran in water was added to cellular pellet, gently mixed, and incubated for 1 min, followed by the addition of approximately three volumes of PBS, mixed again, and incubated in the dark for 50–60 min. At the end of incubation, the clear layer at the top of the tube containing neutrophils was collected, washed with PBS, and centrifuged at 900 × *g* for 10 min. Cells were treated with ACK lysing buffer (Quality Biological, Gaithersburg, MD) for 5 min at 37 °C, washed with R10, and counted. Neutrophil purity was not assessed due to low cell availability. The same isolation method was repeatedly used in previous experiments and the purity was assessed by flow cytometry. The following antibodies were used: Alexa700 anti-CD3 (clone SP34-2; cat. #557917, 3.0 µl), Alexa700 anti-CD20 (clone 2H7; cat. #560631, 3.0 µl), Alexa700 anti-CD8 (clone RPA-T8; cat. #561453, 3.0 µl), BV786 anti-CD45 (clone D058-1283; cat. #563861, 3.0 µl) all from BD Biosciences; FITC anti-CD66abce (clone TET2; cat. #130-116-668, 2.0 µl) from Miltenyi Biotec and Blue LIVE/DEAD viability dye (cat. #L34962, 0.5 µl; Thermo Fisher Scientific). The antibodies’ cross-reactivity with rhesus macaques for clones SP34-2, 2H7, D058-1283, RPA-T8, and M5E2 is reported on the BD Biosciences website. Cross-reactivity for clones TET2 and 3G8 is reported on the Nonhuman Primate Reagent Resource website. Neutrophil purity ranged between 75-92% of CD45^+^ cells (Gating strategy Singlets/Live/CD45^+^/CD3^−^CD20^−^CD8^−^/CD66abce^+^).

Neutrophils were stained with CFSE provided by the kit and following manufacturer instructions. Briefly, neutrophils were resuspended in buffer (10^7^ cells/ml), an equal volume of buffer containing 2X CFSE (stock diluted 1:200 in buffer) was added to cells, incubated at 37 °C for 30 min and washed three times with R10. The apoptosis of neutrophils was induced by treatment with Staurosporine Apoptosis inducer provided in the kit. Briefly, isolated cells were resuspended in R10 containing Staurosporine (stock diluted 1:1000) and incubated at 37 °C for 3 h. At the end of incubation, cells were washed two times with R10 and used for the efferocytosis assay.

#### CD14^+^ cells and neutrophils coculture

Effector and apoptotic target cells were cultured alone (as controls) or cocultured at a ratio of one effector CD14^+^ cell to three target apoptotic neutrophils. Cells were incubated in incubator at 37 °C for 12 h or 24 h. In particular, CD14^+^ cells collected at baseline and week 13 isolated from all twelve vaccinated animals were cocultured for 12 h, whereas due to low cell availability, only those isolated from nine animals were cocultured for 24 h. At the end of the coculture, cells were washed with PBS, fixed with 1% paraformaldehyde in PBS, and acquired with a flow cytometer. Flow cytometry acquisitions were performed on a FACSymphony A5 and examined using FACSDiva software (BD Biosciences) by acquiring all stained cells. Data was further analyzed using FlowJo v10.1 (TreeStar, Inc.). The frequency of CD14^+^ efferocytes was determined as the frequency of CFSE^+^ cells (neutrophils) in the CytoTell^TM^ Blue^+^ cells (CD14^+^ cells), therefore representing the frequency of CD14^+^ cells that engulfed the apoptotic neutrophils. Gating strategy: FSC/SSC/Sigle cells/CytoTell^TM^ Blue^+^/CFSE^+^ (Supplementary Fig. S7b).

#### CD14^+^ cell isolation validation method

In order to test the effect of the isolation method on the efferocytic activity, the efferocytosis assay was performed using either CD14^+^ effector cells isolated by positive or negative selection and the results obtained with the two methods were compared. Due to lack of PBMCs isolated from vaccinated macaques, the test was performed using CD14^+^ cells isolated from cryopreserved PBMCs (total of 20 × 10^6^ cells) collected from 12 naïve animals. Following thawing, the PBMCs collected from each animal were mixed and divided in two separate tubes to perform the two isolations starting from identical batches of cells as described below.

#### Positive selection

CD14^+^ cells were isolated as described above by using CD14 microbeads and the AutoMACSpro (Miltenyi Biotec) following the Possel program.

#### Negative selection

CD14^+^ cells were isolated from PBMCs by depletion of CD3^+^, CD20^+^, CD8^+^ and NKG2A^+^ cells. Briefly, 10 × 10^6^ PBMCs were incubated with a mix of PE-conjugated antibodies diluted in buffer for 20 min at room temperature. The following antibodies were used: anti-CD3 (clone SP34-2; cat. #552127, 15 µl), anti-CD8 (clone RPA-T8; cat. #555367, 15 µl), anti-CD20 (clone 2H7; cat. #555623, 15 µl), from BD and anti-CD159a (clone Z199; cat. #IM3291U, 10 µl). At the end of the incubation cells were washed twice with 4 ml of buffer and incubated with 20 µl of PE-microbeads (#130-048-801, Miltenyi Biotec) and 80 µl of buffer for 15 min at 4 °C. At the end of the incubation cells were washed with 3 ml of buffer and resuspended in 500 µl of buffer. Negative separation was performed using the AutoMACSpro following the Deplets program. The antibodies’ cross-reactivity with rhesus macaques for clones SP34-2, 2H7, and RPA-T8 is reported on the BD Biosciences website. Cross-reactivity for clone Z199 is reported on the Nonhuman Primate Reagent Resource website.

#### CD14^+^ cell purity evaluation

Following isolations, the purity of effector cells was checked by flow cytometry. The following antibodies were used: FITC anti-CD16 (clone 3G8; cat. #555406, 2.5 µl), APC anti-CD14 (clone M5E2; cat. #555399, 3.5 µl), Alexa700 anti-CD3 (clone SP34-2; cat. #557917, 2.5 µl), BV650 anti-CD20 (clone 2H7; cat. #563780, 2.5 µl), all from BD Biosciences, and PE-Cy5 anti-HLA-DR (clone L243; cat. #307608, 2.5 µl), BV786 anti-CD8 (clone RPA-T8; cat. #301046, 2.5 µl) from BioLegend; and Blue LIVE/DEAD viability dye (cat. #L34962, 1 µl; Thermo Fisher Scientific). The antibodies’ cross-reactivity with Rhesus macaques for clones SP34-2, 2H7, RPA-T8, and M5E2 is reported on BD Biosciences websites; for clone 3G8 is reported on Nonhuman Primate Reagent Resource website; and for clone L243 is reported on BioLegend website.

Cells were acquired on a FACSymphony A5 and examined using FACSDiva software (BD Biosciences) by acquiring at least 100,000 CD3^−^CD20^−^ cells. Data was further analyzed using FlowJo v10.1 (TreeStar, Inc.). CD14^+^ cells were identified as Lin^–^(CD3^−^CD20^−^CD8^−^)HLA-DR^+^CD14^+^ and expressed as frequency of live cells. Gating strategy: Singlets/Live/CD3^−^CD20^−^/CD8^−^HLA-DR^+^/CD16^+/−^CD14^+^. The negative selection method resulted in a less efficient purification of effector cells. The frequency of CD14^+^ cells in live cells ranged 71.2–80.2% (average 75.5%) following positive selection and 30.1–47.4% (average 36.4%) following negative selection. Comparison showed a difference between the two types of isolation (*p* = 0.063, Wilcoxon *t*-test, *n* = 5 vs 5, Supplementary Fig. S7d).

#### Efferocytosis assay

Following isolation, the efferocytosis assay was conducted as described above and by coculturing effector and target cells for 24 h. Although negatively selected CD14^+^ effector cells showed higher efferocytic activity than positively selected cells (*p* < 0.001, Wilcoxon *t*-test, *n* = 5 vs 5; Supplementary Fig. S7e), Spearman correlation analysis of the efferocytic activity measured using positively or negatively selected CD14^+^ effector cells showed a correlation between the two isolation methods (*p* = 0.061, *ρ* = 0.56, Spearman correlation and simple linear regression, *n* = 12; Supplementary Fig. S7f). The data indicate that the type of CD14^+^ cell isolation method affects the efferocytic activity, however, the positive correlation between the separation methods indicates that the effect is proportional in each sample, and therefore the correlation of the frequency of CD14^+^ efferocytes with a delayed risk of SIV acquisition identified in Study 2 would likely not be affected.

### Statistical analysis

The Mann–Whitney–Wilcoxon test was used to compare continuous factors between young and old macaques. All scatter plots report the median, except where stated otherwise. The number of challenges is a numeric variable taking integer values between 1 and 11 with right-censoring of higher values, recorded as >11. Its associations with continuous variables could be tested using the proportional hazards model, but this model is semi-parametric in that the results are dependent on the distribution of the continuous variable. For a fully nonparametric test, we used Spearman’s rank correlation. For graphing, we assigned 12 as the value of the right-censored numbers, but the ranks are the same for any assigned value >11. The exact log-rank Mantel-Cox test of the discrete-time proportional hazards model was used to compare the viral acquisition in animals exposed to SIV_mac251_. For viral acquisition analyses, the hazard ratio (HR) was calculated based on the Cox proportional hazards model. All statistical tests were performed as two-tailed.

### Reporting summary

Further information on research design is available in the Nature Portfolio Reporting Summary linked to this article.

## Supplementary information

Supplementary Information

Reporting Summary

## Data Availability

The sequencing data discussed in this publication have been deposited in NCBI’s Gene Expression Omnibus (http://www.ncbi.nlm.nih.gov/geo) and are accessible through the following GEO Series accession numbers: Whole blood RNA-seq (Study 1): GSE188901; microRNA-seq (Study 1): GSE188575; CD14^+^ ATAC-seq (Study 2): GSE188879; CD14^+^ RNA-seq (Study 2): GSE189032. Source data are provided with this paper in the “Source Data” file.

## Source data

Source Data

## Materials availability

All materials are available on reasonable request.

## Code availability

The source codes for the whole blood RNA-Seq analysis (Study 1) and CD14^+^ cells RNA-seq analysis (Study 2) are available at: https://github.com/sekalylab/vb013. The source codes for the microRNA-Seq analysis (Study 1) are available at: https://github.com/NIDAP-Community/HIV-vaccine-candidate-efficacy. The source codes for the CD14^+^ cells RNA-Seq/ATAC-Seq analysis (Study 2) are available at: https://github.com/CBIIT/BM-Rhesus-NatureComm2023.git.

## References

[1] Rerks-Ngarm S (2009). Vaccination with ALVAC and AIDSVAX to prevent HIV-1 infection in Thailand. N. Engl. J. Med..

[2] Vaccari M (2016). Adjuvant-dependent innate and adaptive immune signatures of risk of SIVmac251 acquisition. Nat. Med..

[3] Vaccari M (2018). HIV vaccine candidate activation of hypoxia and the inflammasome in CD14(+) monocytes is associated with a decreased risk of SIVmac251 acquisition. Nat. Med..

[4] Gorini G (2020). Engagement of monocytes, NK cells, and CD4+ Th1 cells by ALVAC-SIV vaccination results in a decreased risk of SIVmac251 vaginal acquisition. PLoS Pathog..

[5] Silva de Castro I (2021). Anti-V2 antibodies virus vulnerability revealed by envelope V1 deletion in HIV vaccine candidates. iScience.

[6] Haynes BF (2012). Immune-correlates analysis of an HIV-1 vaccine efficacy trial. N. Engl. J. Med..

[7] Gray GE (2021). Vaccine efficacy of ALVAC-HIV and bivalent subtype C gp120-MF59 in adults. N. Engl. J. Med..

[8] Johnson & Johnson and Global Partners Announce Results from Phase 2b Imbokodo HIV Vaccine Clinical Trial in Young Women in Sub-Saharan Africa. (PR Newswire, 2021).

[9] Solano-Galvez SG (2018). Human Dendritic Cells: Ontogeny and Their Subsets in Health and Disease. Med. Sci..

[10] Manicassamy S, Pulendran B (2011). Dendritic cell control of tolerogenic responses. Immunol. Rev..

[11] Comi M (2020). Coexpression of CD163 and CD141 identifies human circulating IL-10-producing dendritic cells (DC-10). Cell Mol. Immunol..

[12] Finch CE, Morgan TE, Longo VD, de Magalhaes JP (2010). Cell resilience in species life spans: a link to inflammation?. Aging Cell.

[13] Koelman L, Pivovarova-Ramich O, Pfeiffer AFH, Grune T, Aleksandrova K (2019). Cytokines for evaluation of chronic inflammatory status in ageing research: reliability and phenotypic characterisation. Immun. Ageing.

[14] van Duin D (2007). Age-associated defect in human TLR-1/2 function. J. Immunol..

[15] Metcalf TU (2017). Human monocyte subsets are transcriptionally and functionally altered in aging in response to pattern recognition receptor agonists. J. Immunol..

[16] Pinti M (2016). Aging of the immune system: Focus on inflammation and vaccination. Eur. J. Immunol..

[17] Sierra-Filardi E (2014). CCL2 shapes macrophage polarization by GM-CSF and M-CSF: identification of CCL2/CCR2-dependent gene expression profile. J. Immunol..

[18] Metcalf TU (2015). Global analyses revealed age-related alterations in innate immune responses after stimulation of pathogen recognition receptors. Aging Cell.

[19] Xu M, Wang Y, Xia R, Wei Y, Wei X (2021). Role of the CCL2-CCR2 signalling axis in cancer: mechanisms and therapeutic targeting. Cell Prolif..

[20] Gschwandtner M, Derler R, Midwood KS (2019). More than just attractive: how CCL2 influences myeloid cell behavior beyond chemotaxis. Front. Immunol..

[21] Mantovani A, Sica A, Locati M (2005). Macrophage polarization comes of age. Immunity.

[22] Fourati S (2016). Pre-vaccination inflammation and B-cell signalling predict age-related hyporesponse to hepatitis B vaccination. Nat. Commun..

[23] Liang J, Song W, Tromp G, Kolattukudy PE, Fu M (2008). Genome-wide survey and expression profiling of CCCH-zinc finger family reveals a functional module in macrophage activation. PLoS ONE.

[24] Techasintana P, Davis JW, Gubin MM, Magee JD, Atasoy U (2015). Transcriptomic-wide discovery of direct and indirect HuR RNA targets in activated CD4+ T cells. PLoS ONE.

[25] Treiber T (2017). A compendium of RNA-binding proteins that regulate microRNA biogenesis. Mol. Cell.

[26] Yanez-Mo M (2015). Biological properties of extracellular vesicles and their physiological functions. J. Extracell. Vesicles.

[27] Papangeli I (2016). MicroRNA 139-5p coordinates APLNR-CXCR4 crosstalk during vascular maturation. Nat. Commun..

[28] Jung H, Mithal DS, Park JE, Miller RJ (2015). Localized CCR2 activation in the bone marrow niche mobilizes monocytes by desensitizing CXCR4. PLoS ONE.

[29] Gorry PR, Ancuta P (2011). Coreceptors and HIV-1 pathogenesis. Curr. HIV/AIDS Rep..

[30] Cao B (2016). Inactivation of oncogenic cAMP-specific phosphodiesterase 4D by miR-139-5p in response to p53 activation. Elife.

[31] Shen K (2012). MiR-139 inhibits invasion and metastasis of colorectal cancer by targeting the type I insulin-like growth factor receptor. Biochem. Pharm..

[32] Botta C (2018). MiR-29b antagonizes the pro-inflammatory tumor-promoting activity of multiple myeloma-educated dendritic cells. Leukemia.

[33] Liu Y (2011). MicroRNA-98 negatively regulates IL-10 production and endotoxin tolerance in macrophages after LPS stimulation. FEBS Lett..

[34] Li L (2018). MiR-98 modulates macrophage polarization and suppresses the effects of tumor-associated macrophages on promoting invasion and epithelial-mesenchymal transition of hepatocellular carcinoma. Cancer Cell Int..

[35] Wang Q, Shu C, Su J, Li X (2015). A crosstalk triggered by hypoxia and maintained by MCP-1/miR-98/IL-6/p38 regulatory loop between human aortic smooth muscle cells and macrophages leads to aortic smooth muscle cells apoptosis via Stat1 activation. Int. J. Clin. Exp. Pathol..

[36] Gordon JR, Ma Y, Churchman L, Gordon SA, Dawicki W (2014). Regulatory dendritic cells for immunotherapy in immunologic diseases. Front. Immunol..

[37] Netea MG (2016). Trained immunity: a program of innate immune memory in health and disease. Science.

[38] Anderson KL (2000). Transcription factor PU.1 is necessary for development of thymic and myeloid progenitor-derived dendritic cells. J. Immunol..

[39] Eklund EA, Kakar R (1999). Recruitment of CREB-binding protein by PU.1, IFN-regulatory factor-1, and the IFN consensus sequence-binding protein is necessary for IFN-gamma-induced p67phox and gp91phox expression. J. Immunol..

[40] Tomalka JA (2021). The transcription factor CREB1 is a mechanistic driver of immunogenicity and reduced HIV-1 acquisition following ALVAC vaccination. Nat. Immunol..

[41] Doran AC, Yurdagul A, Tabas I (2020). Efferocytosis in health and disease. Nat. Rev. Immunol..

[42] Negreiros-Lima GL (2020). Cyclic AMP regulates key features of macrophages via PKA: recruitment, reprogramming and efferocytosis. Cells.

[43] Proto JD (2018). Regulatory T cells promote macrophage efferocytosis during inflammation resolution. Immunity.

[44] Ip WKE, Hoshi N, Shouval DS, Snapper S, Medzhitov R (2017). Anti-inflammatory effect of IL-10 mediated by metabolic reprogramming of macrophages. Science.

[45] Nimmerjahn F, Ravetch JV (2008). Fcgamma receptors as regulators of immune responses. Nat. Rev. Immunol..

[46] Gregori S (2010). Differentiation of type 1 T regulatory cells (Tr1) by tolerogenic DC-10 requires the IL-10-dependent ILT4/HLA-G pathway. Blood.

[47] Goes LR (2020). The V2 loop of HIV gp120 delivers costimulatory signals to CD4(+) T cells through Integrin alpha4beta7 and promotes cellular activation and infection. Proc. Natl Acad. Sci. USA.

[48] Salk HM, Haralambieva IH, Ovsyannikova IG, Goergen KM, Poland GA (2013). Granzyme B ELISPOT assay to measure influenza-specific cellular immunity. J. Immunol. Methods.

[49] Ravetch JV, Bolland S (2001). IgG Fc receptors. Annu. Rev. Immunol..

[50] Teigler JE (2014). The canarypox virus vector ALVAC induces distinct cytokine responses compared to the vaccinia virus-based vectors MVA and NYVAC in rhesus monkeys. J. Virol..

[51] Musich T (2018). Neutrophil vaccination dynamics and their capacity to mediate B cell help in rhesus macaques. J. Immunol..

[52] Jordan MB, Mills DM, Kappler J, Marrack P, Cambier JC (2004). Promotion of B cell immune responses via an alum-induced myeloid cell population. Science.

[53] Wang HB, Weller PF (2008). Pivotal advance: eosinophils mediate early alum adjuvant-elicited B cell priming and IgM production. J. Leukoc. Biol..

[54] Ameglio F (1994). Recombinant gp120 induces IL-10 in resting peripheral blood mononuclear cells; correlation with the induction of other cytokines. Clin. Exp. Immunol..

[55] Borghi P (1995). Induction of interleukin-10 by human immunodeficiency virus type 1 and its gp120 protein in human monocytes/macrophages. J. Virol..

[56] Planes R, Serrero M, Leghmari K, BenMohamed L, Bahraoui E (2018). HIV-1 envelope glycoproteins induce the production of TNF-alpha and IL-10 in human monocytes by activating calcium pathway. Sci. Rep..

[57] Rahman MA (2019). Differential effect of mucosal NKp44(+) innate lymphoid cells and deltagamma cells on simian immunodeficiency virus infection outcome in rhesus macaques. J. Immunol..

[58] Baxter AE (2014). Macrophage infection via selective capture of HIV-1-infected CD4+ T cells. Cell Host Microbe.

[59] Franceschi C (2000). Inflamm-aging. An evolutionary perspective on immunosenescence. Ann. N. Y Acad. Sci..

[60] De Maeyer RPH (2020). Blocking elevated p38 MAPK restores efferocytosis and inflammatory resolution in the elderly. Nat. Immunol..

[61] Arnardottir HH, Dalli J, Colas RA, Shinohara M, Serhan CN (2014). Aging delays resolution of acute inflammation in mice: reprogramming the host response with novel nano-proresolving medicines. J. Immunol..

[62] Seubert A, Monaci E, Pizza M, O’Hagan DT, Wack A (2008). The adjuvants aluminum hydroxide and MF59 induce monocyte and granulocyte chemoattractants and enhance monocyte differentiation toward dendritic cells. J. Immunol..

[63] Pegu P (2013). Antibodies with high avidity to the gp120 envelope protein in protection from simian immunodeficiency virus SIV(mac251) acquisition in an immunization regimen that mimics the RV-144 Thai trial. J. Virol..

[64] Shytaj IL (2015). Two-year follow-up of macaques developing intermittent control of the human immunodeficiency virus homolog simian immunodeficiency virus SIVmac251 in the chronic phase of infection. J. Virol..

[65] Lee M, Kim WK, Kuroda MJ, Pal R, Chung HK (2016). Development of real-time PCR for quantitation of simian immunodeficiency virus 2-LTR circles. J. Med. Primatol..

[66] Vaccari M (2019). Myeloid cell crosstalk regulates the efficacy of the DNA/ALVAC/gp120 HIV vaccine candidate. Front. Immunol..

[67] Pollara J (2011). High-throughput quantitative analysis of HIV-1 and SIV-specific ADCC-mediating antibody responses. Cytom. A.

[68] Pollara J (2019). Bridging vaccine-induced HIV-1 neutralizing and effector antibody responses in rabbit and rhesus macaque animal models. J. Virol..

[69] Ritchie ME (2015). limma powers differential expression analyses for RNA-sequencing and microarray studies. Nucleic Acids Res..

[70] Subramanian A (2005). Gene set enrichment analysis: a knowledge-based approach for interpreting genome-wide expression profiles. Proc. Natl Acad. Sci. USA.

[71] Thery, C., Amigorena, S., Raposo, G. & Clayton, A. Isolation and characterization of exosomes from cell culture supernatants and biological fluids. *Curr. Protoc. Cell Biol.* **Chapter 3**, Unit 3 22 (2006). 10.1002/0471143030.cb0322s30 18228490

[72] Therneau, T. M. *A Package for Survival Analysis in S* https://CRAN.R-project.org/package=survival (2015).

[73] Corces MR (2017). An improved ATAC-seq protocol reduces background and enables interrogation of frozen tissues. Nat. Methods.

[74] Fujiwara S, Baek S, Varticovski L, Kim S, Hager GL (2019). High quality ATAC-seq data recovered from cryopreserved breast cell lines and tissue. Sci. Rep..

[75] Heinz S (2010). Simple combinations of lineage-determining transcription factors prime cis-regulatory elements required for macrophage and B cell identities. Mol. Cell.

[76] Khandpur R (2013). NETs are a source of citrullinated autoantigens and stimulate inflammatory responses in rheumatoid arthritis. Sci. Transl. Med..

