# Supplementary material for: HIV vaccine candidate efficacy in female macaques mediated by cAMP-dependent efferocytosis and V2-specific ADCC
Source: Nat Commun. 2023 Feb 2;14:575. doi: 10.1038/s41467-023-36109-8 (PMC9894672; doi:10.1038/s41467-023-36109-8)
Supplement: Supplementary file 1 — Supplementary Information [file 41467_2023_36109_MOESM1_ESM.pdf]

Supplementary Information

**Journal:** Nature Communications

**Article Title:**	**HIV vaccine candidate efficacy in female macaques mediated by cAMP-dependent efferocytosis and V2-specific ADCC**
**Corresponding Author:**	Genoveffa Franchini, M.D. email: franchig@mail.nih.gov

Supplementary Item & Number	Title
Supplementary Figure S1	Viral load and *in vitro* PBMC stimulation in young and old animals.
Supplementary Figure S2	Monocytes, CCL2, and Specific ADCC Killing.
Supplementary Figure S3	*Ex vivo* and *in vitro* cytokine/chemokine levels in young and old macaques.
Supplementary Figure S4	Gene expression in young and old animals and miR-139-5p effect on *PDE4D*.
Supplementary Figure S5	Chromatin accessibility and vaccination.
Supplementary Figure S6	*CREB1* Chromatin accessibility and risk of SIV acquisition.
Supplementary Figure S7	Efferocytosis assay and time of acquisition.
Supplementary Figure S8	Hypothetical mechanisms of vaccine efficacy.
Supplementary Table S1	Serum neutralizing antibody responses to SIV viruses.
Supplementary Table S2	Cytokine/Chemokine levels in plasma of young and old macaques.
Supplementary Table S3	Cytokine/Chemokine expression in PBMCs of young and old macaques.
Supplementary Table S4	BioAge modules and risk of SIV acquisition.
Supplementary Table S5	Gene expression and risk of SIV acquisition.
Supplementary Table S6	Differently expressed miRNAs in old and young animals.
Supplementary Table S7	MicroRNAs correlating with susceptibility to SIV acquisition in young vaccinated animals.
Supplementary Table S8	MicroRNAs correlating with susceptibility to SIV acquisition in old vaccinated animals.
Supplementary Table S9	Vaccine-induced genes in animals with delayed risk of SIV acquisition.
Supplementary Table S10	Cyclic AMP (cAMP) pathways correlated with risk of SIV_{mac251} acquisition and V2-specific ADCC in $CD14^+$ cells isolated from n=12 animals of Study 2.

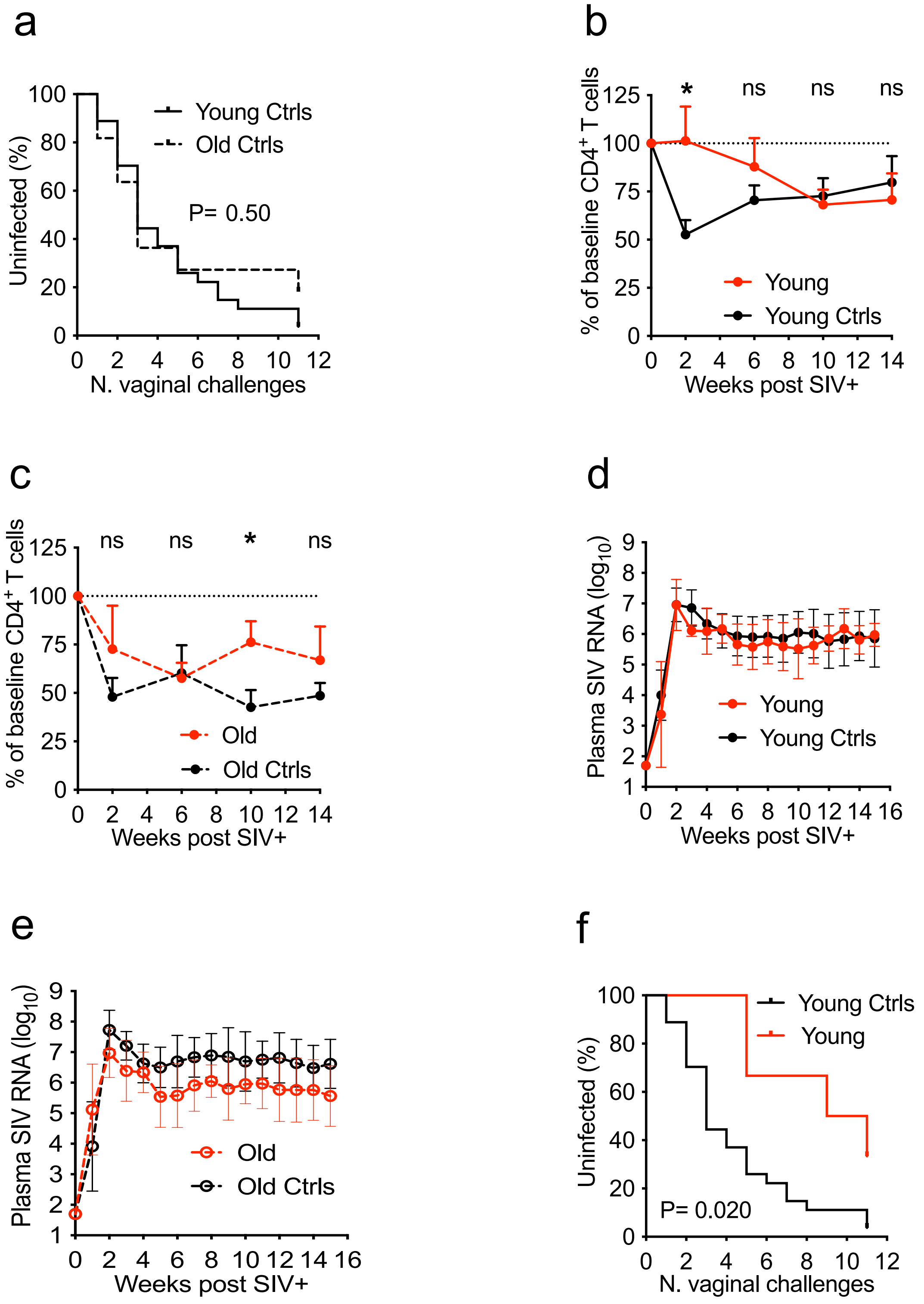

Supplementary Figure 1

Supplementary Figure S1. Viral load and *in vitro* PBMC stimulation in young and old animals. (**a**) SIV_{mac251} acquisition in naïve animals. The number of intravaginal exposures before virus acquisition was assessed in the n=27 young and n=11 old control animals using the Log-rank (Mantel-Cox test). (**b, c**) Percentage of $CD4^+$ T-cell changes in blood over time following SIV_{mac251} infection (weeks; mean ± s.e.m.) in (**b**) n=7 vaccinated and n=18 control young animals (p=0.012 at week 2); and (**c**) n=11 vaccinated and n=9 control old animals (p=0.027 at week 10). The (*) or (ns) at each time-point indicates two-tailed Mann-Whitney test between the two groups $p<0.05$ or $p>0.05$, respectively. (**d, e**) SIV RNA levels in plasma over time following SIV_{mac251} infection (weeks; geometric mean with error) in the (**d**) n=7 vaccinated and n=26 control young animals; and (**e**) n=11 vaccinated and n=9 control old animals. (**f**) Time of SIV_{mac251} acquisition in young animals to second (28 weeks following the last immunization) viral exposure. The number of intravaginal exposures before virus acquisition was assessed in n=6 young vaccinated animals during the second viral exposure compared to n=27 young controls using the Log-rank (Mantel-Cox test). Displayed p values are unadjusted. Source data are provided in the Source Data file.

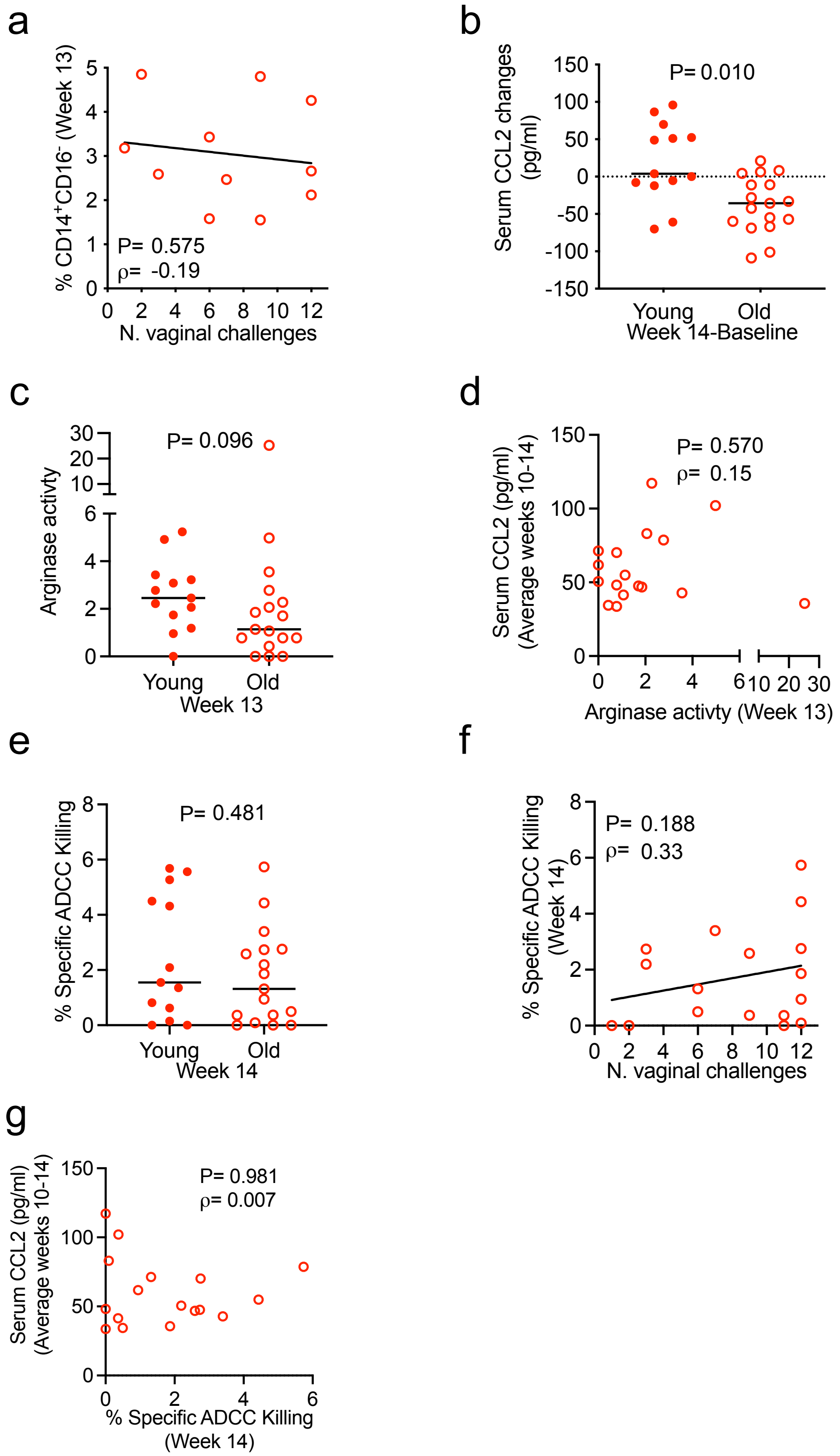

Supplementary Figure 2

Supplementary Figure S2. Monocytes, CCL2, and Specific ADCC Killing. (**a**) Correlation between the frequency of classical monocytes ($CD14^{+}CD16^{-}HLA\text{-}DR^{+}$ in live cells) at week 13 and the time of acquisition (TOA) in the n=11 old vaccinated animals. (**b**) Change from baseline to week 14 of serum levels (pg/ml) of CCL2 in n=13 young and n=17 old vaccinated animals. (**c**) Arginase activity measured in plasma at week 13 in n=13 young and n=17 old vaccinated animals. (**d**) Correlation between the arginase activity measured in plasma at week 13 and the average of the serum CCL2 levels (pg/ml) at weeks 10 and 14 in n=17 old vaccinated animals. (**e**) Average of the percentages of adjusted specific ADCC killing of SIV_{mac251}-infected cells assessed at different plasma dilutions (1:400, 1:1,600, and 1:6,400) at week 14 in n=13 young and n=17 old vaccinated animals. (**f**) Correlations between the average of adjusted specific ADCC killing of SIV_{mac251}-infected cells at week 14 assessed at different plasma dilutions (1:400, 1:1,600, and 1:6,400) and the TOA in n=17 old vaccinated animals. (**g**) Correlation between the average of the serum CCL2 levels (pg/ml) at weeks 10 and 14 and the average of the percentages of adjusted specific ADCC killing of SIV_{mac251}-infected cells assessed at different plasma dilutions (1:400, 1:1,600, and 1:6,400) at week 14 in n=17 old vaccinated animals. Comparisons: (**b, c, e**) two-tailed Mann-Whitney test and the medians. Correlation analyses: (**a, d, f, g**) two-tailed Spearman correlation test and simple linear regression. Displayed p values are unadjusted. Source data are provided in the Source Data file.

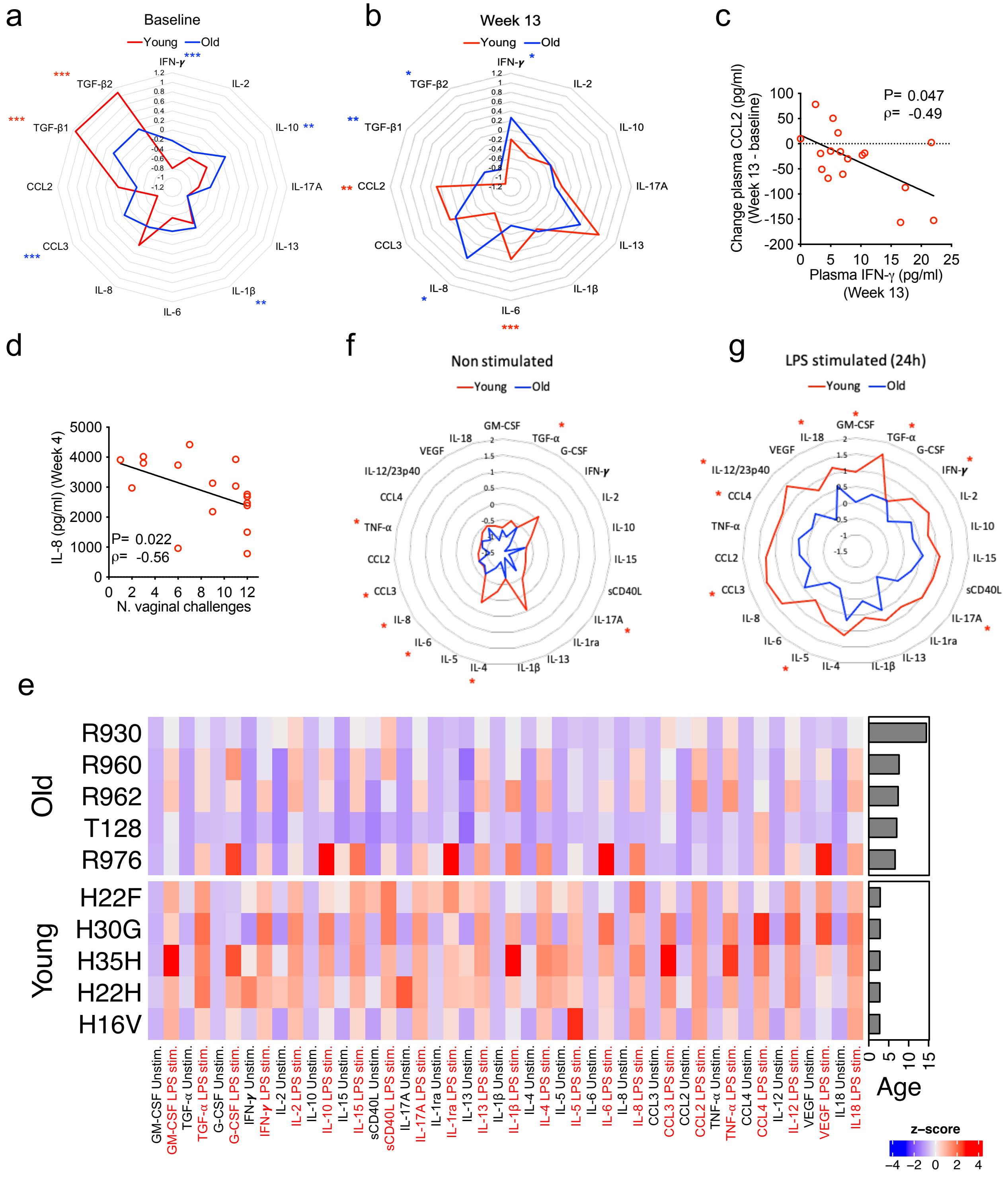

Supplementary Figure 3

Supplementary Figure S3. *Ex vivo* and *in vitro* cytokine/chemokine levels in young and old macaques. (**a**, **b**) Radar plots showing different expressions of cytokines and chemokines between n=13 young (red) and n=17 old (blue) animals at (**a**) baseline and (**b**) week 13. (**c**) Correlation between the variation of the plasma CCL2 levels (pg/ml) at weeks 13 and baseline and the plasma levels at week 13 of IFN-γ in n=17 old animals. (**d**) Correlation between the plasma levels at week 4 (24h following second DNA administration) of IL-8 and the time of acquisition in n=17 old animals. (**e**) Heatmap representation of the 24-hour expression of different cytokines and chemokines in unstimulated (black) or LPS-stimulated (red) PBMCs from n=5 young and n=5 old naïve macaques. Assayed cytokines (in order of appearance): GM-CSF, TGF-α, G-CSF, IFN-γ, IL-2, IL-10, IL-15, CD40L, IL-17, IL-1ra, IL-13, IL-1β, IL-4, IL-5, IL-6, IL-8, CCL3, CCL2, TNF-α, CCL4, IL-12, VEGF, IL-18. The age is shown as bars on the right of the heatmap. Values are presented as z-scores scaled across animals and stimulations. (**f, g**) Radar plots showing different expressions of cytokines and chemokines between n=5 young (red) and n=5 old (blue) animals. (**f**) Non stimulated and (**g**) LPS-stimulated PBMCs. (**a, b, f, g**) In all radar plots, data are plotted as normalized z-scores for each cytokine/chemokine across all the animals. Comparisons were performed using two-tailed Mann-Whitney test on pg/ml measured levels and presented as * $p<0.05$, ** $p<0.01$, *** $p<0.001$ (red when higher in the young group, blue when higher in the old group. P values are reported in Supplementary Tables S2 for a-b, and S3 for f-g). Correlation analyses: (**c-d**) two-tailed Spearman correlation test and simple linear regression line. Displayed p values are unadjusted. Source data are provided in the Source Data file.

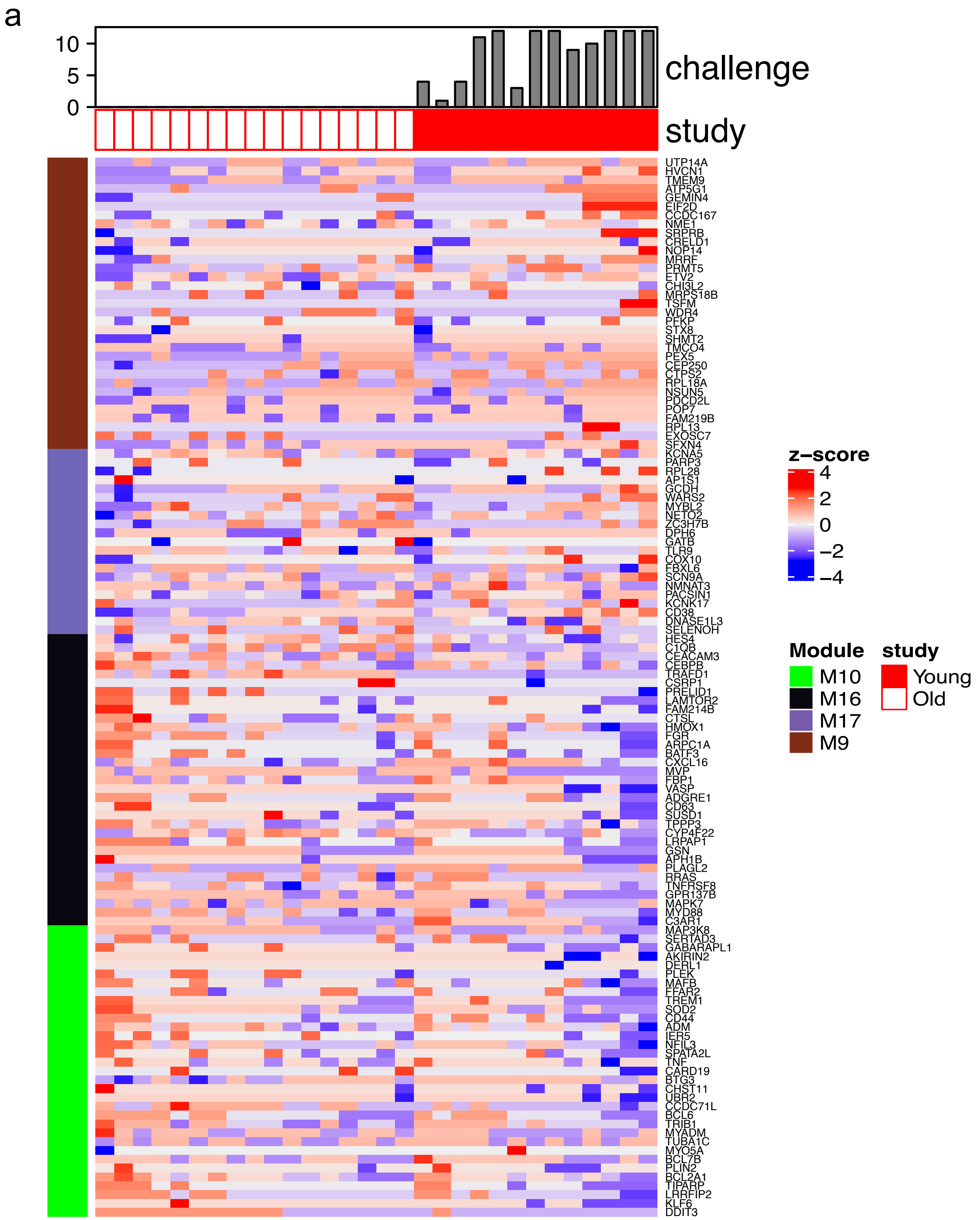

Supplementary Figure 4

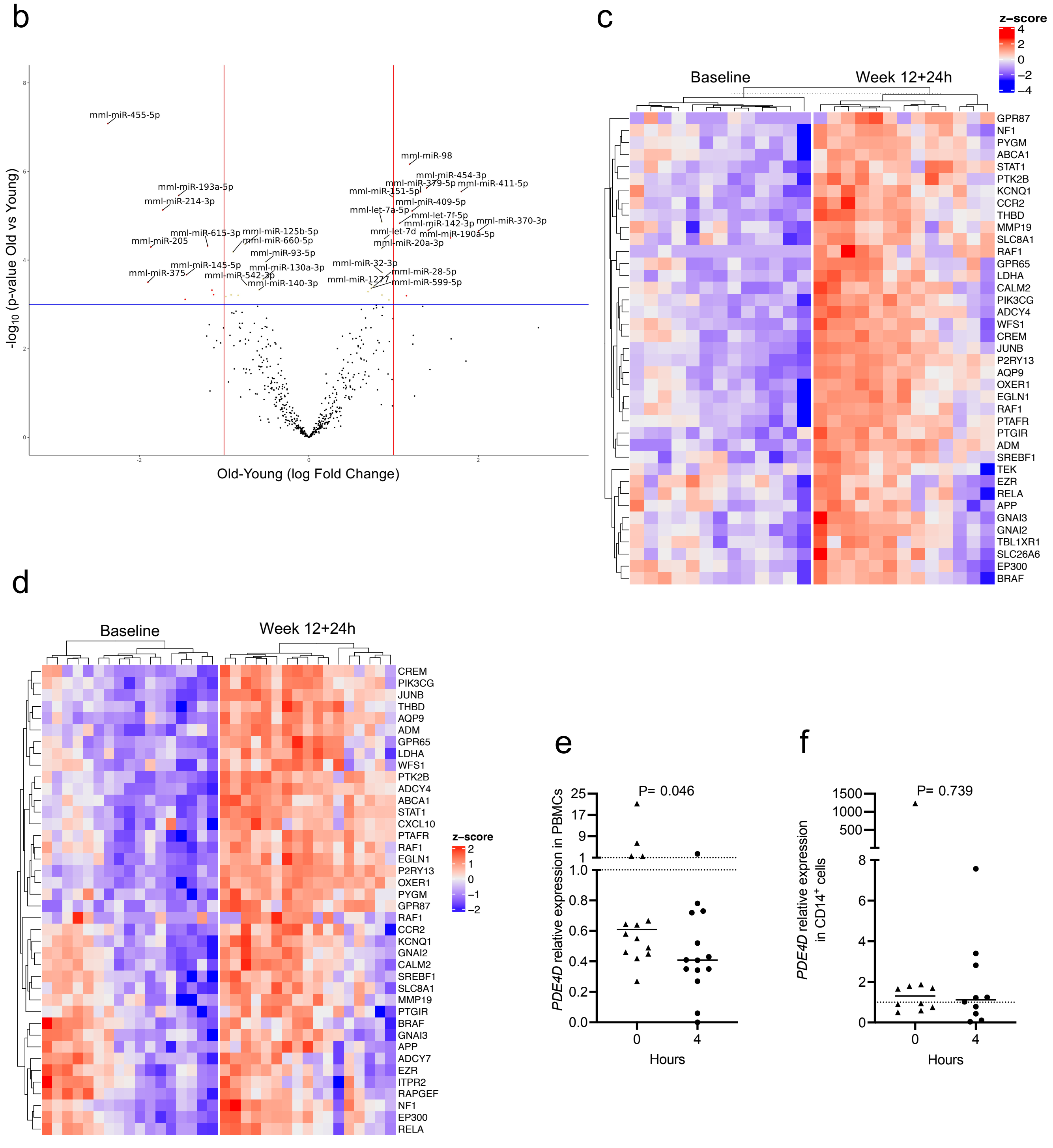

Supplementary Figure 4

Supplementary Figure S4. Gene expression in young and old animals and miR-139-5p effect on *PDE4D*. (**a**) BioAge modules associated with age and risk of SIV_{mac251} acquisition 24h following last immunization (week 12+24h). Heatmap representation of the expression of 120 genes divided in four BioAge modules (M9->33; M10->21; M16->33; M17->33) associated with risk of acquisition in n=13 young animals (adjusted p-values for fold-changes and time of acquisition: M9 p=0.0043; M10 p=0.0301; M16 p=0.0259; M17 p=0.0321). Modules are indicated on the left column. Challenges required to infection in young animals are indicated on the top. (**b**) Volcano plot representation of statistical significance against fold-change of the expression of microRNA in EVs between n=12 young and n=16 old vaccinated animals. MicroRNAs above the blue horizontal line reached higher significance (p<0.001). By comparing the expression in old and young animals, miRNAs located on the left and the right of the two vertical red lines respectively showed higher fold-change in decreased or increased expression. (**c, d**) Heatmap representation of the TMM normalized expression scaled to a mean of 0 and a standard deviation of 1 across all the samples of 39 genes involved in the CREB pathway in n=13 young (**c**) and n=17 old (**d**) animals at baseline and week 12+24h. The list of genes plotted in the heatmap are part of three GO terms (*i.e.* union of leading edges genes): GO Positive regulation of cAMP metabolic process (subterm of GO Regulation of cAMP metabolic process), GO Regulation of cAMP metabolic process (previous work[1-3]), and GO Response to cAMP (Reference for entries can be found at http://amigo.geneontology.org/amigo/term/GO:0051591). (**e, f**) Relative *PDE4D* expression in PBMCs from n=14 animals (**e**) and $CD14^+$ cells from n=10 animals (**f**) at 0 and 4-hours following transfection with miR-139-5p. In **e** and **f**, the relative *PDE4D* expression in miR-139-5p mimic transfected samples was calculated as fold-change relative to expression of *GAPDH* housekeeping gene and to expression of *PDE4D* in miR

negative control transfected samples. Comparison was performed by two-tailed Mann-Whitney test and the medians are shown. In **e** and **f**, displayed p values are unadjusted. Source data are provided in the Source Data file and at GEO Series accession numbers GSE188901 (https://www.ncbi.nlm.nih.gov/geo/query/acc.cgi?acc=GSE188901) and GSE188575 (https://www.ncbi.nlm.nih.gov/geo/query/acc.cgi?acc=GSE188575).

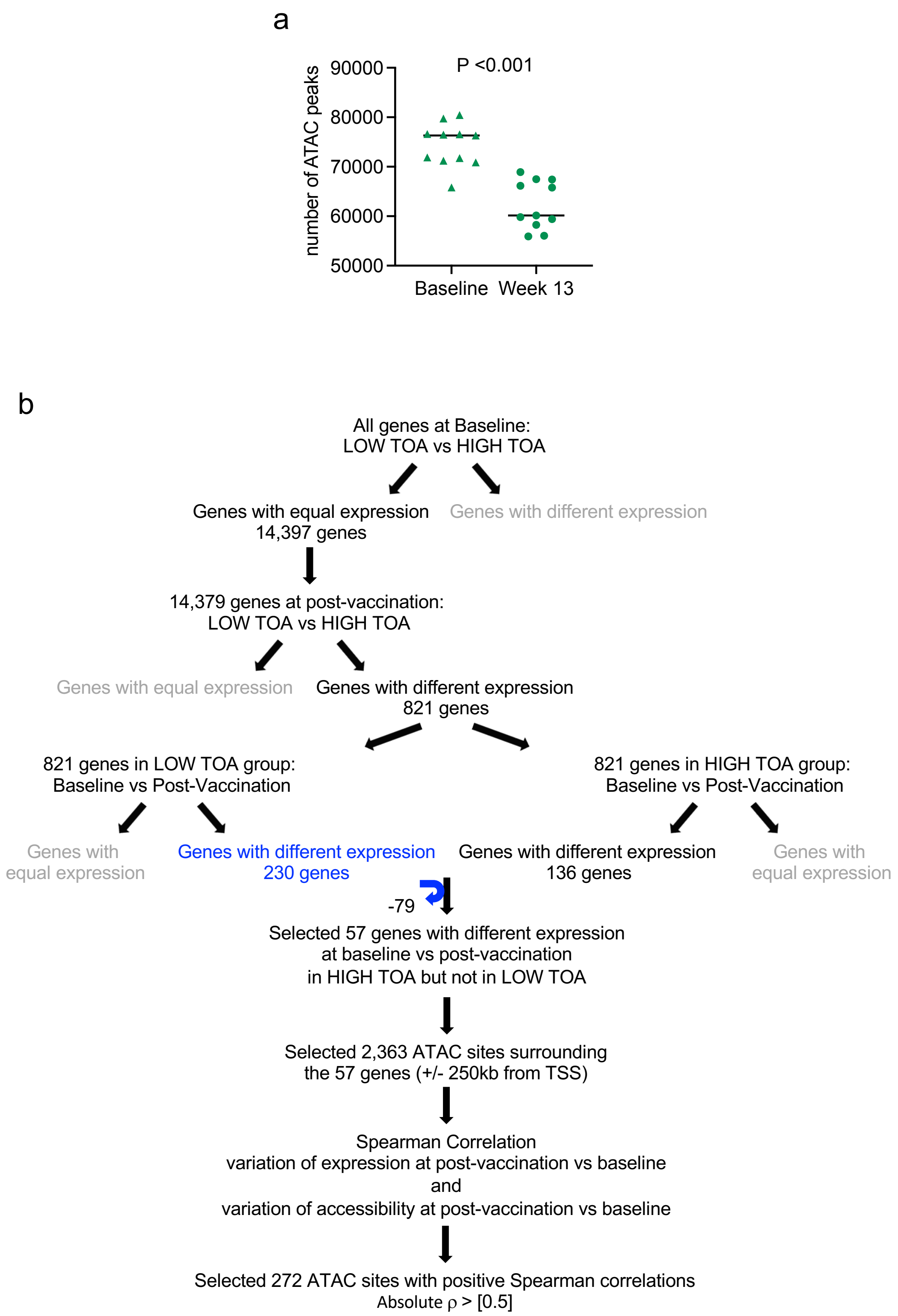

Supplementary Figure 5

Supplementary Figure S5. Chromatin accessibility and vaccination. (**a**) Number of ATAC peaks in n=11 vaccinated animals of Study 2 at baseline and 1 week following last immunization (Week 13; unadjusted p=0.00098). Two-tailed Wilcoxon signed rank test and median. (**b**) Schematic pipeline of the strategy to select accessible enhancer single-regions surrounding vaccine-induced genes in animals with delayed risk of SIV acquisition. First, genes with differential expression in animals that acquired the virus earlier or later than 8 viral exposures (Low and High Time of Acquisition [TOA], respectively) were excluded to select only the genes that were affected by vaccination. Then, gene expression at post-vaccination of the genes with equal expression at baseline were compared in low and high TOA animals. Genes differentially expressed were compared at baseline and post-vaccination both in the low and high TOA groups. From the 136 genes affected by vaccination in the high TOA group, we removed 79 genes that were affected by vaccination in low TOA group as well (in blue), allowing the selection of 57 genes whose expression was modified by vaccination and that are present only in animals with delayed SIV acquisition (TOA>8). Finally, 272 ATAC sites that showed an absolute two-tailed Spearman correlation of at least $\rho>|0.5|$ between the variation at baseline and post-vaccination of the accessibility of ATAC sites surrounding (+/- 250kb from TSS) the 57 genes, and the variation at baseline and post-vaccination of the expression of the same 57 genes, were selected. These 272 sites represent the regions that were modified by vaccination, that were present only in animals with delayed risk of SIV acquisition, and that affected gene expression and therefore were further analyzed by HOMER *de novo* motif analysis. Source data are provided in the Source Data file and at GEO Series accession numbers GSE188879 (https://www.ncbi.nlm.nih.gov/geo/query/acc.cgi?acc=GSE188879) and GSE189032 (https://www.ncbi.nlm.nih.gov/geo/query/acc.cgi?acc=GSE189032).

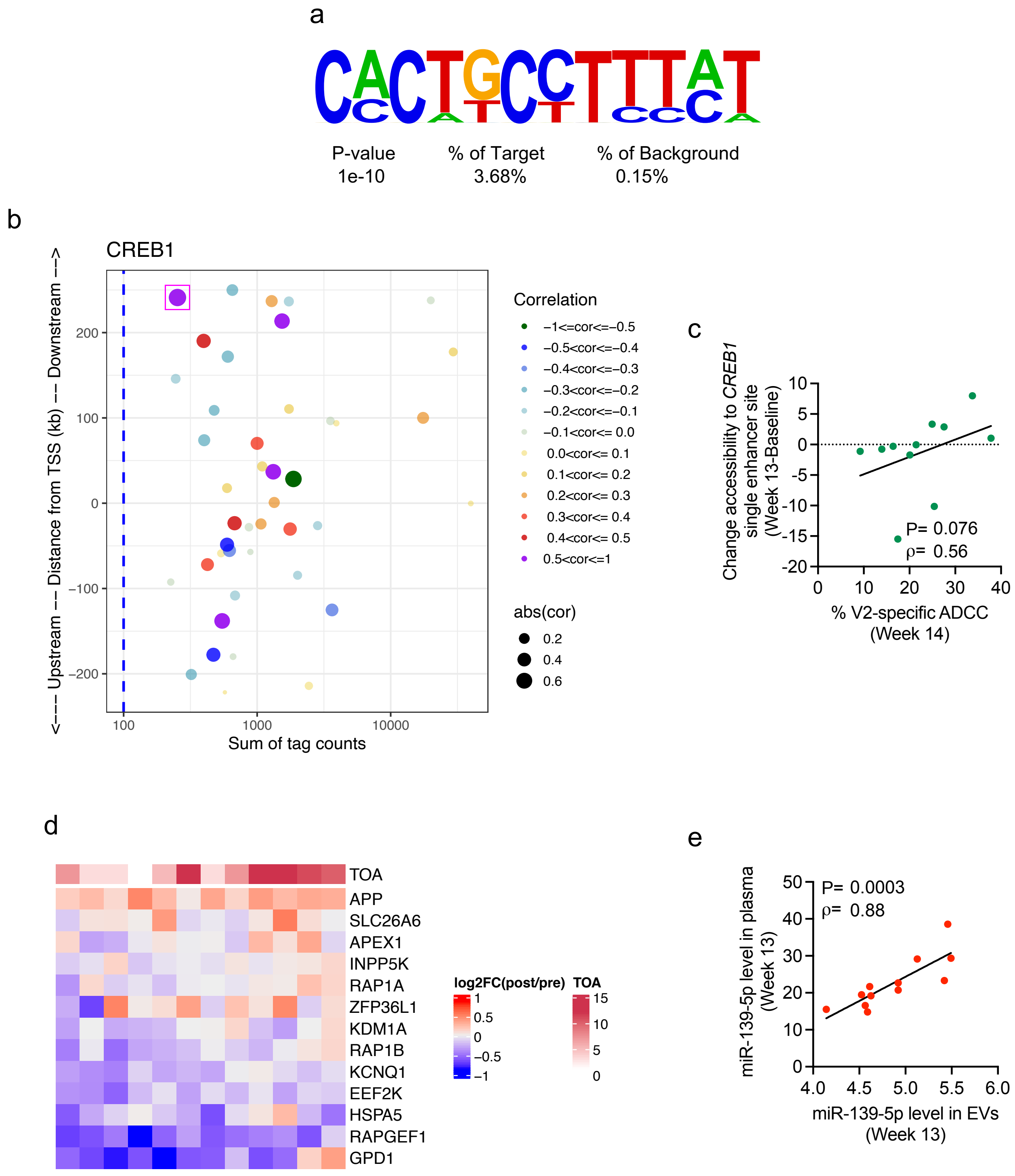

Supplementary Figure 6

Supplementary Figure S6. *CREB1* Chromatin accessibility and risk of SIV acquisition. (**a**) PU.1, SPIB, SPI1 top enriched motif among the 272 ATAC sites identified by the pipeline in (Supplementary Figure 5b). The analysis was done by focusing on the central 100 bp of each ATAC site. The p-value, target percentage, and background percentage are reported. (**b**) ATAC sites around *CREB1* TSS and their two-tailed Pearson correlation between their variation of the intensity of the accessibility at baseline and week 13 with the TOA. The ATAC site with highest correlation is framed by a square. Threshold sum of raw tag counts: 100 (dashed blue line). Size of the dots: absolute R value of correlation with TOA. Dot color: R values. (**c**) Two-tailed Spearman correlation test and simple linear regression line between V2-specific ADCC at week 14 and the variation between baseline and week 13 of the intensity of the accessibility of the single *CREB1* ATAC-site selected in (**b**) in n=11 vaccinated animals. (**d**) Heatmap representation of the log_2 fold-change expression (week 13/baseline) of 13 genes involved in the cellular response to cAMP identified in $CD14^+$ cells of n=12 vaccinated animals. The number of challenges required for acquisition (TOA) are reported on top. (**e**) Two-tailed Spearman correlation test and simple linear regression line between levels of miR-139-5p in extracellular vesicles and plasma at week 13 in n=12 young animals. Displayed p values are unadjusted. Source data are provided in the Source Data file and at GEO Series accession numbers GSE188879 (https://www.ncbi.nlm.nih.gov/geo/query/acc.cgi?acc=GSE188879) and GSE189032 (https://www.ncbi.nlm.nih.gov/geo/query/acc.cgi?acc=GSE189032).

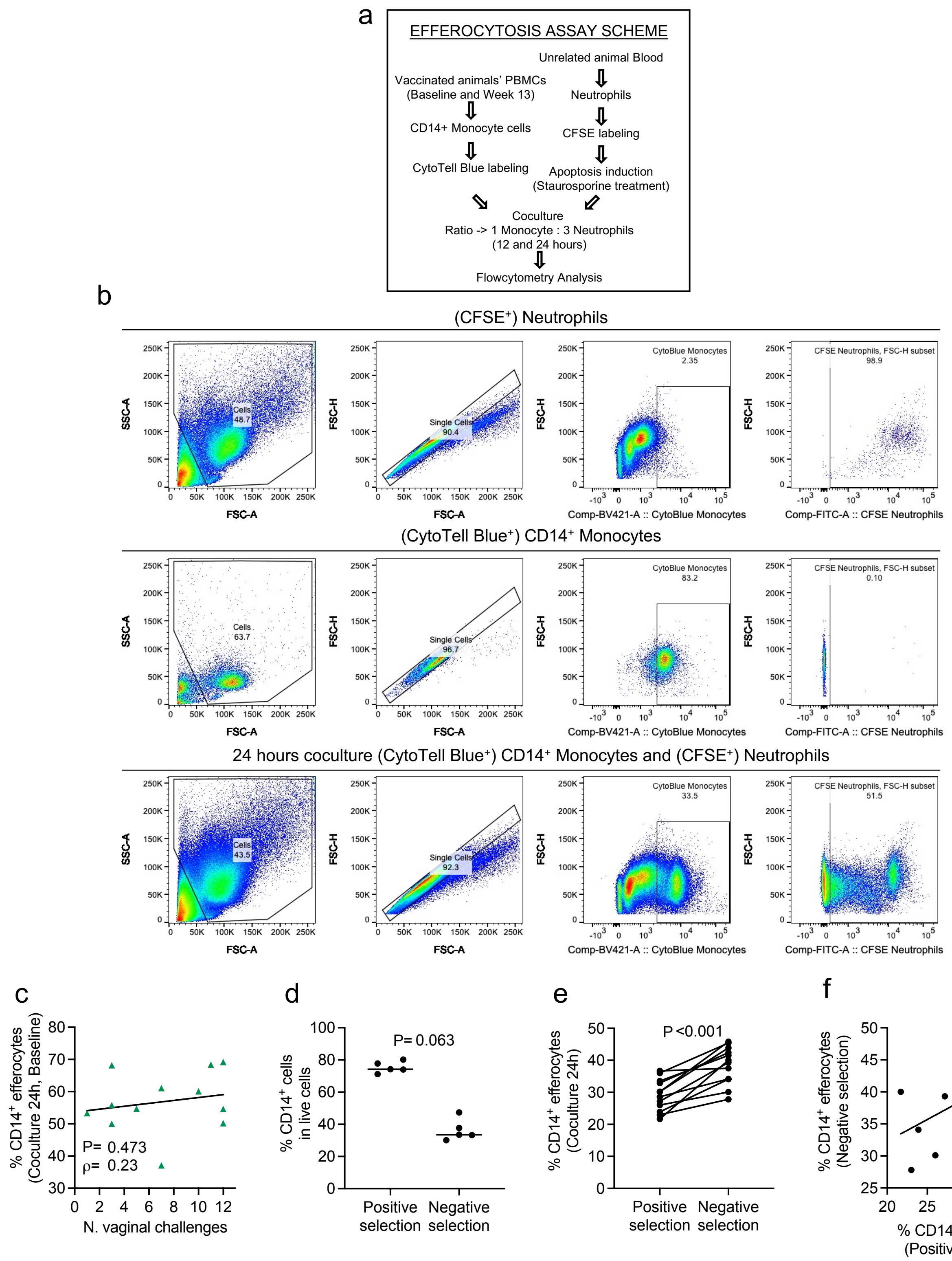

Supplementary Figure 7

g

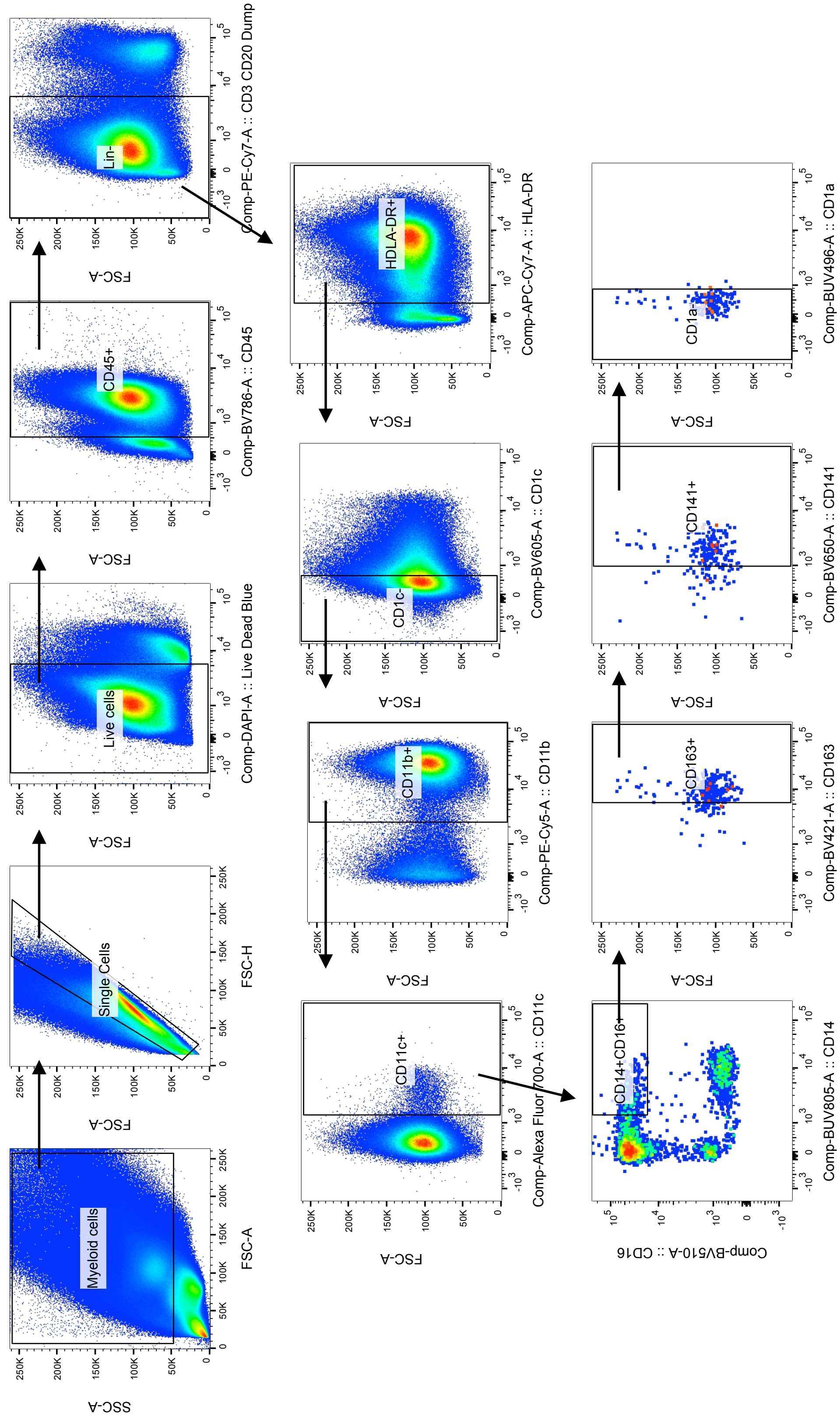

Supplementary Figure 7

Supplementary Figure S7. Efferocytosis assay and time of acquisition. (**a**) Schematic representation of efferocytosis assay performed to determine the frequency of CD14^{+} efferocytes in vaccinated animals. (**b**) Representative flow plot showing the gating strategy for identifying CD14^{+} efferocytes that engulfed the apoptotic neutrophils. Cells were gated on monocytes/neutrophils, singlets, CytoTell Blue^{+} and CFSE^{+}. The frequency of CD14^{+} efferocytes was expressed as the frequency of CFSE^{+} cells in CytoTell Blue^{+} cells. The CFSE^{+} Neutrophils (upper panel) and CytoTell Blue^{+} CD14^{+} cells (middle panel) not cocultured were used as controls for setting the gates. An example of 24-hour cocultured CFSE^{+} Neutrophils and CytoTell Blue^{+} CD14^{+} cells are reported in the lower panel. (**c**) Correlation between the frequency of CD14^{+} efferocytes cocultured for 24h and time of acquisition in n=12 vaccinated animals at baseline. (**d**) Frequency of CD14^{+} cells obtained following isolation with positive or negative selection from n=5 naïve animals (p=0.063; two-tailed Wilcoxon test). CD14^{+} cells were identified as Lin^{-}(CD3^{-}CD20^{-}CD8^{-})HLA-DR^{+}CD14^{+} and expressed as frequency of live cells. (**e**) Frequencies of CD14^{+} efferocytes in CD14^{+} cells isolated with positive or negative selection from PBMCs collected from n=12 naïve animals and cocultured for 24h (p=0.00049; two-tailed Wilcoxon test). (**f**) Correlation between the frequency of CD14^{+} efferocytes in CD14^{+} cells from n=12 animals and isolated with positive or negative selection and cocultured for 24h. (**g**) Representative flow plot showing the gating strategy for identifying DC-10. DC-10 cells were identified as (high SSC/Singlets/Live CD45^{+}/CD3^{-}CD20^{-}/HLA-DR^{+}/ CD1c^{-}/CD11b^{+}/CD11c^{+}/CD14^{+}CD16^{+}/CD163^{+}/CD141^{+}/CD1a^{-}) and expressed as the frequency of the cells in the final gate (CD1a^{-}) of the cells in CD11b^{+} gate. For visualization purposes the dots in the last 4 panels were enlarged. Correlation analyses: (**c, f**) two-tailed Spearman correlation test

and simple linear regression line. Displayed p values are unadjusted. Source data are provided in the Source Data file.

Hypothetical mechanisms of vaccine efficacy

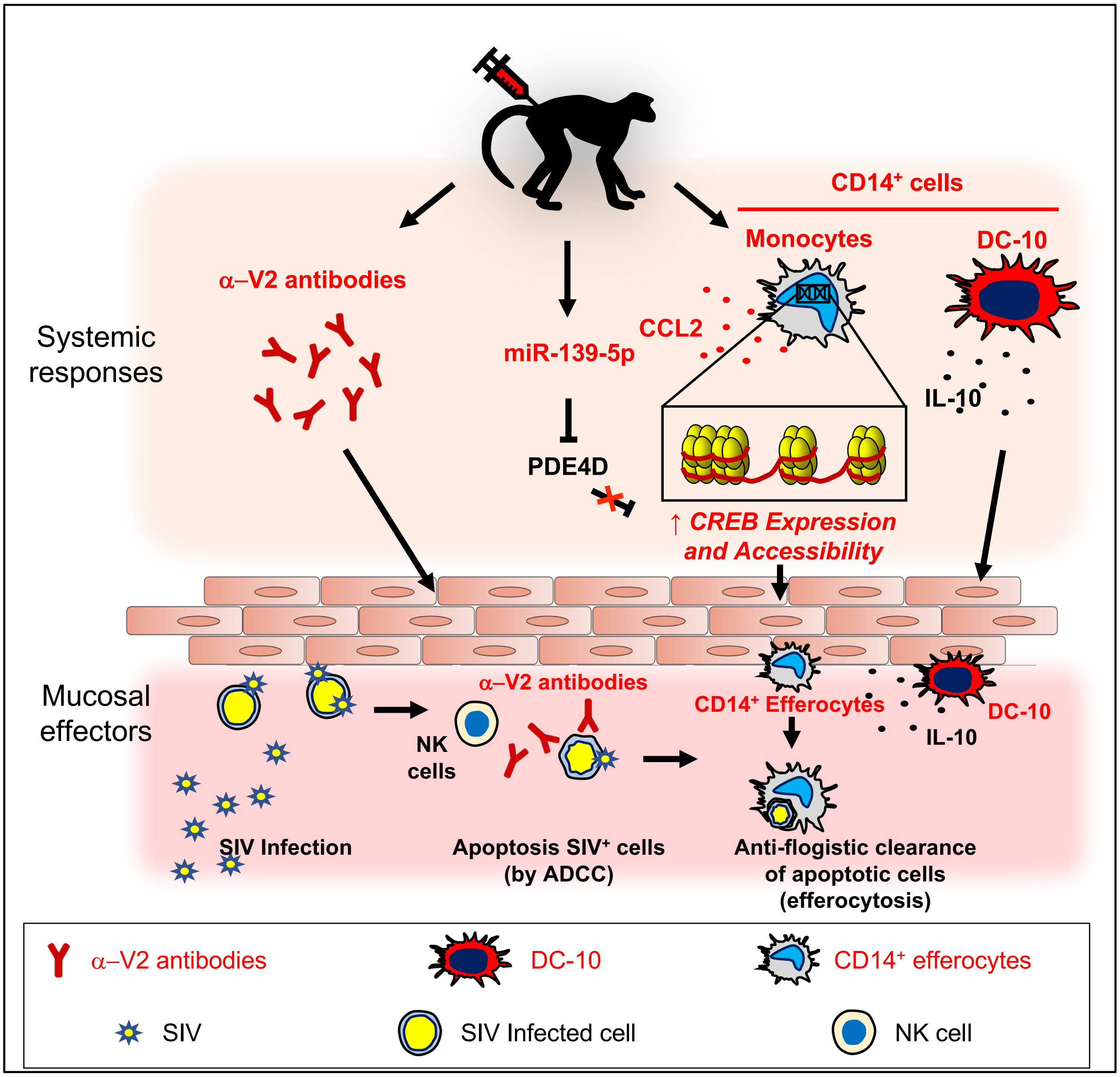

Supplementary Figure 8

Supplementary Figure S8. Hypothetical mechanisms of vaccine efficacy. Hypothetical schematic representation of systemic and mucosal events contributing to decreased risk of SIV_{mac251} acquisition in vaccinated animals. At the systemic level, vaccination induces the production of anti-V2 antibodies which mediate ADCC. The level of systemic miR-139-5p would contribute to activation of the CREB pathway by decreasing the levels of *PDE4D*. Following vaccination, $CD14^+$ efferocytic cells may migrate to the mucosa via the CCL2/CCR2 axis. Early after SIV_{mac251} exposure, mucosal cells that become infected would be targeted by Natural Killer cells, which mediate V2-specific ADCC. Apoptotic infected cells then may be engulfed by mucosal $CD14^+$ efferocytes, clearing the infection sites while maintaining an anti-inflammatory environment and avoiding recruitment of additional target cells. The monkey element in the schematic has been adapted from Silva de Castro*, et al.*[4].

a

		Young				Old				Young vs Old
		25% Percentile	Median	75% Percentile	95% CI	25% Percentile	Median	75% Percentile	95% CI	Mann-Whitney Test P-value
Neutralization ID50	TIER 1A SIVmac251.6 (ID#1636DB2)	65296	88146	166015	65121-171251	35220	100917	195052	36335-158033	0.7416
	TIER 1A SIVsmE660/BR-CG7G.IR1 (ID#1370DB2)	5427	26438	95260	5318-116559	1785	13990	44290	2204-38177	0.1698
	TIER 1B SIVsmE660/BR-CG7V.IR1 (ID#1634DB2)	0	880	5639	0-6480	0	0	685	0-593	0.1080
	**Challenge virus SIVmac251 DAY 8 2010 (ID#2079DB2)**	**46.5**	**63**	**112**	**46-119**	**0**	**44**	**55**	**0-52**	**0.0162**
Neutralization ID80	**TIER 1A SIVmac251.6 (ID#1636DB2)**	**0**	**1352**	**5673**	**0-5816**	**0**	**0**	**0**	**0-0**	**0.0002**
	TIER 1A SIVsmE660/BR-CG7G.IR1 (ID#1370DB2)	755.5	5847	22169	523-22477	504.5	4591	11249	732-10178	0.2629
	TIER 1B SIVsmE660/BR-CG7V.IR1 (ID#1634DB2)	0	0	0	0-0	0	0	0	0-0	>0.9999
	Challenge virus SIVmac251 DAY 8 2010 (ID#2079DB2)	0	0	0	0-0	0	0	0	0-0	>0.9999

b

		Young		Old		Young+Old	
		ρ	P	ρ	P	ρ	P
Neutralization ID50	TIER 1A SIVmac251.6 (ID#1636DB2)	-0.25	0.4077	0.16	0.5406	0.00	0.9846
	TIER 1A SIVsmE660/BR-CG7G.IR1 (ID#1370DB2)	-0.34	0.2508	0.13	0.6247	-0.03	0.8676
	TIER 1B SIVsmE660/BR-CG7V.IR1 (ID#1634DB2)	**-0.70**	**0.0112**	-0.27	0.2958	**-0.40**	**0.0287**
	Challenge virus SIVmac251 DAY 8 2010 (ID#2079DB2)	-0.50	0.0837	-0.38	0.1323	-0.33	0.0760
Neutralization ID80	TIER 1A SIVmac251.6 (ID#1636DB2)	**-0.56**	**0.0492**	all negative	all negative	-0.15	0.4325
	TIER 1A SIVsmE660/BR-CG7G.IR1 (ID#1370DB2)	-0.36	0.2303	0.13	0.6178	-0.09	0.6456
	TIER 1B SIVsmE660/BR-CG7V.IR1 (ID#1634DB2)	all negative	all negative	all negative	all negative	all negative	all negative
	Challenge virus SIVmac251 DAY 8 2010 (ID#2079DB2)	all negative	all negative	all negative	all negative	all negative	all negative

Supplementary Table 1

Supplementary Table S1. Serum neutralizing antibody responses to SIV viruses. (**a**) The data represented show the median, the 25th and 75th percentiles, and the 95% Confidence Intervals (CI) of ID_{50} and ID_{80} neutralizing antibody responses to different SIV viruses (TIER 1A/SIV_{mac251}, TIER 1A/SIV_{smE660}, TIER 1B/SIV_{smE660} and SIV_{mac251} used for the animal challenge) in serum of n=13 young and n=17 old animals at week 14. The last column shows the unadjusted p-values of the comparisons between the two groups using two-tailed Mann-Whitney test. Rows highlighted in red or blue indicate a higher response in young or old animals, respectively. (**b**) The data represented show the ρ and unadjusted p-values of the two-tailed Spearman correlations between the ID_{50} and ID_{80} neutralizing antibody responses and the number of SIV_{mac251} exposure required for infection in n=13 young, n=17 old, or n=13+17 combined groups. Cells highlighted in red indicate a significant correlation. Source data are provided in the Source Data file.

		Young				Old				Young vs Old
		25% Percentile	Median	75% Percentile	95% CI	25% Percentile	Median	75% Percentile	95% CI	Mann-Whitney Test P-value
Baseline	**IFNγ**	**0**	**0**	**0**	**0-0**	**0.435**	**3.58**	**4.81**	**0.87-4.7**	**0.0002**
	IL-2	0	0	4.71	0-5.05	0	1.91	7.14	0-4.37	0.5353
	**IL-10**	**0**	**0**	**0**	**0-0**	**0**	**107.6**	**294.1**	**0-275.5**	**0.0026**
	IL-17A	0.06	0.31	0.595	0-0.69	0.17	0.69	1.845	0.22-1.71	0.1344
	IL-13	0	0	0	0-0	0	0	0	0-0	0.8883
	**IL-1β**	**0**	**0**	**0.11**	**0-0.11**	**0.11**	**0.4**	**1.24**	**0.11-1.24**	**0.0028**
	IL-4	0	0	0	0-0	0	0	0	0-0	>0.9999
	IL-6	0	0	0.69	0-0.69	0	1.04	2.69	0-2.21	0.0517
	IL-8	1228	1858	2050	968-2084	829	1388	1752	830-1638	0.0786
	**CCL3**	**3.63**	**3.63**	**5.25**	**3.63-5.25**	**6.51**	**8.53**	**12.24**	**6.51-10.93**	**0.00006**
	CCL2	110.5	150.7	166.5	103.7-169.8	105.7	124.4	199.2	109.4-188.6	0.9016
	IL-22	0	0	0	0-0	0	0	0	0-0	>0.9999
	IL-21	0	0	0	0-0	0	0	0	0-0	>0.9999
	IL-23	0	0	0	0-0	0	0	0	0-0	0.2379
	**TGF-β1**	**93324**	**102164**	**113322**	**92044-118444**	**50775**	**69326**	**94956**	**51379-93603**	**0.0003**
	**TGF-β2**	**8772**	**10085**	**12520**	**8703-12542**	**5246**	**6942**	**8627**	**5257-8563**	**0.00006**
	TGF-β3	0	0	0	0-0	0	0	0	0-0	>0.9999

		Young				Old				Young vs Old
		25% Percentile	Median	75% Percentile	95% CI	25% Percentile	Median	75% Percentile	95% CI	Mann-Whitney Test P-value
Week 13	**IFNγ**	**2.49**	**3.75**	**5.8**	**2.28-6.21**	**4.055**	**6.62**	**13.61**	**4.57-10.64**	**0.0398**
	IL-2	0	0	4.315	0-4.92	0	3.71	12.09	0-10.55	0.3025
	IL-10	9.885	19.75	27.37	9.31-27.96	10.46	18.58	35.05	10.46-29.14	0.8121
	IL-17A	0.655	1.03	2.105	0.6-2.86	0.44	0.82	3.4	0.49-3.29	0.8933
	IL-13	9.595	21.18	23.41	6.53-24.33	11.38	15.73	27.97	12.66-24.91	0.9095
	IL-1β	0.37	0.59	1.15	0.26-1.23	0.395	0.8	1.515	0.48-1.49	0.8934
	IL-4	0	0	1.535	0-3.07	0	0	3.07	0-3.07	0.6586
	**IL-6**	**1.82**	**3.3**	**5.34**	**1.82-5.42**	**0.235**	**0.66**	**1.615**	**0.32-0.99**	**0.0008**
	**IL-8**	**807.3**	**1037**	**1403**	**740.6-1459**	**1095**	**2225**	**2991**	**1356-2948**	**0.0105**
	CCL3	7.445	9.95	12.77	6.96-13.91	6.96	9.2	15.71	6.96-14.42	0.8283
	**CCL2**	**137**	**174.5**	**227.1**	**133.2-229.6**	**94.88**	**122.2**	**148**	**96.06-141.1**	**0.006**
	IL-22	0	0	0	0-0	0	0	0	0-0	>0.9999
	IL-21	0	0	0	0-0	0	0	0	0-0	>0.9999
	IL-23	0	0	0	0-0	0	0	0	0-0	0.1793
	**TGF-β1**	**18116**	**22869**	**30085**	**17201-31288**	**19930**	**40225**	**56833**	**24363-47514**	**0.0037**
	**TGF-β2**	**2062**	**2642**	**3469**	**2050-3646**	**2344**	**3434**	**5867**	**2582-5641**	**0.017**
	TGF-β3	0	0	0	0-0	0	0	0	0-0	>0.9999

Supplementary Table 2

Supplementary Table S2. Cytokine/Chemokine levels in plasma of young and old macaques. The data represented show the median, the 25th and 75th percentiles, and the 95% confidence intervals (CI) of each cytokine/chemokine measured by Luminex in plasma of n=13 young and n=17 old animals at baseline and 1 week following the last immunization (week 13). The last column shows the unadjusted p-values of the comparisons between the two groups using the two-tailed Mann-Whitney test. Rows highlighted in red or blue indicate higher expression in young or old animals, respectively. Source data are provided in the Source Data file.

		Young				Old				Young vs Old
		25% Percentile	Median	75% Percentile	95% CI	25% Percentile	Median	75% Percentile	95% CI	Mann-Whitney Test P-value
Not stimulated	GM-CSF	0.72	1.7	3.555	0.58-5.15	0.18	0.22	1.54	0.18-2.22	0.159
	**TGF-α**	**0.28**	**0.73**	**0.95**	**0.28-0.95**	**0**	**0**	**0.14**	**0-0.28**	**0.024**
	G-CSF	6.735	13.65	18.4	4.9-19.95	0	0.55	9.275	0-13.65	0.063
	IFN-γ	2.6	10.56	16.69	0-22.82	0	0	1.2	0-1.81	0.071
	IL-2	9.96	29.44	50.28	7.82-62.39	1.59	1.91	26.04	1.43-44.47	0.095
	IL-10	26.45	48.34	120.4	11.49-139.1	0	0	40.91	0-68.03	0.095
	IL-15	1.015	1.46	3.39	0.79-3.88	0	0.3	2.62	0-4.45	0.175
	sCD40L	15.68	20.81	49.01	12.24-62.12	3.355	5.19	27.51	1.52-35.9	0.159
	**IL-17A**	**0.465**	**0.89**	**3.545**	**0.28-5.71**	**0**	**0**	**0.405**	**0-0.53**	**0.024**
	IL-1ra	81.48	154.5	401.1	18.92-634	4.965	9.64	143.9	4.54-274.7	0.095
	IL-13	8.14	24.88	26.22	7.16-26.88	0	4.47	9.475	0-10.75	0.056
	IL-1β	1.345	1.81	3.175	0.98-3.28	0.11	0.26	1.975	0.06-3.17	0.095
	**IL-4**	**3.75**	**14.93**	**14.93**	**3.75-14.93**	**0**	**0**	**5.05**	**0-9.47**	**0.032**
	IL-5	0.095	3.32	6.08	0-6.79	0	0	0.255	0-0.51	0.087
	**IL-6**	**2.545**	**4.04**	**10.22**	**1.15-16.39**	**0.39**	**0.72**	**1.93**	**0.39-3.03**	**0.016**
	**IL-8**	**836.7**	**1780**	**2079**	**438.1-2183**	**72.14**	**129.8**	**673.1**	**41.72-843.5**	**0.032**
	**CCL3**	**3.12**	**5.36**	**6.86**	**3.12-8.36**	**0**	**0**	**2.68**	**0-5.36**	**0.04**
	CCL2	124.1	450.2	806.9	93.08-1027	51.96	52.98	568.8	51.06-1075	0.151
	**TNF-α**	**12.52**	**16.4**	**69.44**	**10.56-72.65**	**0**	**0**	**6.255**	**0-10.56**	**0.016**
	CCL4	0.255	1.45	2.435	0-2.73	0	0	0.255	0-0.51	0.087
	IL-12/23p40	0	7.28	10.19	0-10.19	0	0	2.145	0-4.29	0.167
	VEGF	39.71	73.7	119.3	19.88-125.9	5.895	40.18	106.4	0-165.8	0.31
	IL-18	3.865	9.6	13.56	2.3-13.93	1.03	1.84	6.285	0.68-9.6	0.063
LPS stimulated	**GM-CSF**	**24.37**	**28.07**	**44.96**	**21.33-61.12**	**12.05**	**13.28**	**22.14**	**11.79-23.46**	**0.016**
	**TGF-α**	**2.64**	**3.65**	**3.985**	**2.08-4.1**	**0.84**	**1.85**	**2.075**	**0.5-2.3**	**0.016**
	G-CSF	96.1	751.4	1832	69.23-2362	159.4	780.3	2062	48.76-2496	0.841
	**IFN-γ**	**15.23**	**16.76**	**22.31**	**14.71-24.81**	**6.265**	**8.44**	**14.2**	**4.09-14.71**	**0.016**
	IL-2	66.83	73.17	82.96	64.88-86.95	42.32	55.11	67.58	35.69-69.38	0.056
	IL-10	885.1	1414	2024	791-2236	333.3	979.3	2271	102.9-3295	0.46
	IL-15	5.38	6.67	7.205	4.64-7.38	2.235	5.2	7	0.79-8.8	0.278
	sCD40L	57.23	68.59	87.87	49.09-87.87	25.8	49.09	62.12	15.69-65.36	0.063
	**IL-17A**	**3.41**	**3.66**	**3.97**	**3.35-4.22**	**1.38**	**2.11**	**2.915**	**0.89-3.1**	**0.008**
	IL-1ra	498.8	673.9	828.7	487.3-979.9	103	152	981.8	72.4-1765	0.151
	IL-13	27.16	29.35	34.18	25.56-34.56	18.34	25.56	31.45	16.74-33.26	0.246
	IL-1β	120.4	234.1	478.6	106.3-639.5	68.64	138.4	403.2	18.63-425.7	0.841
	IL-4	33.74	38.14	42.5	31.84-45.6	13.45	30.57	39.38	6.66-43.12	0.175
	**IL-5**	**4.42**	**5.21**	**7.84**	**4.26-10.31**	**0.975**	**2.06**	**2.53**	**0.82-3**	**0.008**
	IL-6	389	554	1068	279.4-1267	148.3	195	1167	135.8-1871	0.222
	IL-8	70338	88647	103338	68704-107498	8710	52893	81240	2873-102431	0.095
	**CCL3**	**811.3**	**1366**	**1902**	**637-2248**	**211.7**	**446.4**	**636**	**17.32-663.6**	**0.016**
	CCL2	2577	2615	2655	2543-2662	683	1475	2413	539.3-2671	0.151
	TNF-α	334.3	516.6	781.8	328.3-919.5	119.1	308.2	515.9	116-594.5	0.151
	**CCL4**	**95.49**	**135.8**	**220.6**	**67.48-255**	**42.1**	**58.99**	**116.4**	**36.57-120.1**	**0.032**
	**IL-12/23p40**	**40.17**	**49.35**	**57.1**	**34.89-63.55**	**15.79**	**24.13**	**33.54**	**10.19-37.54**	**0.016**
	VEGF	296.1	573.8	1006	225.4-1220	80.96	154.3	890.4	65.36-1396	0.31
	**IL-18**	**36.45**	**39.6**	**45.78**	**35.61-47.76**	**18.61**	**29.71**	**36.98**	**17.16-38.76**	**0.032**

Supplementary Table 3

Supplementary Table S3. Cytokine/Chemokine expression in PBMCs of young and old macaques. The data represented show the median, the 25th and 75th percentiles, and the 95% confidence intervals (CI) of each cytokine/chemokine measured by Luminex in supernatants of PBMCs collected from n=5 young and n=5 old naïve macaques and stimulated with or without LPS for 24h. The last column shows the unadjusted p-values of the comparisons between the young and old animals using the two-tailed Mann-Whitney test. Rows highlighted in red or blue indicate higher expression in young or old animals, respectively. Source data are provided as a Source Data file.

GROUP/TIME-POINT	CONTRAST	MODULE NAME	GS DETAILS	NES	NOM p-val	FDR q-val
**Young (Baseline)**	challenge	EM131_FOURATI_BIOAGE_MODULE6	T-cell receptor signaling	-1.7	<0.001	**0.0129**
**Old (Week 12+24h / Baseline)**	challenge	EM131_FOURATI_BIOAGE_MODULE9	Fatty acid biosynthesis	-1.88	<0.001	**0.00182**
**Old (Week 12+24h)**	challenge	EM131_FOURATI_BIOAGE_MODULE9	Fatty acid biosynthesis	-1.64	0.00606	**0.0087**
**Old (Baseline)**	challenge	EM131_FOURATI_BIOAGE_MODULE9	Fatty acid biosynthesis	-1.53	0.00345	**0.0228**
**Young (Week 12+24h / Baseline)**	challenge	EM131_FOURATI_BIOAGE_MODULE9	Fatty acid biosynthesis	1.75	0.00236	**0.0043**
**Young (Week 12+24h / Baseline)**	challenge	EM131_FOURATI_BIOAGE_MODULE10	AA metabolism	1.53	0.0117	**0.0301**
**Old (Baseline)**	challenge	EM131_FOURATI_BIOAGE_MODULE13	NA	1.64	<0.001	**0.0177**
**Young (Week 12+24h / Baseline)**	challenge	EM131_FOURATI_BIOAGE_MODULE13	NA	1.87	<0.001	**0.00315**
**Young (Week 12+24h)**	challenge	EM131_FOURATI_BIOAGE_MODULE13	NA	1.8	<0.001	**0.001**
**Young (Week 12+24h / Baseline)**	challenge	EM131_FOURATI_BIOAGE_MODULE16	Inflammation	-1.53	0.00351	**0.0259**
**Old (Week 12+24h / Baseline)**	challenge	EM131_FOURATI_BIOAGE_MODULE17	Type II interferon signaling	1.96	<0.001	**0.00321**
**Old Week (12+24h)**	challenge	EM131_FOURATI_BIOAGE_MODULE17	Type II interferon signaling	1.59	0.00149	**0.0128**
**Young (Week 12+24h / Baseline)**	challenge	EM131_FOURATI_BIOAGE_MODULE17	Type II interferon signaling	-1.58	0.0103	**0.0321**
**Old (Baseline)**	challenge	EM131_FOURATI_BIOAGE_MODULE19	NK mediated cytolysis	1.7	<0.001	**0.0134**
**Young (Week 12+24h / Baseline)**	challenge	EM131_FOURATI_BIOAGE_MODULE19	NK mediated cytolysis	1.57	0.00711	**0.0293**
**Young (Week 12+24h)**	challenge	EM131_FOURATI_BIOAGE_MODULE19	NK mediated cytolysis	1.93	<0.001	**0.001**

Method: GSEA

Associated with younger age (red)
Associated with older age (cyan)

Supplementary Table 4

Supplementary Table S4. BioAge modules and risk of SIV acquisition. The table lists BioAge modules that correlated with risk of SIV acquisition in vaccinated animals. Data are presented as correlation of the expression of genes grouped in the different BioAge modules for each group/time-point in contrast to numbers of challenges required to infection (Challenge). Normalized Enrichment Scores (NES) values indicate the positive or negative correlation. P-values are reported as unadjusted (NOM p-val) or adjusted for multiple comparisons (FDR). Modules M9, M10, M16 and M17 are highlighted. Modules associated with younger or older age are highlighted in red or blue, respectively. To identify genes regulated by the vaccine and correlated with the number of SIV challenges to infection, a two-tailed linear regression model was fitted with the number of SIV challenges to infection as an independent variable and the fold-change post/pre of the genes as a dependent variable using the R-package LIMMA[5]. A moderated t-test was used to test that the coefficient of regression was statistically different from zero. The Benjamini-Hochberg method was used to correct the p-values for multiple testing (adjusted p-values). Genes with an adjusted p-value below 5% were considered significantly correlated with challenges. Source data are provided at GEO Series accession number GSE188901 (https://www.ncbi.nlm.nih.gov/geo/query/acc.cgi?acc=GSE188901).

a

		Gene ID	Gene name	logFC	P-Value	FDR
Young	Young Baseline	ENSMMUG00000019205	*ADAMTS16*	1.134	1.01E-07	0.00213
		ENSMMUG00000018058	*ABCG1*	-0.119	2.92E-06	0.03084
	Young Week 12+24h	ENSMMUG00000021288	*ANKRD28*	-0.154	2.51E-07	0.00271
		ENSMMUG00000020349	*SEL1L3*	-0.159	2.60E-07	0.00271
		ENSMMUG00000000672	*HP*	-0.597	1.15E-06	0.00765
		ENSMMUG00000005442	*NT5E*	-0.361	3.75E-06	0.01324
		ENSMMUG00000019205	*ADAMTS16*	0.946	3.81E-06	0.01324
		ENSMMUG00000015151	*IL2RA*	-0.230	7.44E-06	0.02214
Old	Old Baseline	ENSMMUG00000012251	*POU2F3*	1.238	2.29E-10	0.00001
		ENSMMUG00000021160	*ADAMTS17*	-0.181	6.76E-08	0.00074
		ENSMMUG00000012841	*KLK3*	1.155	9.80E-08	0.00074
		ENSMMUG00000023780	*IFI27*	-0.234	8.25E-06	0.03101
	Old Week 12+24h	ENSMMUG00000016649	*TNFRSF10D*	-0.434	6.74E-08	0.00077
		ENSMMUG00000018011	*SPHK1*	0.337	8.08E-07	0.00459
		ENSMMUG00000000238	*HECTD2*	-0.337	1.77E-06	0.00806
		ENSMMUG00000011777	*B3GNT7*	0.134	3.25E-06	0.01232
		ENSMMUG00000021977	*PI16*	0.113	7.29E-06	0.02368
		ENSMMUG00000004622	*NLRP6*	0.166	1.25E-05	0.03561
		ENSMMUG00000043275	*BDKRB2*	0.184	1.87E-05	0.04245

(green) correlated with decreased risk of acquisition
(orange) correlated with increased risk of acquisition

b

		Correlated with reduced risk of acquisition	Correlated with increased risk of acquisition
Young	Baseline	*ADAMTS16*	*ABCG1*
	Week 12+24h	*ADAMTS16*	*ANKRD28, SEL1L3, HP, NT5E, IL2RA*
	Week 12+24h / Baseline	none	*ZC3H7A*
Old	Baseline	*POU2F3, KLK3*	*ADAMTS17, IFI27*
	Week 12+24h	*SPHK1, B3GNT7, PI16, NLRP6, BDKRB2*	*TNFRSF10D, HECTD2*
	Week 12+24h / Baseline	none	none

Supplementary Table 5

Supplementary Table S5. Gene expression and risk of SIV acquisition. (**a**) The data represented show the RNA transcripts and their IDs identified in the whole blood collected at baseline and 24h following the last immunization (week 12+24h) that are correlated with a decreased (green rows) or increased (red rows) risk of SIV_{mac251} acquisition either in n=13 young or n=17 old animals. Transcripts are divided based on the time-point of collection. Each gene is associated to a fold-change (log_2FC), unadjusted p-value, and FDR adjusted for multiple comparisons. (**b**) Summary table showing gene transcripts that are correlated with reduced (green) or increased (red) risk of SIV_{mac251} acquisition in n=13 young and n=17 old animals at the different time-points. To identify genes regulated by the vaccine and correlated with the number of SIV challenges to infection, a two-tailed linear regression model was fitted with the number of SIV challenges to infection as an independent variable and the fold-change post/pre of the genes as a dependent variable using the R-package LIMMA[5]. A moderated t-test was used to test that the coefficient of regression was statistically different from zero. The Benjamini-Hochberg method was used to correct the p-values for multiple testing (adjusted p-values). Genes with an adjusted p-value below 5% were considered significantly correlated with challenges. Source data are provided at GEO Series accession number GSE188901 (https://www.ncbi.nlm.nih.gov/geo/query/acc.cgi?acc=GSE188901).

miRNA	Young Average	Young SD	Young 25% Percentile	Young Median	Young 75% Percentile	Young 95% CI of median	Old Average	Old SD	Old 25% Percentile	Old Median	Old 75% Percentile	Old 95% CI of median	Fold Change Old-Young	Log Fold Change Old-Young	T stat Old-Young	P value Old-Young	Adjusted P value Old-Young
mml-miR-455-5p	4.08	0.964	3.03	4.31	4.76	2.92 - 4.8	1.69	0.953	1.18	1.64	2.09	1.1 - 2.1	-5.17	-2.37	-6.91	8.31E-08	4.26E-05
mml-miR-98	8.55	0.516	8.17	8.43	8.96	8.15 - 8.98	9.73	0.503	9.38	9.55	10.3	9.37 - 10.3	2.28	1.19	6.17	6.84E-07	0.0002
mml-miR-454-3p	7.67	0.732	7.07	7.81	8.25	6.94 - 8.32	9.06	0.602	8.6	9.08	9.72	8.59 - 9.75	2.62	1.39	5.74	2.37E-06	0.0003
mml-miR-411-5p	7.61	0.709	7.19	7.62	8.2	7.17 - 8.23	9.42	0.981	8.64	9.37	10	8.59 - 10	3.48	1.80	5.68	2.83E-06	0.0003
mml-miR-379-5p	8.37	0.511	8.04	8.4	8.78	8.02 - 8.81	9.68	0.701	9.16	9.78	10.1	9.14 - 10.1	2.49	1.32	5.67	2.88E-06	0.0003
mml-miR-193a-5p	6.37	0.824	5.69	6.44	6.81	5.57 - 6.82	4.82	0.691	4.4	4.93	5.25	4.36 - 5.25	-2.90	-1.53	-5.61	3.48E-06	0.0003
mml-miR-151-5p	6.17	0.368	5.93	6.1	6.45	5.92 - 6.46	7.16	0.497	6.71	7.25	7.49	6.7 - 7.49	1.98	0.98	5.59	3.69E-06	0.0003
mml-miR-214-3p	5.64	0.726	5.25	5.68	6.11	5.24 - 6.21	3.91	1.05	3.16	3.75	4.88	3.06 - 5.08	-3.30	-1.72	-5.35	7.40E-06	0.0004
mml-miR-409-5p	5.97	0.745	5.49	6.13	6.29	5.48 - 6.31	7.19	0.5	6.96	7.18	7.6	6.96 - 7.6	2.33	1.22	5.33	7.78E-06	0.0004
mml-let-7a-5p	14.5	0.492	14.4	14.5	14.8	14.4 - 14.8	15.4	0.417	15.1	15.5	15.7	15.1 - 15.7	1.82	0.86	5.13	1.37E-05	0.0007
mml-let-7f-5p	13.8	0.656	13.5	13.7	14.4	13.5 - 14.4	14.9	0.511	14.3	14.9	15.4	14.3 - 15.4	2.10	1.07	5.11	1.47E-05	0.0007
mml-miR-142-3p	12.8	0.905	12.1	12.9	13.6	12.1 - 13.6	14.2	0.697	13.5	14.2	14.9	13.5 - 14.9	2.65	1.41	4.98	2.12E-05	0.0009
mml-miR-370-3p	6.27	1.02	5.37	6.34	7.08	5.29 - 7.14	8.28	1.2	7.16	8.5	9.06	7.11 - 9.12	4.00	2.00	4.97	2.20E-05	0.0009
mml-miR-190a-5p	5.93	0.753	5.55	6	6.4	5.52 - 6.41	7.61	1.08	6.67	7.37	8.49	6.64 - 8.57	3.19	1.67	4.85	3.15E-05	0.0011
mml-let-7d	11.1	0.449	10.8	11	11.4	10.8 - 11.4	12	0.517	11.6	12	12.4	11.6 - 12.4	1.86	0.89	4.83	3.28E-05	0.0011
mml-miR-125b-5p	10.4	0.236	10.2	10.4	10.6	10.2 - 10.6	9.64	0.501	9.18	9.6	9.88	9.14 - 9.88	-1.68	-0.75	-4.73	4.37E-05	0.0014
mml-miR-615-3p	1.52	0.666	1.06	1.55	2.07	1.02 - 2.11	0.342	0.645	-0.14	0.152	0.672	-0.155 - 0.675	-2.29	-1.19	-4.70	4.79E-05	0.0014
mml-miR-205	5.82	0.959	5.11	5.64	6.68	5.08 - 6.82	3.96	1.3	3.07	3.84	4.79	3 - 5.05	-3.64	-1.86	-4.68	5.06E-05	0.0014
mml-miR-20a-3p	2.89	0.495	2.32	3.05	3.26	2.3 - 3.31	3.76	0.389	3.46	3.8	4.06	3.45 - 4.08	1.81	0.86	4.66	5.40E-05	0.0015
mml-miR-660-5p	9.14	0.422	8.88	9	9.53	8.88 - 9.62	8.24	0.581	7.89	8.28	8.76	7.89 - 8.79	-1.86	-0.89	-4.59	6.52E-05	0.0017
mml-miR-93-5p	14.3	0.268	14	14.3	14.4	14 - 14.4	13.7	0.276	13.5	13.6	13.8	13.5 - 13.8	-1.42	-0.51	-4.42	0.0001	0.0026
mml-miR-32-3p	3	0.636	2.76	3.08	3.46	2.71 - 3.47	3.87	0.408	3.64	3.77	4.15	3.62 - 4.17	1.82	0.87	4.22	0.0002	0.0044
mml-miR-145-5p	5.11	0.792	4.69	5.24	5.46	4.68 - 5.46	3.65	1.11	2.78	3.66	4.54	2.71 - 4.66	-2.72	-1.44	-4.18	0.0002	0.0048
mml-miR-130a-3p	7.11	0.224	6.96	7.09	7.29	6.96 - 7.33	6.57	0.342	6.38	6.51	6.9	6.37 - 6.93	-1.45	-0.54	-4.10	0.0003	0.0057
mml-miR-375	6.31	0.978	6.03	6.38	7.02	6.03 - 7.06	4.39	1.64	3.67	4.38	5.52	3.65 - 5.54	-3.73	-1.90	-4.04	0.0003	0.0065
mml-miR-542-3p	3.64	0.39	3.29	3.68	3.92	3.26 - 3.94	2.92	0.477	2.71	2.97	3.19	2.66 - 3.19	-1.66	-0.73	-3.99	0.0004	0.0068
mml-miR-1277	4.49	0.521	4.22	4.42	4.89	4.2 - 4.91	5.25	0.426	4.94	5.15	5.54	4.92 - 5.57	1.67	0.74	3.99	0.0004	0.0068
mml-miR-28-5p	5.2	0.478	5	5.15	5.45	4.98 - 5.48	5.97	0.515	5.62	5.94	6.44	5.58 - 6.46	1.70	0.77	3.97	0.0004	0.0071
mml-miR-599-5p	4.09	0.373	3.84	4.01	4.31	3.84 - 4.35	4.83	0.516	4.42	4.93	5.08	4.38 - 5.09	1.67	0.74	3.93	0.0004	0.0076
mml-miR-140-3p	9.16	0.376	8.88	9.18	9.45	8.85 - 9.46	8.58	0.371	8.28	8.53	8.87	8.23 - 8.88	-1.50	-0.59	-3.90	0.0005	0.0079
mml-miR-218-5p	2.61	0.982	1.71	2.51	3.7	1.63 - 3.75	1.48	0.594	1.13	1.63	1.87	1.09 - 1.87	-2.21	-1.14	-3.89	0.0005	0.0079
mml-miR-340-3p	3.68	0.337	3.45	3.71	4.02	3.44 - 4.07	4.38	0.489	3.93	4.52	4.69	3.85 - 4.7	1.62	0.70	3.87	0.0005	0.0082
mml-miR-149-5p	2.45	0.844	1.87	2.18	3.23	1.87 - 3.41	1.32	0.742	0.727	1.44	2.05	0.69 - 2.08	-2.18	-1.12	-3.81	0.0006	0.0088
mml-miR-551a	1.14	0.798	0.393	0.98	1.94	0.334 - 1.99	0.23	0.463	-0.0964	0.137	0.645	-0.0964 - 0.662	-1.89	-0.92	-3.80	0.0006	0.0088
mml-miR-1185-5p	3.99	0.603	3.69	3.98	4.38	3.68 - 4.42	4.86	0.595	4.47	4.88	5.45	4.47 - 5.46	1.82	0.86	3.80	0.0006	0.0088
mml-miR-365-3p	5.24	0.344	5.05	5.26	5.43	5.04 - 5.44	4.4	0.739	3.79	4.27	5.14	3.76 - 5.18	-1.78	-0.83	-3.80	0.0006	0.0088
mml-miR-374a-3p	3.31	0.9	2.57	3.21	4	2.45 - 4.08	4.48	0.757	4.08	4.56	5.06	4.05 - 5.08	2.22	1.15	3.79	0.0006	0.0088
mml-let-7b-3p	4.06	0.398	3.89	4.13	4.32	3.87 - 4.35	3.07	0.873	2.94	3.23	3.64	2.93 - 3.69	-1.97	-0.98	-3.77	0.0007	0.0090
mml-miR-429-3p	4.57	1.08	3.67	4.63	5.47	3.64 - 5.49	3.09	1.15	2.44	3.42	3.98	2.43 - 4.11	-2.75	-1.46	-3.72	0.0008	0.0101
mml-miR-379-3p	3.89	0.807	3.82	3.9	4.02	3.8 - 4.04	4.85	0.583	4.49	5.03	5.32	4.49 - 5.37	1.93	0.95	3.71	0.0008	0.0102
mml-miR-7	9.61	0.65	9.15	9.5	9.86	9.12 - 9.88	10.4	0.56	10	10.3	11	9.98 - 11	1.74	0.80	3.61	0.0011	0.0131
mml-miR-378a	8.94	0.43	8.57	9	9.31	8.53 - 9.33	8.33	0.435	8.01	8.35	8.67	8 - 8.68	-1.52	-0.60	-3.59	0.0011	0.0132
mml-miR-127-3p	5.79	0.955	5.29	5.64	6.2	5.26 - 6.29	7.11	1.11	6.35	6.74	7.6	6.31 - 7.7	2.54	1.34	3.59	0.0011	0.0132
mml-miR-544	3.51	0.768	3.09	3.68	4.09	3.08 - 4.11	4.42	0.555	4.02	4.3	4.85	4.01 - 4.88	1.83	0.87	3.57	0.0012	0.0135
mml-miR-6827-5p	4.22	0.579	3.64	4.35	4.78	3.64 - 4.84	5.02	0.595	4.56	5.2	5.44	4.5 - 5.46	1.74	0.80	3.56	0.0012	0.0136
mml-miR-329-2-5p	0.17	0.546	-0.167	-0.0218	0.45	-0.179 - 0.489	0.939	0.545	0.492	1.12	1.34	0.421 - 1.36	1.70	0.76	3.54	0.0012	0.0139
mml-miR-103-3p	12.5	0.573	12	12.5	13	12 - 13	13.1	0.374	12.9	13.1	13.4	12.9 - 13.4	1.54	0.62	3.53	0.0013	0.0141
mml-miR-125b-2-3	3.18	0.556	2.81	3.21	3.61	2.8 - 3.68	2.3	0.714	1.58	2.65	2.87	1.51 - 2.92	-1.81	-0.86	-3.50	0.0014	0.0152
mml-miR-1306-5p	9.16	0.524	8.78	9.22	9.57	8.68 - 9.61	8.37	0.682	7.83	8.44	8.97	7.77 - 9.01	-1.73	-0.79	-3.47	0.0015	0.0159
mml-miR-192-5p	8.72	0.654	8.06	8.97	9.23	8.06 - 9.29	7.81	0.78	7.18	7.57	8.3	7.14 - 8.37	-1.88	-0.91	-3.46	0.0015	0.0159
mml-miR-100-5p	4.9	0.585	4.38	4.95	5.44	4.32 - 5.52	4.01	0.78	3.38	4.03	4.66	3.31 - 4.66	-1.85	-0.88	-3.46	0.0016	0.0159
mml-miR-412-5p	4.14	0.701	3.68	4.19	4.55	3.68 - 4.6	5.01	0.675	4.6	5.2	5.46	4.55 - 5.52	1.82	0.86	3.41	0.0018	0.0175
mml-miR-495-3p	5.51	0.495	5.09	5.46	5.72	5.04 - 5.76	6.18	0.533	5.92	6.26	6.51	5.89 - 6.53	1.59	0.67	3.36	0.0020	0.0195

Supplementary Table 6 (1/2)

miRNA	Young						Old						Fold Change	Log Fold Change	T stat	P value	Adjusted P value
	Average	SD	25% Percentile	Median	75% Percentile	95% CI of median	Average	SD	25% Percentile	Median	75% Percentile	95% CI of median	Old-Young	Old-Young	Old-Young	Old-Young	Old-Young
mml-miR-23a-3p	13.8	0.345	13.5	13.8	14.2	13.5 - 14.2	13.3	0.418	13	13.4	13.5	13 - 13.5	-1.41	-0.50	-3.36	0.0021	0.0195
mml-miR-500b-5p	1.12	0.812	0.254	1.28	1.64	0.142 - 1.64	2.09	0.782	1.47	2.12	2.43	1.4 - 2.48	1.99	0.99	3.35	0.0021	0.0196
mml-miR-489-3p	0.855	0.824	-0.03	1.15	1.58	-0.0721 - 1.62	0.0753	0.443	-0.179	-0.0843	0.197	-0.179 - 0.216	-1.72	-0.79	-3.26	0.0027	0.0236
mml-miR-424-3p	3.06	0.357	2.75	3.07	3.3	2.68 - 3.34	2.24	0.831	2.1	2.37	2.71	2.08 - 2.75	-1.76	-0.82	-3.26	0.0027	0.0236
mml-miR-30d-5p	13.5	0.721	12.9	13.6	13.9	12.9 - 14	12.8	0.334	12.4	12.9	13.1	12.4 - 13.2	-1.56	-0.64	-3.26	0.0027	0.0236
mml-miR-1235	3.9	0.845	3.42	4.15	4.49	3.31 - 4.52	2.73	1.17	1.83	2.67	3.88	1.74 - 4.01	-2.26	-1.17	-3.23	0.0029	0.0250
mml-miR-24-3p	12.9	0.515	12.7	12.9	13.3	12.7 - 13.3	12.4	0.331	12.2	12.4	12.6	12.2 - 12.6	-1.43	-0.51	-3.19	0.0032	0.0264
mml-miR-874-3p	4.05	0.742	3.82	4.22	4.46	3.78 - 4.49	3.24	0.646	2.63	3.33	3.6	2.55 - 3.6	-1.76	-0.82	-3.18	0.0033	0.0264
mml-miR-320b	6.45	0.418	6.18	6.33	6.84	6.17 - 6.9	5.95	0.345	5.65	5.93	6.19	5.65 - 6.21	-1.41	-0.50	-3.18	0.0033	0.0264
mml-miR-508-3p	0.588	1.84	-0.129	0.0329	0.456	-0.129 - 0.489	3.24	2.8	0.771	2.42	6.21	0.662 - 6.29	6.54	2.71	3.17	0.0033	0.0264
mml-miR-155	12.5	0.607	11.9	12.4	13.2	11.9 - 13.2	13.2	0.654	12.9	13.2	13.6	12.9 - 13.6	1.66	0.73	3.17	0.0034	0.0264
mml-miR-491-5p	6.31	0.514	6.06	6.41	6.66	6.05 - 6.67	6.98	0.599	6.56	7.06	7.48	6.49 - 7.53	1.59	0.67	3.17	0.0034	0.0264
mml-miR-452-5p	2.41	0.631	2.14	2.44	2.64	2.06 - 2.64	1.4	0.976	0.343	1.65	1.97	0.142 - 1.97	-2.01	-1.01	-3.17	0.0034	0.0264
mml-miR-150-5p	13.1	0.62	12.5	13	13.6	12.5 - 13.6	12.3	0.778	11.8	12.2	12.8	11.8 - 12.9	-1.75	-0.81	-3.16	0.0034	0.0264
mml-miR-125a-5p	11.6	0.417	11.5	11.8	11.9	11.5 - 12	11.1	0.497	10.9	11.1	11.5	10.9 - 11.5	-1.47	-0.55	-3.15	0.0035	0.0265
mml-miR-221-5p	5.51	0.351	5.17	5.55	5.78	5.09 - 5.79	6.13	0.609	5.86	6.05	6.45	5.83 - 6.46	1.54	0.62	3.13	0.0038	0.0278
mml-miR-92a-3p	15	0.331	14.8	15	15.1	14.8 - 15.1	14.6	0.364	14.4	14.7	14.8	14.4 - 14.8	-1.34	-0.43	-3.12	0.0038	0.0278
mml-miR-150-3p	5.43	0.511	5.01	5.55	5.92	4.99 - 5.94	4.74	0.658	4.29	4.8	5.24	4.24 - 5.26	-1.61	-0.69	-3.08	0.0043	0.0310
mml-miR-181d	5.99	0.335	5.73	5.87	6.26	5.72 - 6.33	6.47	0.424	6.25	6.44	6.7	6.25 - 6.7	1.40	0.48	3.06	0.0045	0.0321
mml-miR-887	2.48	0.665	1.96	2.67	2.87	1.96 - 2.87	1.52	0.928	0.849	1.54	2.1	0.766 - 2.1	-1.91	-0.93	-3.02	0.0050	0.0345
mml-miR-9-5p	5.5	1.25	4.72	5.27	5.76	4.72 - 5.79	6.73	1.07	5.81	6.64	7.3	5.78 - 7.4	2.35	1.23	3.02	0.0050	0.0345
mml-miR-143-3p	9.6	1.02	8.65	9.51	10.4	8.56 - 10.5	8.39	1.24	7.52	8.19	9.46	7.43 - 9.5	-2.30	-1.20	-3.00	0.0052	0.0348
mml-miR-942-5p	7.62	0.316	7.29	7.65	7.95	7.26 - 8	8.01	0.292	7.77	7.92	8.3	7.76 - 8.35	1.32	0.40	3.00	0.0052	0.0348
mml-miR-134-5p	9.7	0.436	9.3	9.74	10	9.25 - 10	10.3	0.599	9.96	10.4	10.7	9.92 - 10.7	1.52	0.60	2.99	0.0053	0.0348
mml-miR-671-3p	6.75	0.463	6.41	6.74	7.14	6.37 - 7.17	6.19	0.501	5.65	6.22	6.68	5.65 - 6.7	-1.47	-0.56	-2.99	0.0053	0.0348
mml-miR-539	-0.0997	0.222	-0.337	-0.101	0.0963	-0.356 - 0.0963	0.461	0.578	-0.0904	0.275	1.03	-0.0964 - 1.1	1.47	0.56	2.95	0.0059	0.0379
mml-miR-30d-3p	4.43	0.211	4.23	4.49	4.61	4.19 - 4.61	4.96	0.527	4.44	4.94	5.29	4.38 - 5.31	1.43	0.52	2.95	0.0060	0.0379
mml-miR-514a-3p	0.145	1.06	-0.345	-0.215	0.0963	-0.356 - 0.0963	1.83	2.01	0.0406	1.04	3.87	-0.0178 - 3.91	3.20	1.68	2.95	0.0060	0.0379
mml-miR-30a-5p	9.84	0.47	9.51	9.71	10.2	9.46 - 10.3	9.09	0.845	8.39	9.29	9.69	8.37 - 9.71	-1.68	-0.75	-2.94	0.0061	0.0380
mml-miR-296-3p	4.55	0.367	4.29	4.48	4.8	4.28 - 4.81	4.05	0.42	3.76	4.11	4.36	3.74 - 4.36	-1.40	-0.49	-2.93	0.0063	0.0386
mml-miR-889-5p	-0.0534	0.299	-0.263	-0.153	0.0689	-0.279 - 0.0963	0.576	0.669	-0.0661	0.542	1.09	-0.0721 - 1.1	1.54	0.62	2.92	0.0064	0.0386
mml-miR-484	8.86	0.544	8.45	9	9.14	8.36 - 9.14	8.15	0.753	7.88	8.25	8.63	7.84 - 8.68	-1.64	-0.71	-2.92	0.0064	0.0386
mml-miR-329-1-5p	2.28	0.746	1.57	2.3	2.87	1.54 - 2.97	3.09	0.731	2.55	3.09	3.88	2.55 - 3.95	1.76	0.81	2.91	0.0065	0.0386
mml-miR-885-3p	2.5	0.786	1.79	2.62	3.1	1.62 - 3.23	1.41	1.17	0.221	1.31	2.45	0.216 - 2.48	-2.13	-1.09	-2.91	0.0066	0.0386
mml-miR-195-3p	1.43	0.812	0.956	1.24	2.11	0.944 - 2.23	0.63	0.67	0.147	0.416	0.995	0.142 - 1.09	-1.75	-0.81	-2.90	0.0066	0.0386
mml-miR-204-3p	1.21	1.04	0.361	1.32	1.57	0.0963 - 1.62	0.394	0.441	-0.0768	0.408	0.821	-0.0964 - 0.839	-1.76	-0.81	-2.90	0.0067	0.0386
mml-miR-486-5p	15.5	0.36	15.1	15.7	15.7	15.1 - 15.7	14.9	0.69	14.3	14.8	15.7	14.3 - 15.7	-1.51	-0.60	-2.90	0.0068	0.0386
mml-miR-758-5p	1.04	0.651	0.649	0.979	1.41	0.645 - 1.5	2	1.03	1.59	2.01	2.66	1.54 - 2.83	1.93	0.95	2.89	0.0069	0.0389
mml-miR-339-3p	7.48	0.376	7.22	7.45	7.83	7.2 - 7.84	7.08	0.282	6.91	7.06	7.31	6.9 - 7.33	-1.32	-0.40	-2.87	0.0073	0.0405
mml-miR-154-3p	0.716	0.765	0.0963	0.539	1.48	0.0963 - 1.58	1.53	0.79	0.916	1.77	2.01	0.839 - 2.06	1.78	0.83	2.86	0.0074	0.0405
mml-miR-145-3p	2.03	0.817	1.35	1.76	2.8	1.31 - 2.92	0.991	1.1	0.0107	0.697	2.03	-0.0178 - 2.08	-2.06	-1.04	-2.86	0.0074	0.0405
mml-miR-1240	1.74	0.482	1.34	1.76	2.08	1.3 - 2.12	1.01	0.786	-0.0136	1.34	1.62	-0.0721 - 1.65	-1.68	-0.75	-2.84	0.0078	0.0409
mml-miR-128b-3p	9.67	0.286	9.47	9.78	9.92	9.46 - 9.92	9.28	0.357	8.95	9.39	9.55	8.91 - 9.55	-1.31	-0.39	-2.84	0.0078	0.0409
mml-miR-1185-3p	2.76	0.552	2.24	2.95	3.21	2.11 - 3.25	4.01	1.46	2.75	3.92	5.2	2.66 - 5.29	2.34	1.23	2.84	0.0079	0.0409
mml-miR-376b-5p	2.25	0.701	1.75	2.27	2.85	1.66 - 2.85	2.89	0.459	2.53	2.86	3.16	2.52 - 3.17	1.56	0.64	2.84	0.0079	0.0409
mml-miR-148a-5p	7.37	0.433	7.14	7.37	7.68	7.08 - 7.7	6.89	0.422	6.49	7.01	7.1	6.46 - 7.11	-1.39	-0.48	-2.84	0.0079	0.0409
mml-miR-330-5p	2.12	0.252	1.97	2.12	2.32	1.94 - 2.34	2.71	0.62	2.38	2.65	3.11	2.37 - 3.14	1.50	0.58	2.81	0.0085	0.0434
mml-miR-1262-3p	3.01	0.871	2.19	3.08	3.68	2.12 - 3.77	2.13	0.856	1.42	2.08	2.65	1.4 - 2.66	-1.84	-0.88	-2.80	0.0085	0.0434
mml-miR-1271-5p	6.63	0.362	6.43	6.63	6.92	6.42 - 6.93	6.17	0.428	5.9	6.29	6.39	5.89 - 6.41	-1.37	-0.45	-2.80	0.0086	0.0434
mml-miR-381-5p	-0.075	0.244	-0.263	-0.129	0.094	-0.279 - 0.0963	0.52	0.672	-0.0768	0.259	1.04	-0.0964 - 1.09	1.50	0.58	2.78	0.0090	0.0448
mml-miR-222-3p	8.53	0.468	8.49	8.64	8.85	8.48 - 8.85	8.03	0.475	7.7	7.93	8.41	7.67 - 8.43	-1.42	-0.50	-2.78	0.0091	0.0448
mml-miR-143-5p	2.45	1.15	1.79	2.45	3.23	1.74 - 3.29	1.34	1.11	0.316	1.23	2.08	0.142 - 2.08	-2.17	-1.12	-2.76	0.0095	0.0464
mml-miR-299-5p	7.58	0.708	6.8	7.79	8	6.73 - 8.02	8.2	0.497	7.88	8.35	8.64	7.87 - 8.65	1.52	0.61	2.75	0.0097	0.0464
mml-miR-25	12.9	0.247	12.8	12.9	13.1	12.8 - 13.1	12.6	0.313	12.4	12.5	12.9	12.3 - 13	-1.26	-0.33	-2.75	0.0097	0.0464
mml-let-7i-5p	13.6	0.304	13.4	13.5	14	13.4 - 14.1	14	0.299	13.8	13.9	14.1	13.8 - 14.1	1.26	0.34	2.74	0.0099	0.0470
mml-miR-497-5p	4.6	0.514	4.22	4.68	4.83	4.1 - 4.86	4.05	0.518	3.56	4.13	4.44	3.54 - 4.45	-1.46	-0.55	-2.74	0.0100	0.0470

Supplementary Table 6 (2/2)

Supplementary Table S6. Differently expressed miRNAs in old and young animals. The table shows the average, standard deviation, median, the 25th and 75th percentiles, and the 95% confidence intervals (CI) of the quantile normalized levels of each miRNA in EVs of n=12 young and n=16 old animals at 1 week following the last immunization (week 13) that are significantly different between the two groups (adjusted p-values). The table reports the fold-change of each miRNA between the old and young animals, the log of the fold-change, t-test p-values of the comparisons between the old and young animals (R function t-test), and p-values adjusted for multiple comparisons. Differentially expressed genes between young and old animals were determined using Limma-Voom[5]. Adjusted p-values were generated by the Benjamini-Hochberg method. The table reports only the miRNAs that showed significant adjusted p-values. Source data are provided at GEO Series accession number GSE188575 (https://www.ncbi.nlm.nih.gov/geo/query/acc.cgi?acc=GSE188575).

a

DNA/ALVAC/gp120 Young (week 13)

microRNA	Mean	Std. Deviation	25% Percentile	Median	75% Percentile	95% CI of median	Hazard Ratio	Lower CI	Upper CI	P value	FDR
mml-miR-544	3.51	0.77	3.09	3.68	4.09	3.08 - 4.11	22.96	1.62	325.72	0.001	0.60
mml-miR-376c-3p	8.75	0.62	8.44	8.68	9.35	8.43 - 9.42	11.14	1.55	80.13	0.004	0.60
mml-miR-1255a-5p	0.63	0.74	0.01	0.65	1.31	-0.01 - 1.47	0.08	0.01	0.74	0.004	0.60
mml-miR-377-3p	7.78	0.62	7.15	7.77	8.35	7.10 - 8.35	10.95	1.44	83.55	0.006	0.60
mml-miR-494-3p	7.68	0.69	7.14	7.74	8.22	6.99 - 8.32	8.98	1.48	54.38	0.006	0.60
mml-miR-382-3p	6.18	0.64	5.63	6.26	6.59	5.61 - 6.61	6.59	1.31	33.18	0.013	0.65
mml-miR-23b-5p	5.12	0.33	4.86	5.08	5.41	4.86 - 5.42	0.01	0.00	0.88	0.014	0.65
mml-miR-3146	3.19	0.32	2.94	3.26	3.39	2.93 - 3.39	0.03	0.00	0.59	0.015	0.65
mml-miR-23a-3p	13.81	0.35	13.51	13.83	14.17	13.51 - 14.20	73.35	1.07	5032.51	0.016	0.65
mml-miR-30b-5p	6.92	0.34	6.64	7.04	7.17	6.59 - 7.17	103.40	0.61	17514.39	0.018	0.65
mml-miR-889-3p	8.53	0.41	8.15	8.51	8.83	8.07 - 8.88	14.20	1.31	154.15	0.019	0.65
mml-miR-3059-5p	-0.06	0.44	-0.37	-0.24	0.27	-0.38 - 0.33	0.01	0.00	2.77	0.019	0.65
mml-miR-4677-3p	0.14	0.49	-0.27	0.10	0.44	-0.28 - 0.49	8.39	1.44	48.79	0.019	0.65
mml-miR-660-3p	0.28	0.43	-0.10	0.23	0.58	-0.13 - 0.59	8.73	1.31	58.05	0.020	0.65
mml-miR-92b-5p	4.41	0.45	4.09	4.23	4.73	4.07 - 4.74	0.06	0.00	0.96	0.020	0.65
mml-let-7a-5p	14.54	0.49	14.42	14.47	14.76	14.42 - 14.79	0.05	0.00	1.04	0.020	0.65
mml-miR-98	8.55	0.52	8.17	8.44	8.96	8.15 - 8.98	0.09	0.01	0.96	0.024	0.69
mml-miR-7174-5p	1.81	0.91	1.36	1.54	2.66	1.34 - 2.68	3.47	1.02	11.73	0.026	0.69
mml-let-7e-5p	9.02	0.63	8.70	9.08	9.35	8.70 - 9.37	0.15	0.03	0.94	0.028	0.69
mml-miR-1185-5p	3.99	0.60	3.69	3.98	4.38	3.68 - 4.42	5.01	1.11	22.53	0.029	0.69
mml-miR-196a-5p	5.87	0.88	5.15	5.88	6.66	5.13 - 6.72	0.31	0.10	0.98	0.030	0.69
mml-miR-139-5p	4.87	0.43	4.57	4.77	5.35	4.56 - 5.42	0.09	0.01	1.05	0.031	0.69
mml-miR-29b-1-5p	0.87	0.60	0.61	0.93	1.18	0.59 - 1.18	0.19	0.04	0.91	0.033	0.69
mml-miR-656-3p	5.12	0.62	4.57	5.17	5.53	4.54 - 5.55	4.63	1.10	19.50	0.034	0.69
mml-let-7f-5p	13.83	0.66	13.51	13.74	14.39	13.51 - 14.42	0.19	0.04	0.98	0.034	0.69
mml-let-7f-3p	1.69	0.76	0.91	2.03	2.21	0.66 - 2.23	6.88	0.48	98.98	0.035	0.69
mml-miR-376c-5p	2.64	0.55	2.25	2.61	2.99	2.23 - 3.03	5.98	0.99	36.15	0.041	0.77
mml-miR-142-5p	12.89	0.54	12.44	12.85	13.30	12.43 - 13.38	4.24	1.05	17.16	0.046	0.77
mml-let-7b-5p	14.39	0.37	14.01	14.47	14.63	13.99 - 14.67	0.07	0.00	1.25	0.048	0.77
mml-miR-376a-3p	9.09	0.58	8.69	9.21	9.52	8.65 - 9.55	5.32	0.84	33.56	0.049	0.77

b

DNA/ALVAC/gp120 Young

	Baseline						Week 13						Correlation					Baseline vs Week 13
microRNA	Mean	Std. Deviation	25% Percentile	Median	75% Percentile	95% CI of median	Mean	Std. Deviation	25% Percentile	Median	75% Percentile	95% CI of median	Hazard Ratio	Lower CI	Upper CI	P value	FDR	Mann-Whitney Test P-value
mml-miR-3059-5p	0.12	0.44	-0.36	0.30	0.38	-0.52 - 0.71	-0.06	0.44	-0.37	-0.24	0.27	-0.38 - 0.33	0.01	0.00	2.77	0.019	0.65	0.468
mml-miR-23b-5p	5.20	0.98	5.11	5.37	5.84	3.11 - 5.95	5.12	0.33	4.86	5.08	5.41	4.86 - 5.42	0.01	0.00	0.88	0.014	0.65	0.167
mml-miR-3146	3.37	0.80	3.42	3.50	3.82	1.66 - 4.17	3.19	0.32	2.94	3.26	3.39	2.93 - 3.39	0.03	0.00	0.59	0.015	0.65	0.068
mml-let-7a-5p	14.72	0.26	14.48	14.67	14.83	14.48 - 15.24	14.54	0.49	14.42	14.47	14.76	14.42 - 14.79	0.05	0.00	1.04	0.020	0.65	0.134
mml-miR-92b-5p	4.90	0.44	4.55	4.69	5.20	4.51 - 5.72	4.41	0.45	4.09	4.23	4.73	4.07 - 4.74	0.06	0.00	0.96	0.020	0.65	0.065
mml-let-7b-5p	14.16	0.42	13.87	14.19	14.19	13.77 - 15.03	14.39	0.37	14.01	14.47	14.63	13.99 - 14.67	0.07	0.00	1.25	0.048	0.77	0.190
mml-miR-1255a-5p	0.38	0.78	-0.36	0.33	1.10	-0.52 - 1.39	0.63	0.74	0.01	0.65	1.31	-0.01 - 1.47	0.08	0.01	0.74	0.004	0.60	0.522
**mml-miR-139-5p**	**4.19**	**0.55**	**3.94**	**4.24**	**4.76**	**3.17 - 4.81**	**4.87**	**0.43**	**4.57**	**4.77**	**5.35**	**4.56 - 5.42**	**0.09**	**0.01**	**1.05**	**0.031**	**0.69**	**0.016**
**mml-miR-98**	**9.12**	**0.38**	**8.69**	**9.12**	**9.32**	**8.67 - 9.74**	**8.55**	**0.52**	**8.17**	**8.44**	**8.96**	**8.15 - 8.98**	**0.09**	**0.01**	**0.96**	**0.024**	**0.69**	**0.028**
mml-let-7e-5p	8.87	0.46	8.64	8.78	9.20	8.18 - 9.60	9.02	0.63	8.70	9.08	9.35	8.70 - 9.37	0.15	0.03	0.94	0.028	0.69	0.469
**mml-miR-29b-1-5p**	**0.46**	**0.45**	**0.33**	**0.56**	**0.56**	**-0.37 - 1.13**	**0.87**	**0.60**	**0.61**	**0.93**	**1.18**	**0.59 - 1.18**	**0.19**	**0.04**	**0.91**	**0.033**	**0.69**	**0.045**
mml-let-7f-5p	14.14	0.15	13.99	14.05	14.34	13.99 - 14.34	13.83	0.66	13.51	13.74	14.39	13.51 - 14.42	0.19	0.04	0.98	0.034	0.69	0.371
mml-miR-196a-5p	5.61	0.83	4.91	5.37	6.43	4.74 - 7.05	5.87	0.88	5.15	5.88	6.66	5.13 - 6.72	0.31	0.10	0.98	0.030	0.69	0.635

Supplementary Table 7

Supplementary Table S7. MicroRNAs correlating with susceptibility to SIV acquisition in young vaccinated animals. (**a**) The table shows the average, standard deviation, median, the 25th and 75th percentiles, and the 95% confidence intervals (CI) of the quantile normalized expression of the 30 miRNAs in EVs of n=12 young animals at 1 week following the last immunization (week 13) that are correlated with risk of SIV_{mac251} acquisition (unadjusted p-values). Risk of acquisition was measured by number of challenges survived before infection, tested with a Cox model survival analysis (R function coxph[6]). Hazard Ratio (HR) is indicative of risk of SIV acquisition. The table reports the HR, its lower and upper 95% confidence interval endpoints, the p-value associated with HR, and FDR adjusted for multiple comparisons. HR>1 is indicative of increased risk of SIV acquisition, whereas HR<1 is indicative of decreased risk of SIV acquisition. (**b**) The table shows the average, standard deviation, median, the 25th and 75th percentiles, and the 95% Confidence Intervals (CI) of the quantile normalized expression of the 13 miRNA in EVs of n=7 young animals at baseline and n=12 young animals at 1 week following the last immunization (week 13) that are correlated with a decreased risk of SIV_{mac251} acquisition (unadjusted p-values). The table reports the HR, its lower and upper 95% confidence interval endpoints, the p-value associated with HR, and FDR adjusted for multiple comparisons. The last column shows the unadjusted p-values of the comparisons between the baseline and week 13 time-points using two-tailed Mann-Whitney test. Rows highlighted in red or blue indicate higher or lower expression at week 13 compared to baseline, respectively. Source data are provided at GEO Series accession number GSE188575 (https://www.ncbi.nlm.nih.gov/geo/query/acc.cgi?acc=GSE188575).

DNA/ALVAC/gp120 Old (week 13)											
microRNA	Mean	Std. Deviation	25% Percentile	Median	75% Percentile	95% CI of median	Hazard Ratio	Lower CI	Upper CI	P value	FDR
mml-miR-215-5p	6.30	1.22	5.62	6.39	7.09	5.61 - 7.13	3.94	1.48	10.50	0.001	0.66
mml-miR-942-3p	1.91	0.93	1.58	2.18	2.44	1.53 - 2.46	0.27	0.11	0.67	0.004	0.75
mml-miR-10b-5p	10.22	0.73	9.53	10.06	10.46	9.42 - 10.46	5.14	1.53	17.24	0.006	0.75
mml-miR-146b-5p	5.08	0.60	4.61	5.11	5.52	4.60 - 5.60	4.90	1.53	15.67	0.006	0.75
mml-miR-628-3p	7.37	0.30	7.34	7.46	7.53	7.34 - 7.53	0.01	0.00	0.60	0.010	0.82
mml-miR-4716	0.56	0.72	0.01	0.23	1.06	-0.02 - 1.09	0.24	0.06	0.91	0.012	0.82
mml-miR-134-3p	0.99	1.00	-0.02	1.04	1.63	-0.07 - 1.64	0.42	0.19	0.93	0.020	0.82
mml-miR-582-5p	0.63	1.00	-0.08	0.31	1.47	-0.10 - 1.74	2.18	1.16	4.11	0.022	0.82
mml-miR-1262-3p	2.13	0.86	1.42	2.08	2.65	1.40 - 2.67	0.39	0.16	0.94	0.028	0.82
mml-miR-210-5p	2.26	1.01	1.84	2.53	2.98	1.79 - 3.08	0.44	0.22	0.90	0.032	0.82
mml-miR-382-5p	12.37	0.48	12.03	12.46	12.72	11.97 - 12.72	0.17	0.03	0.84	0.032	0.82
mml-miR-6529-5p	11.83	0.24	11.67	11.88	11.97	11.63 - 11.97	0.04	0.00	0.66	0.032	0.82
mml-miR-154-3p	1.53	0.79	0.92	1.77	2.01	0.84 - 2.06	0.37	0.15	0.94	0.035	0.82
mml-miR-130b-5p	4.72	0.67	4.38	4.88	5.24	4.34 - 5.26	0.34	0.12	0.92	0.038	0.82
mml-miR-4766-5p	3.23	0.64	2.85	3.39	3.59	2.78 - 3.59	0.37	0.15	0.88	0.040	0.82
mml-miR-192-5p	7.81	0.78	7.18	7.57	8.30	7.14 - 8.37	2.45	1.06	5.67	0.043	0.82
mml-miR-29b-3p	8.07	0.62	7.49	8.10	8.45	7.43 - 8.48	3.00	1.05	8.56	0.044	0.82
mml-miR-128a-5p	1.78	0.69	1.16	1.90	2.17	1.10 - 2.17	0.32	0.10	0.99	0.044	0.82
mml-miR-7204-3p	1.33	0.64	1.02	1.34	1.60	0.99 - 1.63	3.14	0.95	10.42	0.047	0.82
mml-miR-29b-2-5p	2.12	0.91	2.09	2.26	2.73	2.08 - 2.75	0.46	0.22	0.96	0.049	0.82
mml-miR-342-3p	9.82	0.75	9.20	9.78	10.42	9.14 - 10.52	2.26	1.01	5.03	0.050	0.82
mml-miR-7206-3p	1.52	0.84	1.02	1.34	1.98	0.99 - 2.08	3.05	1.02	9.13	0.050	0.82

Supplementary Table 8

Supplementary Table S8. MicroRNAs correlating with susceptibility to SIV acquisition in old vaccinated animals. The table shows the average, standard deviation, median, the 25% and 75% percentiles and the 95% confidence intervals (CI) of the quantile normalized expression of the 22 miRNAs in EVs of n=16 old animals at 1 week following the last immunization (week 13) that are correlated with risk of SIV_{mac251} acquisition (unadjusted p-values). Risk of acquisition was measured by number of challenges survived before infection, tested with a Cox model survival analysis (R function coxph[6]). Hazard Ratio is indicative of risk of SIV acquisition. The table reports the HR, its lower and upper 95% confidence interval endpoints, the p-value associated with HR and FDR adjusted for multiple comparisons. HR>1 is indicative of increased risk of SIV acquisition, whereas HR<1 is indicative of decreased risk of SIV acquisition. Source data are provided at GEO Series accession number GSE188575 (https://www.ncbi.nlm.nih.gov/geo/query/acc.cgi?acc=GSE188575).

GENE ID	Gene name
ENSMMUG00000061109	NA
ENSMMUG00000008531	FCAR
ENSMMUG00000053798	NA
ENSMMUG00000005461	SMCHD1
ENSMMUG00000052747	NA
ENSMMUG00000004677	AGTRAP
ENSMMUG00000011779	HEATR5A
ENSMMUG00000063718	KLF9
ENSMMUG00000020113	CMBL
ENSMMUG00000058309	NA
ENSMMUG00000009247	OTUD1
ENSMMUG00000021963	PCDHGB5
ENSMMUG00000005076	PDSS1
ENSMMUG00000055114	NA
ENSMMUG00000001210	NA
ENSMMUG00000023073	CCNJL
ENSMMUG00000012742	SLC6A8
ENSMMUG00000052123	NA
ENSMMUG00000061103	PIN1
ENSMMUG00000015306	CNN2
ENSMMUG00000020685	SERTAD3
ENSMMUG00000006058	TSPO
ENSMMUG00000018679	GAPDH
ENSMMUG00000010032	EDEM2
ENSMMUG00000017788	HOPX
ENSMMUG00000020972	MRPL37
ENSMMUG00000053234	Metazoa_SRP
ENSMMUG00000022996	HS1BP3
ENSMMUG00000012410	IGSF9B
ENSMMUG00000014835	ANKMY1
ENSMMUG00000023456	ERGIC3
ENSMMUG00000001806	ZUP1
ENSMMUG00000000170	SAMD9
ENSMMUG00000023778	FBXO32
ENSMMUG00000053630	Metazoa_SRP
ENSMMUG00000043316	Metazoa_SRP
ENSMMUG00000007905	VPS72
ENSMMUG00000020710	OS9
ENSMMUG00000060194	mml-mir-661
ENSMMUG00000021486	MRPL10
ENSMMUG00000060020	PTRHD1
ENSMMUG00000013586	NMT1
ENSMMUG00000004571	KIAA1109
ENSMMUG00000040562	TMCO3
ENSMMUG00000032760	NA
ENSMMUG00000053836	NA
ENSMMUG00000025809	NA
ENSMMUG00000059624	TMEM101
ENSMMUG00000033056	NA
ENSMMUG00000019611	ARRDC1
ENSMMUG00000053249	NA
ENSMMUG00000054438	NA
ENSMMUG00000020223	RNF113A
ENSMMUG00000020120	NAA80
ENSMMUG00000056273	U6
ENSMMUG00000020819	PPP2CB
ENSMMUG00000010257	SLC5A6

Supplementary Table 9

Supplementary Table S9. Vaccine-induced genes in animals with delayed risk of SIV acquisition. The table lists the gene IDs and names of the 57 genes identified with the pipeline (Supplementary Fig. 5b) adopted to select genes that were induced by vaccination in animals with high time of acquisition (TOA>8; n=5) but not in animals with low time of acquisition (TOA<8; n=7). NA indicates the gene name is not available. Source data are provided at GEO Series accession numbers GSE188879 (https://www.ncbi.nlm.nih.gov/geo/query/acc.cgi?acc=GSE188879) and GSE189032 (https://www.ncbi.nlm.nih.gov/geo/query/acc.cgi?acc=GSE189032).

a

TIME-POINT	CONTRAST	PATHWAY	NES	p-val	Adjusted p-val
Week 13 / Baseline	Challenge	GOBP_CAMP_MEDIATED_SIGNALING	-1.32	0.117	0.319
Week 13 / Baseline	Challenge	GOBP_CELLULAR_RESPONSE_TO_CAMP	1.22	0.207	0.443
Week 13 / Baseline	Challenge	GOBP_REGULATION_OF_CAMP_MEDIATED_SIGNALING	-1.23	0.199	0.434
Week 13	Challenge	GOBP_CELLULAR_RESPONSE_TO_CAMP	1.30	0.096	0.406
Week 13	Challenge	GOBP_RESPONSE_TO_CAMP	1.21	0.167	0.525
Baseline	Challenge	GOBP_CAMP_MEDIATED_SIGNALING	1.27	0.172	0.453

decreased risk of acquisition
increased risk of acquisition

b

TIME-POINT	CONTRAST	PATHWAY	NES	p-val	Adjusted p-val
Week 13 / Baseline	V2-ADCC	CREB_02	1.55	0.002	0.017
Week 13 / Baseline	V2-ADCC	GOBP_CAMP_MEDIATED_SIGNALING	-1.90	0.002	0.016
Week 13 / Baseline	V2-ADCC	TOMALKA_2022_45CREBCHIP	1.79	0.002	0.019

positively correlated with V2-ADCC
negatively correlated with V2-ADCC

c

TIME-POINT	CONTRAST	PATHWAY	NES	p-val	Adjusted p-val
Week 13	miR-139-5p	TAXCREB_01	1.47	0.009	0.066
Week 13 / Baseline	miR-139-5p	GOBP_CAMP_MEDIATED_SIGNALING	1.62	0.012	0.131
Week 13 / Baseline	miR-139-5p	MCCLUNG_CREB1_TARGETS_DN	1.94	0.001	0.014

positively correlated with plasma miR-139-5p
negatively correlated with plasma miR-139-5p

Supplementary Table 10

Supplementary Table S10. Cyclic AMP (cAMP) pathways correlated with risk of SIV_{mac251} acquisition and V2-specific ADCC in $CD14^+$ cells isolated from n=12 animals of Study 2. (**a**) Summary table showing Gene Ontology Biological Process (GOBP) related to cAMP and CREB pathways in $CD14^+$ cells that correlated (unadjusted p-values) with reduced (green) or increased (red) risk of SIV_{mac251} acquisition at the different time-points (Fold-change: Week 13/Baseline, Week 13, and Baseline). Positive and negative NES are indicative of decreased and increased risk of SIV_{mac251} acquisition, respectively. The last columns show the unadjusted and adjusted p-values of the correlations with the numbers of challenges required to infection. (**b**) Summary table showing GOBP related to cAMP and CREB pathways in $CD14^+$ cells (Fold-change: Week 13/Baseline) that correlated with increased (green) or decreased (red) V2-specific ADCC. Positive and negative NES are indicative of increased and positive V2-ADCC, respectively. The last columns show the unadjusted and adjusted p-values of the correlations with V2-ADCC. (**c**) Summary table showing GOBP related to cAMP and CREB pathways in $CD14^+$ cells (Fold-change: Week 13/Baseline) that correlated with increased (green) or decreased (red) miR-139-5p relative expression (Timepoints: Week 13 or fold-change Week 13/Baseline). Positive and negative NES are indicative of increased and positive miR-139-5p relative expression, respectively. The last columns show the unadjusted and adjusted p-values of the correlations with miR-139-5p relative expression. A generalized two-tailed linear model was fit to the normalized gene expression (dependent variable) with the time to acquisition, V2-ADCC, or miR-139-5p as independent variables. A moderated t-test and Benjamini-Hochberg correction were used to assess the significance of the correlation to acquisition, adjusting for multiple testing. Geneset Enrichment analysis, as implemented in the R-package fgsea, was performed on the gene list ranked by t-statistic to assess enrichment of MSigDB genesets (version 7.4). All

pathways for gene set enrichment analyses were derived from the validated database Molecular Signatures DataBase (MSigDB)[7,8]. Source data are provided at GEO Series accession number GSE189032 (https://www.ncbi.nlm.nih.gov/geo/query/acc.cgi?acc=GSE189032).

SUPPLEMENTARY REFERENCES

1. Beca, S., *et al.* Phosphodiesterase 4D regulates baseline sarcoplasmic reticulum Ca2+ release and cardiac contractility, independently of L-type Ca2+ current. *Circ Res* **109**, 1024-1030 (2011).
2. Ghosh, A.K., Hurd, T. & Hildebrandt, F. 3D spheroid defects in NPHP knockdown cells are rescued by the somatostatin receptor agonist octreotide. *Am J Physiol Renal Physiol* **303**, F1225-1229 (2012).
3. Chio, C.L., Lajiness, M.E. & Huff, R.M. Activation of heterologously expressed D3 dopamine receptors: comparison with D2 dopamine receptors. *Mol Pharmacol* **45**, 51-60 (1994).
4. Silva de Castro, I., *et al.* Anti-V2 antibodies virus vulnerability revealed by envelope V1 deletion in HIV vaccine candidates. *iScience* **24**, 102047 (2021).
5. Ritchie, M.E., *et al.* limma powers differential expression analyses for RNA-sequencing and microarray studies. *Nucleic Acids Res* **43**, e47 (2015).
6. Therneau, T.M. A Package for Survival Analysis in S. (2015).
7. Liberzon, A., *et al.* Molecular signatures database (MSigDB) 3.0. *Bioinformatics* **27**, 1739-1740 (2011).
8. Subramanian, A., *et al.* Gene set enrichment analysis: a knowledge-based approach for interpreting genome-wide expression profiles. *Proc Natl Acad Sci U S A* **102**, 15545-15550 (2005).
