# Supplementary material for: HIV vaccine candidate efficacy in female macaques mediated by cAMP-dependent efferocytosis and V2-specific ADCC
Source: Nat Commun. 2023 Feb 2;14:575. doi: 10.1038/s41467-023-36109-8 (PMC9894672; doi:10.1038/s41467-023-36109-8)
Supplement: Supplementary file 2 — Reporting Summary [file 41467_2023_36109_MOESM2_ESM.pdf]

Corresponding author(s): Genoveffa Franchini

Last updated by author(s): Jan 6, 2023

Reporting Summary

Nature Portfolio wishes to improve the reproducibility of the work that we publish. This form provides structure for consistency and transparency in reporting. For further information on Nature Portfolio policies, see our Editorial Policies and the Editorial Policy Checklist.

Statistics

For all statistical analyses, confirm that the following items are present in the figure legend, table legend, main text, or Methods section.

n/a	Confirmed	
☐	☒	The exact sample size (*n*) for each experimental group/condition, given as a discrete number and unit of measurement
☐	☒	A statement on whether measurements were taken from distinct samples or whether the same sample was measured repeatedly
☐	☒	The statistical test(s) used AND whether they are one- or two-sided<br>*Only common tests should be described solely by name; describe more complex techniques in the Methods section.*
☐	☒	A description of all covariates tested
☐	☒	A description of any assumptions or corrections, such as tests of normality and adjustment for multiple comparisons
☐	☒	A full description of the statistical parameters including central tendency (e.g. means) or other basic estimates (e.g. regression coefficient) AND variation (e.g. standard deviation) or associated estimates of uncertainty (e.g. confidence intervals)
☐	☒	For null hypothesis testing, the test statistic (e.g. *F*, *t*, *r*) with confidence intervals, effect sizes, degrees of freedom and *P* value noted<br>*Give P values as exact values whenever suitable.*
☒	☐	For Bayesian analysis, information on the choice of priors and Markov chain Monte Carlo settings
☐	☒	For hierarchical and complex designs, identification of the appropriate level for tests and full reporting of outcomes
☐	☒	Estimates of effect sizes (e.g. Cohen's *d*, Pearson's *r*), indicating how they were calculated

Our web collection on statistics for biologists contains articles on many of the points above.

Software and code

Policy information about availability of computer code

Data collection	no software was used
Data analysis	Prism 9 for macOS, Version 9.2.0 (283), July 15, 2021<br>FlowJo v10.1 (TreeStar, Inc.).<br>Microsoft Excel for Mac, Version 16.54<br>QIAGEN Ingenuity Pathway Analysis (QIAGEN IPA), version 01-20-04<br>Palantir Foundry platform (Palantir Technologies), version 5.341.0<br><br>For Whole blood transcriptome<br>- Trimmomatic (version 0.36)<br>- STAR aligner (version 2.5.3a)<br>- HTSeq (version 0.9.1)<br>- R package edgeR (version 3.30.3)<br>- R package LIMMA (version 3.44.3)<br>- Molecular Signatures Database gene sets (version 6.1). The GSEA Java desktop program was downloaded from the Broad Institute (http://www.broadinstitute.org/gsea/index.jsp)<br><br>For microRNA<br>- bcl2fastq software (version 0.10.1; Illumina, Inc.).

- Limma-Voom (Limma package version 3.38.3) [Ritchie, M.E., et al. limma powers differential expression analyses for RNA-sequencing and microarray studies. Nucleic Acids Res 43, e47 (2015)]
- Cox model (R function coxph) [Therneau, T.M. A Package for Survival Analysis in S. (2015)]

For ATAC-seq and CD14+ transcriptome
- Bcal2fastq (version 2.17)
- Trimmomatic (version 0.36)
- Cutadapt (version 1.18)
- PICARD tool (version 2.25.7)
- MACS2 (version 2.2.7.1)
- STAR aligner (version 2.7)
- Bowtie2 (version 2.2.6)
- DESeq2 R package (version 1.34.0)
- Homer tools (version 4.11.1)
- Subread function featureCouts: subread 2.0.3
- LIMMA framework in R (version 4.1.1)
- R package fgsea (version 1.18.0)
- MSigDB genesets (version 7.4)

For manuscripts utilizing custom algorithms or software that are central to the research but not yet described in published literature, software must be made available to editors and reviewers. We strongly encourage code deposition in a community repository (e.g. GitHub). See the Nature Portfolio guidelines for submitting code & software for further information.

Data

Policy information about availability of data

All manuscripts must include a data availability statement. This statement should provide the following information, where applicable:
- Accession codes, unique identifiers, or web links for publicly available datasets
- A description of any restrictions on data availability
- For clinical datasets or third party data, please ensure that the statement adheres to our policy

The sequencing data discussed in this publication have been deposited in NCBI's Gene Expression Omnibus (http://www.ncbi.nlm.nih.gov/geo) and are accessible through GEO Series accession numbers:
Whole blood RNA-seq (Study 1): GSE188901
https://www.ncbi.nlm.nih.gov/geo/query/acc.cgi?acc=GSE188901
microRNA-seq (Study 1): GSE188575
https://www.ncbi.nlm.nih.gov/geo/query/acc.cgi?acc=GSE188575

CD14+ ATAC-seq (Study 2): GSE188879
https://www.ncbi.nlm.nih.gov/geo/query/acc.cgi?acc=GSE188879
CD14+ RNA-seq (Study 2): GSE189032
https://www.ncbi.nlm.nih.gov/geo/query/acc.cgi?acc=GSE189032

The source codes for the whole blood RNA-Seq analysis (Study 1) and CD14+ cells RNA-seq analysis (Study 2) are available at: https://github.com/sekalylab/vb013. The source codes for the microRNA-Seq analysis (Study 1) are available at: https://github.com/NIDAP-Community/HIV-vaccine-candidate-efficacy. The source codes for the CD14+ cells RNA-Seq/ATAC-Seq analysis (Study 2) are available at: https://github.com/CBIIT/BM-Rhesus-NatureComm2023.git.

All the other data are available in the 'Source Data" file

Human research participants

Policy information about studies involving human research participants and Sex and Gender in Research.

Reporting on sex and gender	Research was not conducted in human participants
Population characteristics	Research was not conducted in human participants
Recruitment	Research was not conducted in human participants
Ethics oversight	Research was not conducted in human participants

Note that full information on the approval of the study protocol must also be provided in the manuscript.

Field-specific reporting

Please select the one below that is the best fit for your research. If you are not sure, read the appropriate sections before making your selection.

- [x] Life sciences
- [ ] Behavioural & social sciences
- [ ] Ecological, evolutionary & environmental sciences

For a reference copy of the document with all sections, see nature.com/documents/nr-reporting-summary-flat.pdf

Life sciences study design

All studies must disclose on these points even when the disclosure is negative.

Sample size	The samples size of each group was determined by consultation with our statistician. The studies were not powered to compare SIVmac251 viral acquisition following viral exposure between vaccinated groups but rather between vaccinated and control groups.<br>In Study 1 the power of the study was calculated based on previous results, in which animals vaccinated with DNA/DNA/ALVAC+gp120/ALVAC +gp120 showed a 52% vaccine efficacy (Vaccari et al., Nat Med, 2018). In young animals, the power of the study using 13 vaccinated and 27 control macaques and assuming 52% vaccine efficacy was 59%; whereas, in old animals, the power of the study using 17 vaccinated and 11 control macaques and assuming 52% vaccine efficacy was 29%<br>In study 2 the power of the study was calculated based on previous results (Vaccari et al., Nat Med, 2018 and young of study 2) and was estimated as 81% when using 12 vaccinated and 37 control macaques.<br>Sample size was determined also based on our previous nonhuman primate studies in which, in the majority of the cases, a n=12 in vaccinated groups allowed us to identify a significant reduction of risk of acquisition.<br>Based on statistician consultation, it was established that the comparisons of other outcomes most likely had adequate power, but power analyses were not performed for them.
Data exclusions	MicroRNA samples that showed significantly different read distribution compared to the other samples and a poor gene coverage were removed (T112_wk13 and H24G_wk13 in study 1)<br>ATAC-seq samples with Fraction Reads in called peak regions (FRIP) <5% were removed (BM266 in study 2). Remaining samples had all FRIP>10%
Replication	The nature of the samples analyzed in the present studies, the limited amount of each sample collected from each animal and the cost of the non-human primate studies do not allow us to replicate the experiments. In the reported assays the replicates are represented by each animal enrolled in the study. All the data have been obtained with validated assays that have been used in previous publish work.
Randomization	Study 1: macaques were assigned to four groups based on their major histocompatibility status, age, and weight. One MamuA01+ and one MamuB17+ animal were included in the vaccinated group of the young cohort, whereas two MamuA01+ animals were included in the vaccinated group of the old cohort.<br><br>Study 2: the study included only 1 group and therefore the animals were not randomized. The study included one MamuA01+ and one MamuB17+ animal.
Blinding	For macaques studies (immunizations and viral exposures): the investigators that prepared the reagents were unblinded whereas the veterinarian staff was blinded.<br>For immunological assays: during the execution and analyses of the experiments the investigators were blinded. Investigators were unblinded after generating the results in order to perform the final analyses of the data and correlation studies.

Reporting for specific materials, systems and methods

We require information from authors about some types of materials, experimental systems and methods used in many studies. Here, indicate whether each material, system or method listed is relevant to your study. If you are not sure if a list item applies to your research, read the appropriate section before selecting a response.

Materials & experimental systems

n/a	Involved in the study	
[ ]	[x]	Antibodies
[x]	[ ]	Eukaryotic cell lines
[x]	[ ]	Palaeontology and archaeology
[ ]	[x]	Animals and other organisms
[x]	[ ]	Clinical data
[x]	[ ]	Dual use research of concern

Methods

n/a	Involved in the study	
[x]	[ ]	ChIP-seq
[ ]	[x]	Flow cytometry
[x]	[ ]	MRI-based neuroimaging

Antibodies

Antibodies used

As reported in the Method section:
To identify monocytic myeloid cells in Study 1 the following antibodies were used: PE-Cy7 anti-CD3 (clone SP34-2; cat. #563916, 2.0 µl), PE-Cy7 anti-CD20 (clone 2H7; cat. #560735, 1.0 µl), BV786 anti-NHP-CD45 (clone D058-1283; cat. #563861, 3.0 µl), APC anti-CD14 (clone M5E2; cat. #561390, 7.5 µl), FITC anti-CD16 (clone 3G8; cat. #555406, 5.0 µl), BV421 anti-CD192 (CCR2) (clone 48607; cat. #564067, 3.0 µl), PE-CF594 anti-CD184 (CXCR4) (clone 12G5; cat. #562389, 5.0 µl), all from BD Biosciences (San Jose, CA), and HLA-DR-APC-Cy7 (clone L243; cat. #307618, 5.0 µl) from BioLegend (San Diego, CA).
To identify monocytic myeloid cells in Study 2 the following antibodies were used: FITC anti-CD103 (clone B-Ly7; cat. #11-1038-42, 5.0 µl), PerCP-eFluor710 anti-CCR7 (clone 3D12; cat. #46-1979-42, 5.0 µl) from Thermo Fisher Scientific; PE anti-CD33 (clone AC104.3E3; cat. #130-113-349, 2.0 µl) from Miltenyi Biotec (Cambridge, MA); PE-CF594 anti-CXCR4 (clone 12G5; cat. #562389, 5.0 µl), PE-Cy7 anti-CD3 (clone SP34-2; cat. #557749, 1.0 µl), PE-Cy7 anti-CD20 (clone 2H7; cat. #560735, 1.0 µl), APC anti-CCR2 (clone 48607; cat. #558406, 2.0 µl), APC-R700 anti-CD11c (clone 3.9; cat. #566610, 2.5 µl), APC-Cy7 anti-HLA-DR (clone L243; cat. #335796, 2.5 µl), BV421 anti-CD163 (clone GHI/61; cat. #562643, 2.5 µl), BV510 anti-CD16 (clone 3G8; cat. #563830, 2.5 µl), BV650 anti-CD141 (clone 1A4; cat. #740604, 5.0 µl), BV750 anti-PD-L1 (clone MIH1; cat. #746965, 5.0 µl), BV786 anti-CD45 (clone D058-1283; cat. #563861, 2.5 µl), BUV395 anti-CD73 (clone AD2; cat. #742636, 5.0 µl), BUV496 anti-CD1a (clone SK9; cat. #750320, 2.5 µl), BUV563 anti-CD80 (clone 2D10.4; cat. #751730, 2.5 µl), BUV661 anti-CD86 (clone 2331; cat. #741629, 5.0 µl), BUV737 anti-CX3CR1 (clone 2A9-1; cat. #749355, 2.5 µl), BUV805 anti-CD14 (clone M5E2; cat. #612902, 3.0 µl), all from BD Biosciences; PE-Cy5 anti-CD11b (clone ICRF44; cat. #301308, 1.0 µl), PE-Cy5 anti-CDc (clone L161; cat. #331538, 2.5 µl), all from BioLegend.

For CD14+ cells purity evaluation the following antibodies were used: PE-Cy7 anti-CD20 (clone 2H7; cat. #560735, 2.0 µl), Alexa700 anti-CD3 (clone SP34-2; cat. # 557917, 5.0 µl), APC anti-CD14 (clone M5E2; cat. #555399, 7.0 µl), FITC anti-CD16 (clone 3G8; cat. #555406, 5.0 µl) all from BD Biosciences (San Jose, CA).

For Neutrophils purity evaluation the following antibodies were used in previous assays: Alexa700 anti-CD3 (clone SP34-2; cat. # 557917, 3.0 µl), Alexa700 anti-CD20 (clone 2H7; cat. #560631, 3.0 µl), Alexa700 anti-CD8 (clone RPA-T8; cat. # 561453, 3.0 µl), BV786 anti-CD45 (clone D058-1283; cat. #563861, 3.0 µl) all from BD Biosciences (San Jose, CA); FITC anti-CD66abce (clone TET2; cat. #130-116-668) from Miltenyi Biotec (Cambridge, MA).

For negative selection of CD14+ cells: The following antibodies were used: anti-CD3 (clone SP34-2; cat. #552127, 15 µl), anti-CD8 (clone RPA-T8; cat. #555367, 15 µl), anti-CD20 (clone 2H7; cat. #555623, 15 µl), from BD and anti-CD159a (clone Z199; cat. #IM3291U, 10 µl).

Validation

All the antibodies used are reported as cross-reactive with the Monkey either on Reactivity database of the Nonhuman Primate Reagent Resource (NHPRR) website (https://www.nhpreagents.org/) or by the manufacturers' websites.

Antibodies cross-reactivity with Rhesus macaques: for clones SP34-2, 2H7, D058-1283, M5E2, RPA-T8 and 3.9, cross-reactivity reported on BD Biosciences website; for clones 3G8, 48607, 12G5, B-Ly7, 3D12, AC104.3E3, GHI/61, 1A4, MIH1, AD2, SK9, 2331 (FUN-1), TET2, Z199 and 2D10.4, cross-reactivity reported on Nonhuman Primate Reagent Resource website (https://www.nhpreagents.org/); for clones L243, 2A9-1, ICRF44, and L161, cross-reactivity reported on BioLegend website.

Animals and other research organisms

Policy information about studies involving animals; ARRIVE guidelines recommended for reporting animal research, and Sex and Gender in Research

Laboratory animals

All animals used in these studies were Indian rhesus macaques (Macaca mulatta) obtained from Alpha Genesis Inc. (Yemasee, SC), Primate Products Inc. (Immokalee, FL), the National Institute of Child Health and Human Development (NICHD, Rockville, MD), PreLabs (Hines, IL), SNBL (Everett, WA), Worldwide Primates Inc. (Miami, FL), New Iberia Research Center (New Iberia, LA), and Covance Inc. (Princeton, NJ).
Study 1. Forty juvenile (young, average age 3.45 years and 0.545 SD) and twenty-eight adult (old, average age 7.98 years and 2.60 SD) female macaques
Study 2. Twelve juvenile (average age 3.91 years and 0.155 SD) female macaques

Wild animals

The study did not involve wild animals

Reporting on sex

All animals used in these studies were female. The aim included evaluating the vaccine efficacy following vaginal exposure, therefore the sex of the animals was female

Field-collected samples

The study did not involve samples collected from the field

Ethics oversight

All animals were handled in accordance with the standards of the Association for the Assessment and Accreditation of Laboratory Animal Care (AAALAC) in an AAALAC-accredited facility (OLAW, Animal Welfare Assurance A4149-01 for NIH and A3086-01 for Bioqual). All animal care and procedures were carried out under protocols approved by the NCI and/or NIAID Animal Care and Use Committees (ACUC; Protocol numbers: VB-013 and VB-026 at the NIH and P-181 at Bioqual).

Note that full information on the approval of the study protocol must also be provided in the manuscript.

Flow Cytometry

Plots

Confirm that:

- [x] The axis labels state the marker and fluorochrome used (e.g. CD4-FITC).
- [x] The axis scales are clearly visible. Include numbers along axes only for bottom left plot of group (a 'group' is an analysis of identical markers).
- [x] All plots are contour plots with outliers or pseudocolor plots.
- [x] A numerical value for number of cells or percentage (with statistics) is provided.

Methodology

Sample preparation	To identify monocytic myeloid cells in Study 1, cryopreserved PBMCs (5-10x10^6 cells) collected following the ALVAC-SIV +gp120 immunization (week 13) were thawed and stained with Fluorochrome-conjugated mAbs.<br>To identify monocytic myeloid cells in Study 2, cryopreserved PBMCs (5-10x10^6 cells) collected at baseline and following the ALVAC-SIV+gp120 ΔV1 immunization (week 13) were thawed and stained with Fluorochrome-conjugated mAbs.
Instrument	Flow cytometry acquisitions for Study 1 were performed on an LSRII and examined using FACSDiva software (BD Biosciences) by acquiring a minimum of 500,000 events for myeloid cell evaluation. Flow cytometry acquisitions for Study 2 were performed on a FACSymphony A5 and examined using FACSDiva software (BD Biosciences) by acquiring all stained cells.
Software	FACSDiva software (BD Biosciences) and FlowJo v10.1 (TreeStar, Inc., Ashland, OR)
Cell population abundance	For the identification of monocytic myeloid cells the analyses were conducted on Ficoll isolated PBMCs.<br><br>For ATAC-seq and RNA-seq in CD14+ cells of Study 2 the purity of CD14+ cells was assessed by flow cytometry. Following isolation, the purity was assessed using the following antibodies: PE-Cy7 anti-CD20 (clone 2H7; cat. #560735, 2.0 µl), Alexa700 anti-CD3 (clone SP34-2; cat. # 557917, 5.0 µl), APC anti-CD14 (clone M5E2; cat. #555399, 7.0 µl), FITC anti-CD16 (clone 3G8; cat. #555406, 5.0 µl) all from BD Biosciences (San Jose, CA) and Violet LIVE/DEAD viability dye (cat. # L34955, 1 µl; Thermo Fisher Scientific, Waltham, MA) purity ranged between 60-98% of live cells (Gating strategy Singlets/Live/CD3-CD20-/CD14+CD16-)<br><br>For Efferocytosis assay the purity of CD14+ cells and Neutrophils were not assessed due to low cell availability. The same isolation methods of the 2 types of cells were repeatedly used in previous experiments and the purity ranged between 60-98% of live cells for CD14+ cells (Gating strategy Singlets/Live/CD3-CD20-/CD14+CD16-) and between 75-92% of CD45+ cells for Neutrophils (Gating strategy Singlets/Live/CD45+/CD3-CD20-CD8-/CD66abce+).<br>- CD14+: cells purity was assessed in previous experiments by flow cytometry and the following antibodies were used: PE-Cy7 anti-CD20 (clone 2H7; cat. #560735, 2.0 µl), Alexa700 anti-CD3 (clone SP34-2; cat. # 557917, 5.0 µl), APC anti-CD14 (clone M5E2; cat. #555399, 7.0 µl), FITC anti-CD16 (clone 3G8; cat. #555406, 5.0 µl) all from BD Biosciences (San Jose, CA) and Violet LIVE/DEAD viability dye (cat. # L34955, 1 µl; Thermo Fisher Scientific, Waltham, MA)<br>- Neutrophils: +: cells purity was assessed in previous experiments by flow cytometry and the following antibodies were used: Alexa700 anti-CD3 (clone SP34-2; cat. # 557917, 3.0 µl), Alexa700 anti-CD20 (clone 2H7; cat. #560631, 3.0 µl), Alexa700 anti-CD8 (clone RPA-T8; cat. # 561453, 3.0 µl), BV786 anti-CD45 (clone D058-1283; cat. #563861, 3.0 µl) all from BD Biosciences (San Jose, CA); FITC anti-CD66abce (clone TET2; cat. #130-116-668, 2.0 µl) from Miltenyi Biotec (Cambridge, MA) and Blue LIVE/DEAD viability dye (cat. #L34962, 0.5 µl; Thermo Fisher Scientific).
Gating strategy	Monocyte populations were identified as CD3-CD20-CD45+HLA-DR+ and differentiated by the expression of CD14 and CD16, as previously published. Classical monocytes were identified as CD14+CD16-, Intermediate as CD14+CD16+, and Non-Classical as CD14–CD16+. Monocyte subsets were expressed either as frequency of the parental HLA-DR+ gate or the live cells. The expression of CCR2 in each monocytic subset was expressed as the frequency of CCR2+ cells in their parental subset. DC-10 cells were identified as either Myeloid cells (high SSC)/Singlets/Live CD45+/CD3-CD20-/HLA-DR+/ CD1c-/CD11b+/CD11c+/ CD14+CD16+/CD163+/CD141+/CD1a-) and expressed as the frequency of the cells in the final gate (CD1a-) of the cells in CD11b+ gate. Boundaries between positive and negative cells were established by the neat separation of the 2 major populations when the abundance of the cells was allowing the distinction, or by the use of FMO (Fluorescence minus one) when the cellular marker was had low expression or the positive populations were rare<br>A figure exemplifying the gating strategy is provided in the Supplementary Information only for Efferocytosis assay and DC-10 evaluation conducted in Study 2

- [x] Tick this box to confirm that a figure exemplifying the gating strategy is provided in the Supplementary Information.
